# MicroRNA-21 guide and passenger strand regulation of adenylosuccinate lyase-mediated purine metabolism promotes transition to an EGFR-TKI-tolerant persister state

**DOI:** 10.1038/s41417-022-00504-y

**Published:** 2022-07-15

**Authors:** Wen Cai Zhang, Nicholas Skiados, Fareesa Aftab, Cerena Moreno, Luis Silva, Paul Joshua Anthony Corbilla, John M. Asara, Aaron N. Hata, Frank J. Slack

**Affiliations:** 1 Harvard Medical School Initiative for RNA Medicine, Department of Pathology, Cancer Center, Beth Israel Deaconess Medical Center, Harvard Medical School, Boston, MA 02215 USA; 2 Department of Cancer Division, Burnett School of Biomedical Sciences, College of Medicine, University of Central Florida, 6900 Lake Nona Blvd, Orlando, FL 32827 USA; 3 Department of Medicine, Division of Signal Transduction, Beth Israel Deaconess Medical Center, Harvard Medical School, Boston, MA 02215 USA; 4 Department of Medicine, Massachusetts General Hospital, Charlestown, MA 02129 USA

**Keywords:** Cancer genetics, Cancer genetics

## Abstract

In EGFR-mutant lung cancer, drug-tolerant persister cells (DTPCs) show prolonged survival when receiving EGFR tyrosine kinase inhibitor (TKI) treatments. They are a likely source of drug resistance, but little is known about how these cells tolerate drugs. Ribonucleic acids (RNAs) molecules control cell growth and stress responses. Nucleic acid metabolism provides metabolites, such as purines, supporting RNA synthesis and downstream functions. Recently, noncoding RNAs (ncRNAs), such as microRNAs (miRNAs), have received attention due to their capacity to repress gene expression via inhibitory binding to downstream messenger RNAs (mRNAs). Here, our study links miRNA expression to purine metabolism and drug tolerance. MiR-21-5p (guide strand) is a commonly upregulated miRNA in disease states, including cancer and drug resistance. However, the expression and function of miR-21-3p (passenger strand) are not well understood. We found that upregulation of miR-21-5p and miR-21-3p tune purine metabolism leading to increased drug tolerance. Metabolomics data demonstrated that purine metabolism was the top pathway in the DTPCs compared with the parental cells. The changes in purine metabolites in the DTPCs were partially rescued by targeting miR-21. Analysis of protein levels in the DTPCs showed that reduced expression of adenylosuccinate lyase (ADSL) was reversed after the miR-21 knockdown. ADSL is an essential enzyme in the de novo purine biosynthesis pathway by converting succino-5-aminoimidazole-4-carboxamide riboside (succino-AICAR or SAICAR) to AICAR (or acadesine) as well as adenylosuccinate to adenosine monophosphate (AMP). In the DTPCs, miR-21-5p and miR-21-3p repress ADSL expression. The levels of top decreased metabolite in the DTPCs, AICAR was reversed when miR-21 was blocked. AICAR induced oxidative stress, evidenced by increased reactive oxygen species (ROS) and reduced expression of nuclear factor erythroid-2-related factor 2 (NRF2). Concurrently, miR-21 knockdown induced ROS generation. Therapeutically, a combination of AICAR and osimertinib increased ROS levels and decreased osimertinib-induced NRF2 expression. In a *MIR21* knockout mouse model, *MIR21* loss-of-function led to increased purine metabolites but reduced ROS scavenging capacity in lung tissues in physiological conditions. Our data has established a link between ncRNAs, purine metabolism, and the redox imbalance pathway. This discovery will increase knowledge of the complexity of the regulatory RNA network and potentially enable novel therapeutic options for drug-resistant patients.

## Introduction

Nucleic acids including deoxyribonucleic acid (DNA) and ribonucleic acid (RNA) are macromolecules that are central to all biological functions [1]. Nucleic acid molecules consist of purine and pyrimidine nucleosides [2]. Purine and pyrimidine nucleic acids include adenosine (A), guanosine (G), as well as cytosine (C), thymine (T), and uracil (U). The process of purine nucleoside metabolism is mainly catalyzed by metabolic enzyme activities leading to the creation of adenosine monophosphate (AMP) [3]. For example, adenylosuccinate lyase (ADSL) is an essential enzyme involved in purine metabolism. It catalyzes two reactions in the de novo purine biosynthetic pathway: the conversion of succino-5-aminoimidazole-4-carboxamide riboside (succino-AICAR or SAICAR) to AICAR (or acadesine) and the conversion of adenylosuccinate (S-AMP) to AMP [4]. The metabolic enzymes mediating purine metabolism are finely controlled at the transcriptional or post-translational level [5]. For example, the mechanistic target of rapamycin complex 1 (mTORC1) regulates methylenetetrahydrofolate dehydrogenase 2 (MTHFD2) enzyme at the transcriptional level contributing to purine synthesis [6]. However, it is not clear how the processes for purine metabolism are controlled after genetic transcription [7]. Thus, it is interesting to understand which molecules are involved in purine biosynthesis at the post-transcriptional level.

Small noncoding RNAs such as microRNAs (miRNAs) tune gene expression via regulating protein degradation and RNA stability [8, 9]. In miRNA biosynthesis, transcription generates a primary miRNA (pri-miRNA) that is subsequently processed to produce a precursor miRNA (pre-miRNA) that generates two potentially active miRNAs, the guide and passenger strand, or 5p and 3p strand [10]. Recently, miRNAs have received increased attention due to their modulating metabolic pathways [11, 12]. For example, a low oxygen-induced miRNA, miR-210 represses succinate dehydrogenase (SDH) complex subunit D expression leading to dysregulation of the tricarboxylic acid (TCA) cycle and electron transfer chain [13]. miR-147b regulates VHL and SDH complex in response to hypoxia [14]. Reduced miR-200c de-represses lactate dehydrogenase A resulting in increased lactate production and enhanced aerobic glycolysis [15]. Furthermore, miRNAs regulate nucleic acid synthesis. For instance, nuclear factor erythroid-2-related factor 2 (NRF2) activation-induced downregulation of miR-1 and miR-206 derepress glucose-6-phosphate dehydrogenase, phosphogluconate dehydrogenase, and transketolase. These enzymes increased nucleic acid synthesis by providing a ribose [16]. In addition, miRNA expression levels can distinguish various diseases, including cancer [17–19]. miRNAs can be used as therapeutic targets alone or combined with small molecules [20–25]. Given this precedence, it is critical to understand if miRNAs modulate purine synthesis and metabolism in a disease-dependent context.

Wild-type epidermal growth factor receptor (*EGFR*) is critical to maintaining normal cell growth and organ development [26]. Conversely, somatic *EGFR* mutations drive epithelial cell-derived tumors such as lung and other types of cancer [27, 28]. To block mutation-induced EGFR overactivation, EGFR tyrosine kinase inhibitors (TKIs) including gefitinib and erlotinib, have been applied clinically. By binding to the EGFR adenosine triphosphate (ATP) binding pocket, gefitinib/erlotinib blocks constitutive EGFR phosphorylation and its downstream phosphoinositide 3-kinase (PI3K)/AKT serine/threonine kinase (AKT) and RAS/extracellular signal-regulated kinase (ERK) signaling pathways [29]. However, about half of the acquired resistant cases to gefitinib are caused by an *EGFR Thr790Met* (*T790M*) mutation increasing the affinity for ATP [30]. Osimertinib, a 3rd generation of EGFR TKI, can overcome acquired *T790M* [31], and has also demonstrated superior activity as a first-line treatment for *EGFR*-mutant NSCLC with activating *del19* and *L858*R mutation in the absence of *T790M* [32]. However, acquired drug resistance against osimertinib occurs inevitably through either gain of new genetic mutations such as *C797S*, *MET,* and other gene amplifications, and lineage transformation to small cell or squamous cell lung cancer [33–35]. Drug-tolerant persister cells (DTPCs) are a population of malignant cells showing prolonged survival when receiving anticancer treatments and are a likely source of drug resistance [36, 37]. Our previous study showed that the dysregulated TCA cycle in mitochondria linked the pseudohypoxia signaling pathway to the drug-tolerant persister (DTP) state acquisition in *EGFR*-mutant lung cancer [14, 38]. Furthermore, recent studies showed that activated EGFR signals shunt glycolysis to serine synthesis for nucleotide biosynthesis [39], and modulate tumor-suppressive miRNA biogenesis in hypoxia [40]. Blocking EGFR signals with EGFR TKIs reduced miRNA expression of such genes as miR-221~222, miR-30b~c [41], and miR-21 [42]. Further knocking down these miRNAs enhanced EGFR-TKI-induced apoptosis in *EGFR*-mutant lung cancer cells [43]. In addition, cellular metabolites, such as lactate, cause the drug resistance to anti-EGFR therapy [44]. This suggests that dysregulated metabolic pathways and miRNAs moderate tumor cells’ growth and adaptation to EGFR TKI in *EGFR*-driven cancer. However, how tumor cells transit to the DTP state remains unclear via interaction between miRNAs and metabolic pathways. In this study, we discovered for the first time that dysregulated purine metabolism contributes to the acquisition of the DTP state, regulated by the miR-21 guide and passenger strand repressing ADSL in *EGFR*-mutant lung cancer cells.

## Results

### MiR-21 abundance contributes to the transition to a drug-tolerant persister state

We leveraged an unbiased small RNA-sequencing (RNA-seq) approach in *EGFR*-mutant PC9 cells from an independent lab [45] to profile miRNA expression patterns. We found that miR-21-5p is the most abundant miRNA, accounting for ~20% of the reads among 2592 miRNA candidates (Supplementary Fig. 1A). It is consistent with a previous report showing that EGFR activation induces miR-21 upregulation [42].

Numerous preclinical and clinical studies have demonstrated that activated oncogenic EGFR was vulnerable to EGFR TKIs [29, 31, 46]. This therapeutic success was compromised by the emergence of inevitable drug resistance after the initial treatment response, suggesting a critical need to understand the mechanisms of drug resistance. It was reported that miR-21 overexpression increased resistance to EGFR-TKI gefitinib [47, 48]. These discoveries indicate that the mechanism of miR-21 in response to EGFR TKI might be EGFR-independent. Mature duplex miR-21 yields two miRNAs after separating its two complementary strands into a guide strand [5p] and a passenger strand [3p]. One conventional concept of miRNA biogenesis is that the guide strand is functional, but the passenger strand loses its function due to degradation during the processing step [49]. Since a study has shown a role for the miR-21 passenger strand in carcinogenesis [50], we were curious if the passenger strand of miR-21 was stable during the pathogenesis of drug response and resistance. Surprisingly, miR-21-3p is in the top expressed miRNAs that account for 1% of the total reads in PC9 cells. With treatment of a 3rd generation EGFR inhibitor WZ4002 that shares many common structural features with osimertinib [51] for 24 h, our analysis showed that the normalized reads for miR-21-5p increased 32.6% while miR-21-3p reads remained stable in parental PC9 cells after treatment (Supplementary Fig. 1B). However, in DTPCs derived from PC9, miR-21-3p reads increased by 14.4%, but miR-21-5p reads did not increase (Supplementary Fig. 1B). When the DTPCs were rechallenged with WZ4002 for 48 h, the sequence reads for miR-21-5p increased and miR-21-3p reads decreased slightly (Supplementary Fig. 1B). This data indicates that the expression of miR-21-3p and miR-21-5p are dynamic during drug treatment.

**Fig. 1:**
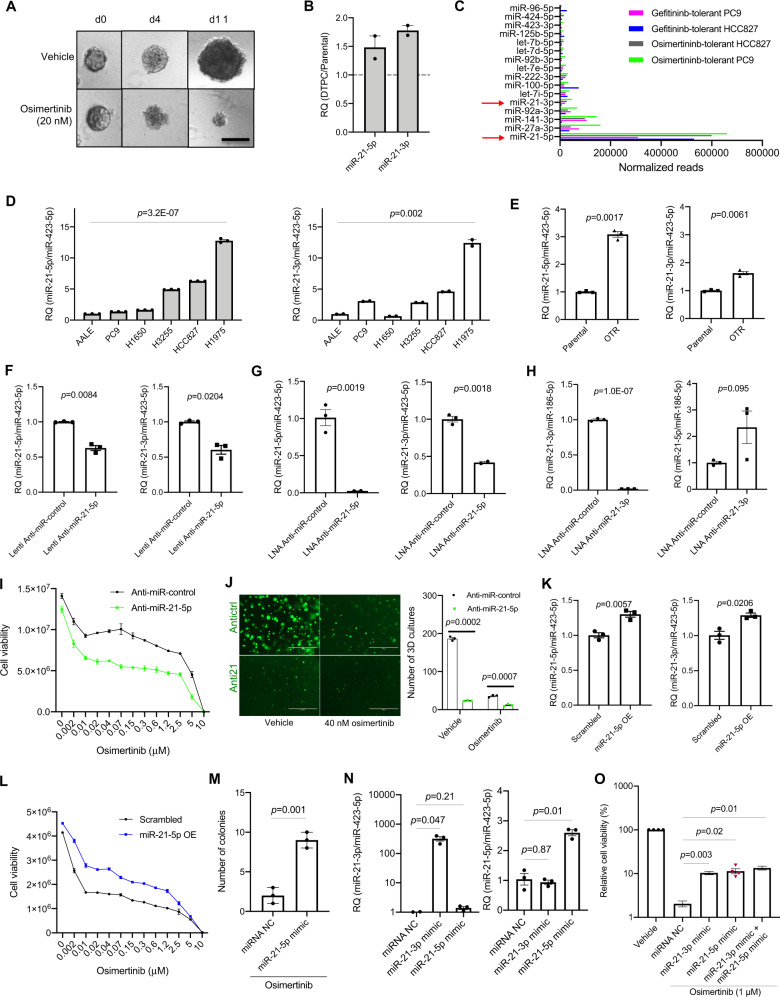
miR-21 regulates drug tolerance to EGFR tyrosine kinase inhibitors. **A** Representative images of establishing drug-tolerant persister cells (DTPCs) from HCC827 in 3D cultures in the presence of osimertinib. The 3D cultures were continuously treated with 20 nM osimertinib or vehicle, and images were taken on days 0, 4, and 11. Scale bar, 100 µm. **B** Relative expression of miR-21-3p and miR-21-5p in DTPCs compared with that in parental cells from PC9 and HCC827. *N* = 2 replicates. **C** Normalized reads for top expressed miRNAs by small RNA-seq analysis across gefitinib-tolerant and osimertinib-tolerant cells from PC9 and HCC827. →, highlighting miR-21-5p and miR-21-3p. **D** qRT-PCR analysis for miR-21-5p and miR-21-3p expression across an immortalized lung epithelial cell (AALE) and EGFR-mutant lung cancer cell lines from humans. miR-423-5p was used as an endogenous control. *N* = 2–3 replicates. **E** qPCR analysis for miR-21-5p and miR-21-3p expression in parental and osimertinib-tolerant (OTR) cells from H1975. miR-423-5p was used as an endogenous control. *N* = 3 replicates. **F** qPCR analysis for miR-21-5p and miR-21-3p expression in H1975 parental cells treated with a lentiviral miR-21-5p inhibitor. The cells were infected with lenti miRa-Off-hsa-miR-21-5p virus (lenti anti-miR-21-5p) or a control inhibitor (lenti anti-miR-control) and selected with 0.5 µg/ml puromycin for 3 days. Then RNAs were extracted for qPCR analysis. miR-423-5p was used as an endogenous control. *N* = 3 replicates. **G** qPCR analysis for miR-21-5p and miR-21-3p expression in H1975 cells treated with the locked nucleic acid (LNA) miR-21-5p inhibitor. The cells were transfected with the LNA miR-21-5p inhibitor (LNA anti-miR-21-5p) or a control inhibitor (LNA anti-miR-control), followed by RNA extraction 48 h post-transfection and qPCR assay. miR-423-5p was used as an endogenous control. *N* = 2–3 replicates. **H** qPCR analysis for miR-21-5p and miR-21-3p in H1975 cells treated with the LNA miR-21-3p inhibitor and a miRNA control inhibitor 48 h post-transfection. miR-186-5p was used as an endogenous control. *N* = 3 replicates. **I** Osimertinib treatment response on H1975 cells infected with the GFP-labeled lentiviral miR-21 inhibitor (anti-miR-21-5p) or scrambled control (anti*-*miR-control). The cell viability was measured with the CellTiter-Glo luminescent cell viability assay on day 4 after treatment with serially diluted osimertinib. *N* = 3 replicates. **J** Osimertinib treatment response on H1975 cells in 3D cultures with the GFP-conjugated lentiviral miR-21 inhibitor (anti-miR-21-5p) and scrambled control (anti-miR-control). The 3D structures were treated with 40 nM osimertinib for 14 days. Images were taken under an EVOS fluorescent microscope. The number of 3D cultures was quantified and analyzed. Scale bar, 1000 µm. *N* = 3 replicates. **K** qPCR analysis for miR-21-5p and miR-21-3p expression in HCC827 cells. The cells were infected with the lentiviral miRa-GFP-hsa-miR-21-5p virus (miR-21-5p OE) or scrambled control and selected with 0.5 µg/ml puromycin for 3 days. Then RNAs were extracted for qPCR analysis. miR-423-5p was used as an endogenous control. *N* = 3 replicates. **L** Osimertinib treatment response on HCC827 cells infected with the lentiviral miR-21-5p (miR-21-5p OE) plasmid or scrambled control. The cell viability was measured with the CellTiter-Glo luminescent cell viability assay on day 4 after treatment with serially diluted osimertinib. *N* = 3 replicates. **M** Colony formation assay for HCC827 cells infected with the lentiviral miR-21 plasmid (miR-21-5p OE) and scrambled control in 40 nM osimertinib. The number of colonies formed within 14 days was quantified and analyzed. *N* = 3 replicates. **N** qPCR analysis for miR-21-5p and miR-21-3p expression in HCC827 cells treated with 40 nM LNA miR-21-3p mimic, miR-21-5p mimic and a negative miRNA mimic control (NC). miR-423-5p was used as an endogenous control. *N* = 3 replicates. **O** Osimertinib treatment response on HCC827 cells transfected with the LNA miR-21-3p mimic, miR-21-5p mimic, and a negative miRNA mimic control in the presence or absence of osimertinib. Twenty-four hours post-transfection with the 40 nM miRNA mimic, the cells were treated with 1 μM osimertinib for 6 days, followed by CellTiter-Glo luminescent cell viability assay. *N* = 4 replicates. Data are mean ± s.e.m. and were analyzed with an unpaired two-tailed *t-*test (**D**, **F**, **G**, **H**); unpaired two-tailed *t*-test with Welch’s correction (**E**, **K**, **M**); Kruskal–Wallis ANOVA (**J**); Brown–Forsythe and Welch ANOVA (**N**, **O**).

Then we asked if the roles of miR-21 in response to EGFR TKIs were similar between growth in two-dimensional (2D) monolayer and 3D cultures. To address this question, we established DTPCs from parental PC9 and HCC827 cells by treating them with 20 nM osimertinib continuously for 2 weeks in 3D cultures (Fig. 1A). Then we performed a small RNA-seq analysis to compare miR-21-5p and miR-21-3p expression between DTPCs and parental cells. We found that miR-21-5p and miR-21-3p expression levels increased 1.5 and 1.8-fold, respectively (Fig. 1B). Consistently, miR-21-3p was abundant compared to most other miRNAs, although the reads for miR-21-3p were lower than those of miR-21-5p in PC9 and HCC827 cells (Fig. 1C and Supplementary Table 1). Thus, these data confirmed that miR-21-5p and miR-21-3p expression increased in DTPCs in both 2D and 3D cultures.

To further explore if miR-21 is functional in transition from a drug-sensitive state to a DTP state, we performed a small-scale expression screening of miR-21-5p and miR-21-3p in a panel of human lung cell lines. They include five *EGFR*-mutant lung adenocarcinoma cell lines and one immortalized lung epithelial cell line AALE (Supplementary Table 2). Our data demonstrated that H1975 was the cell line with the highest expression level of miR-21-5p and miR-21-3p (Fig. 1D). We then established osimertinib-tolerant (OTR) cells by continuously treating parental H1975 cells with 100 nM osimertinib for 2 weeks. Our qPCR analysis showed that the expression for both miR-21-5p and miR-21-3p increased in OTR cells compared with parental cells from H1975 (Fig. 1E).

Next, we knocked down miR-21 in H1975 cells with a lentiviral vector targeting miR-21-5p to evaluate the roles of miR-21-5p and miR-21-3p in cells treated with osimertinib (Supplementary Fig. 2A). The real-time PCR analysis demonstrated that miR-21-5p expression was downregulated 40% in cells when miR-21-5p was knocked down compared with control cells. Surprisingly, expression for miR-21-3p also decreased by 40% in miR-21-5p knockdown cells compared with control cells (Fig. 1F), possibly due to an effect on pre-miRNA processing. To further understand the interactive regulation between the guide and passenger strand, we used a locked nucleic acid (LNA)-modified oligonucleotide synthetic inhibitor against miR-21-5p or miR-21-3p to transfect H1975 cells for 48 h. Our qPCR data showed that expression levels for miR-21-5p and miR-21-3p decreased by 97% and 58% in cells treated with the miR-21-5p inhibitor, respectively (Fig. 1G). Only miR-21-3p expression levels were reduced by 98%, but miR-21-5p levels were not changed significantly in cells treated with the miR-21-3p inhibitor (Fig. 1H). To exclude an off-targeting mechanism from occurring, a perfect complementarity between nucleotide position 2–7 or 2–8 (seed region) of the antisense strand and the 3’ untranslated region (UTR) of the transcript is necessary [52–54]. Consistently, the sequence analogy analysis showed that the LNA miR-21-5p inhibitor could bind to the seed region of miR-21-5p rather than miR-21-3p (Supplementary Fig. 2B). Similarly, the LNA miR-21-3p inhibitor can only bind to the seed region of miR-21-3p rather than miR-21-5p (Supplementary Fig. 2C). Furthermore, we tested a serial dilution of LNA miR-21-5p inhibitor in H1975 cells to determine its specificity in targeting miR-21-5p. Our qPCR data showed that as low as 10 nM LNA inhibitor decreased miR-21-5p expression by 59%. The maximum reduction was from the cells treated with 120 nM miR-21-5p inhibitor (Supplementary Fig. 2D). These data confirm the specificity of miRNA inhibitors. We inferred that miR-21-5p inhibitor might disturb the post-transcriptional regulation of pre-miR-21, leading to downregulation of miR-21-3p. These data indicate that blocking miR-21-5p with the LNA inhibitor might inhibit the cleavage of pre-miRNA into miRNA duplex or miRNA duplex formation through negative feedback. Consequently, the passenger strand [3p] was reduced after defects in miRNA duplex formation. This hypothesis was supported by our qPCR analyses showing upregulated pre-miR-21 (Supplementary Fig. 3) and downregulated miR-21-3p expression after the cells were treated with the LNA miR-21-5p inhibitor. This indicates that miR-21-5p impacts the regulation of miR-21-3p expression but that miR-21-5p expression is less affected by miR-21-3p.

Our drug response results showed that the lentiviral miR-21-5p inhibitor increased drug sensitivity to osimertinib in short-term 2D assays (Fig. 1I) and reduced drug tolerance in long-term 3D cultures (Fig. 1J). Similarly, both LNA miR-21-5p inhibitor and LNA miR-21-3p inhibitor increased treatment response to osimertinib in parental H1975 cells in monolayer cultures compared to the LNA control inhibitor treatment (Supplementary Fig. 4). Upregulation of miR-21-5p and miR-21-3p with 1.3-fold change was found in HCC827 cells infected with a lentiviral vector containing mature miR-21-5p sequence (Fig. 1K). Cells overexpressing both miR-21-5p and miR-21-3p increased drug tolerance (Fig. 1L) and single-cell-derived colony formation within 14 days by clonogenicity assay in HCC827 cells treated continuously with 40 nM osimertinib (Fig. 1M).

To further understand miR-21-3p and miR-21-5p in acquiring the DTP state in lung cancer cells, we overexpressed synthetic miR-21-3p and miR-21-5p mimics in HCC827 cells. qPCR data showed that miR-21-3p and miR-21-5p expression levels increased 320-fold and 2.6-fold in miR-21-3p mimic and miR-21-5p mimic-expressing cells. Neither miR-21-3p expression changed in miR-21-5p mimic-expressing cells nor miR-21-5p expression changed in miR-21-3p mimic-expressing cells (Fig. 1N). We compared the drug response in HCC827 cells treated with synthetic LNA miR-3p mimic and miR-21-5p mimic for 6 days and measured cell survival in the absence or presence of 1 μM osimertinib. Our drug response data showed that both miR-21-3p mimic and miR-21-5p mimic partially rescued osimertinib-induced cell decrease leading to increased drug tolerance (Fig. 1O).

Thus, our data have demonstrated that both miR-21-3p and miR-21-5p regulate the transition to a DTP state in lung cancer cells.

### MiR-21 regulates purine metabolism leading to a DTP state

Previous studies showed that miR-21-5p induced drug resistance to the 1st generation of EGFR-TKI gefitinib through decreasing expression of tumor suppressor phosphatase and tensin homolog (PTEN) or programmed cell death protein 4 (PDCD4) in gefitinib/erlotinib-resistant PC9 (PC9ER) cells [47, 55]. We found that knocking down miR-21-5p with 120 nM of LNA inhibitor did not increase the expression of PTEN and PDCD4 (*P* = 0.92 and *P* = 0.73, respectively) in PC9ER cells. Knocking down miR-21-3p with 120 nM of LNA inhibitor increased the expression of PTEN (*P* = 0.012) (Supplementary Fig. 5A). In H1975 cells, LNA miR-21-3p inhibitor treatment reduced PTEN expression (*P* = 0.008) but increased PDCD4 expression (*P* = 0.018). Treatment with the miR-21-5p inhibitor did not significantly change PTEN or PDCD4 expression in H1975 cells (Supplementary Fig. 5B). Thus, our data have demonstrated that blocking miR-21-3p rather than miR-21-5p increased PTEN and PDCD4 expression in PC9ER and H1975 cells, respectively. This indicates that the mechanisms of miR-21-5p and miR-21-3p in regulating PTEN and PDCD4 are cell context-dependent.

**Fig. 2:**
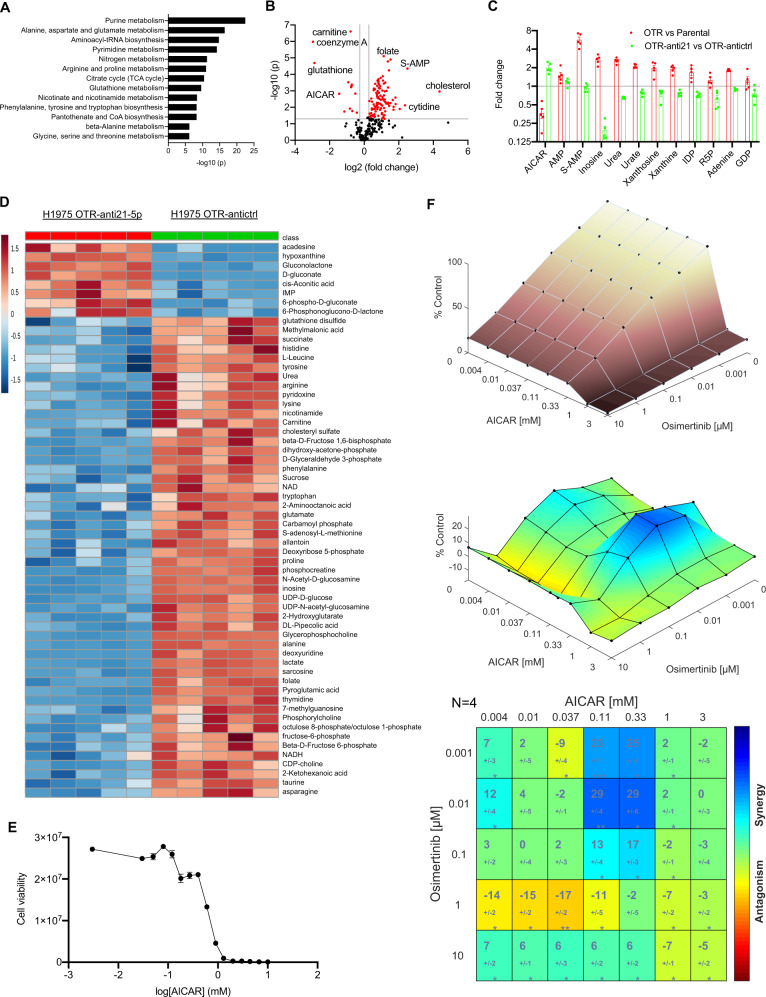
miR-21 regulates purine metabolism leading to a DTP state. **A** A bar chart showing the top metabolic pathways in osimertinib-tolerant cells (OTR) compared to parental cells from H1975. *N* = 5 replicates. **B** A volcano plot showing dysregulated metabolites in OTR cells compared to parental cells from H1975. The red dots represent metabolites above the threshold with fold change >1.2 and *P* < 0.05. Names of top dysregulated metabolites were denoted. *N* = 5 replicates. **C** Reciprocal changes of metabolites in the purine metabolism pathway in two comparisons including OTR vs. parental cells, and OTR-anti-21-5p vs. OTR-antictrl from H1975. **D** A heatmap showing the top upregulated and downregulated metabolites in H1975 OTR cells with a lentiviral miR-21-5p inhibitor and a scrambled control. *N* = 5 replicates. **E** AICAR treatment response on H1975 cells for 3 days. *N* = 3 replicates. **F** A synergistic analysis of combination treatment with AICAR and osimertinib in H1975 cells. 3000 cells were plated in 96-well plates and incubated with AICAR (0–3 mM), osimertinib (0–10 µM), combo, or vehicle control for 3 days. Cell viability was measured with Celltiter-Glo, and the synergy was analyzed with Combenefit using the Bliss independence model. All samples were normalized to a vehicle control group. A dose–response surface curve (top), a surface isogram (middle), and a matrix table (bottom) were created for each combination. The matrix plot shows the maximum % synergy score obtained by comparing experimental combination dose–response values with Bliss-model values, with standard deviation indicated below. (*P < 0.05; **P < 0.01, ***P < 0.001). *N* = 4 replicates. Data are mean ± s.e.m. unless stated otherwise and were analyzed with unpaired two-tailed *t*-test (**A**, **B**, **D**) and one-sample *t*-test (**F**).

Metabolic changes tune drug response in current anticancer therapies [56]. We hypothesized that treatment-induced metabolite dysregulation could regulate the transition from the drug-sensitive state to the DTP state. First, we asked if metabolome changes could distinguish DTPCs from parental cells. We performed a metabolomics analysis of parental and DTPCs from H1975 with a liquid chromatograph-mass spectrometry (LC-MS) tool to address this question. The top dysregulated metabolic pathways in comparing DTPCs and parental cells were purine metabolism, alanine, aspartate and glutamate metabolism, aminoacyl-tRNA biosynthesis, pyrimidine metabolism, nitrogen metabolism, arginine, and proline metabolism as well as citrate cycle (Fig. 2A). Recent studies showed that purine metabolism is linked to liver tumor growth [57] and glioblastoma radiation resistance [58]. Our data indicate that purine metabolism is relevant to acquiring drug tolerance to EGFR TKIs in lung cancer.

Our analysis showed that the top upregulated metabolites in DTPCs versus parental cells were cholesterol, adenylosuccinate (also called succinyladenosine monophosphate or S-AMP), cytidine, acetyl coenzyme A (acetyl-CoA), and N-acetyl-glutamate (Fig. 2B and Supplementary Table 3). The top downregulated metabolites were CoA, glutathione, aminoimidazole carboxamide riboside (AICAR or acadesine), and S-adenosylhomocysteine (SAH) (Fig. 2B and Supplementary Table 4).

Among the list of top upregulated metabolites, S-AMP is an intermediate in the interconversion of purine nucleotides inosine monophosphate (IMP) and adenosine monophosphate (AMP) catalyzed by ADSL [4]. Cytidine is a pyrimidine nucleoside molecule that is found in RNA. Acetyl-CoA delivers the acetyl group to the tricarboxylic acid cycle to be oxidized for ATP generation. N-acetyl-glutamate is biosynthesized from acetyl-CoA or acetylornithine and is involved in the urea cycle (Supplementary Table 3).

Among another list of top downregulated metabolites, CoA is involved in fatty acid biosynthesis and ATP production. Glutathione is an antioxidant and can overcome reactive oxygen species (ROS)-induced cellular damage [59]. AICAR is an intermediate metabolite in purine de novo biosynthesis and mimics AMP to stimulate AMP-activated protein kinase (AMPK) [60, 61]. SAH is derived from S-adenosyl-l-methionine (SAM) and is converted into homocysteine and adenosine. Besides AMP, S-AMP, and AICAR in the purine metabolism synthesis pathway [62], other metabolites in the purine degradation pathway also show significant changes, including upregulated inosine, urea, urate, xanthosine, xanthine, inosine diphosphate (IDP), adenine, and guanosine diphosphate (GDP) (Fig. 2B, C and Supplementary Table 4). The high levels of purine degradation products might be related to the increased recycling of these degraded purines into building blocks for the DNA and RNA synthesis [63].

Then we asked if the metabolic changes in DTPCs were rescued by miR-21 perturbation. Because the lentiviral miR-21-5p inhibitor could decrease expression of both miR-21-5p and miR-21-3p to a similar extent, we introduced this vector in OTR cells from H1975 and performed metabolomic analysis. Interestingly, acadesine, the non-phosphorylated form of AICAR was the most upregulated metabolite after miR-21 knockdown (Fig. 2D). Similarly, reduced AICAR level in OTR cells was also upregulated twofold upon miR-21 knockdown (Supplementary Table 5). All the other metabolites in purine metabolism consistently demonstrated partially reversed changes after miR-21 knockdown in DTPCs (Fig. 2C). Our data confirmed a link between miR-21 and purine metabolism in lung cancer cells.

We asked if AICAR treatment could prevent miR-21-induced transition to the DTP state to test if this was a functional link. We added AICAR in parental H1975 cells with or without osimertinib and performed a drug response assay. Our results showed that AICAR monotherapy of less than 0.12 mM did not decrease cell viability obviously (Fig. 2E). Using the Bliss independence model [64], AICAR and osimertinib showed synergistic effects, with a 29 ± 4% increase in drug sensitivity in H1975 cells treated with 0.01 µM osimertinib and 0.11 mM AICAR compared to predicted levels (Fig. 2F). This suggests that AICAR can further increase osimertinib-induced drug response. Our data have revealed that the miR-21-induced DTP state is via a reduction of AICAR in purine metabolism.

### ADSL is targeted by MiR-21 directly leading to dysregulation of purine metabolism

**Fig. 3:**
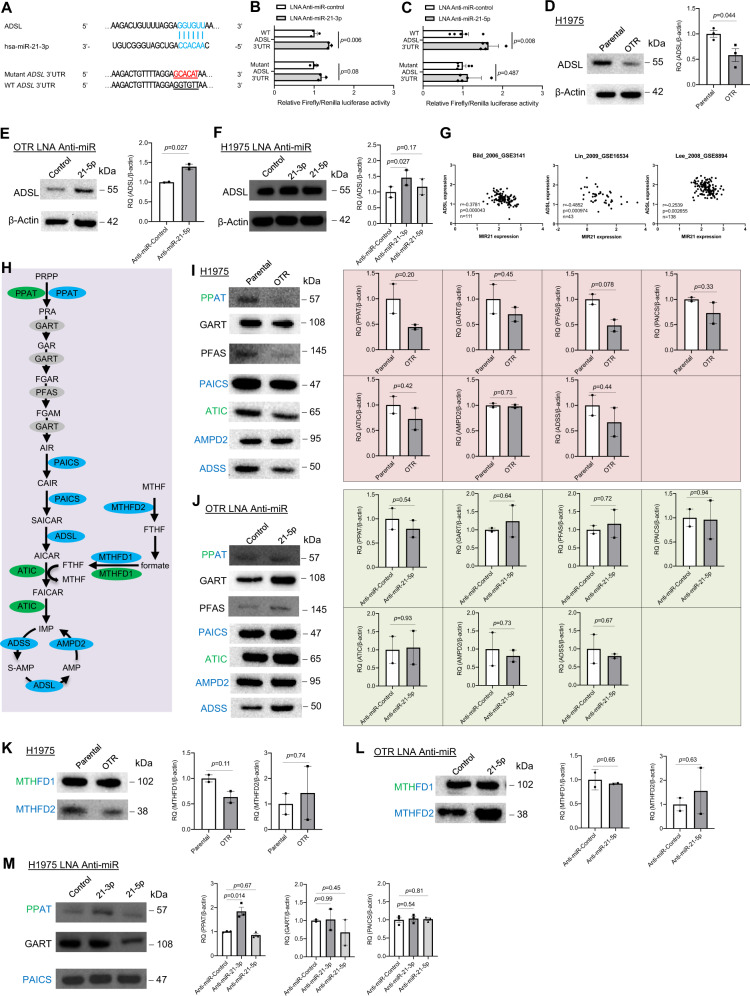
ADSL is the critical gene targeted by miR-21 in regulating purine metabolism. **A** Computational prediction of RNA duplex formation between miR-21-3p and ADSL mRNA’s 3’UTR (untranslated region). Mutations generated within the 3’UTR for the luciferase assay are shown in red. WT, wild-type. **B** Dual-luciferase reporter assay in H1975 cells treated with the miR-21-3p inhibitor. The Firefly luciferase and Renilla luciferase activities were measured 48 h post-co-transfection with the locked nucleic acid (LNA) miR-21-3p inhibitor (anti-miR-21-3p) or a control plasmid (anti-miR-control) and WT or mutant *ADSL* 3’UTR. *N* = 3 replicates. **C** Dual-luciferase reporter assay in H1975 cells treated with the miR-21-5p inhibitor. The Firefly luciferase and Renilla luciferase activities were measured 48 h post-co-transfection with the locked nucleic acid (LNA) miR-21-5p inhibitor (anti-miR-21-5p) or a control plasmid (anti-miR-control) and wild-type (WT) or mutant *ADSL* 3’UTR. *N* = 5 replicates. **D** Western blot analysis and quantification of ADSL in H1975 parental and osimertinib-tolerant (OTR) cells. β-Actin was used as a loading control. *N* = 3 replicates. **E** Western blot analysis and quantification of ADSL in H1975 OTR cells treated with the LNA miR-21-5p inhibitor (anti-miR-21-5p) or a control plasmid (anti-miR-control). β-Actin was used as a loading control. *N* = 2 replicates. **F** Western blot analysis and quantification of ADSL in parental H1975 cells treated with the LNA inhibitor against miR-21-3p (anti-miR-21-3p), miR-21-5p (anti-miR-21-5p), and a miRNA control (anti-miR-control). β-Actin was used as a loading control. *N* = 2 replicates. **G** A correlation between *MIR21* and *ADSL* gene expression in lung tumor tissues from patients. RNA expression data were extracted from three independent RNA transcriptomics datasets (GSE3141, GSE16534, and GSE8894) by the Lung Cancer Explorer web portal. Each dot represents one patient’s sample. **H** A diagram describing the purine de novo synthesis pathway and nucleotide cycle reactions. The enzymes in green and blue colors are predicted targets of miR-21-5p and miR-21-3p by the TargetScan analysis. The enzymes in gray color are non-predicted targets of either miR-21-5p or miR-21-3p. **I** Western blot analysis and quantification of PPAT, GART, PFAS, PAICS, ATIC, AMPD2, and ADSS in H1975 parental and OTR cells. β-Actin shown in (**D**) was used as the loading control. *N* = 2 replicates. **J** Western blot analysis and quantification of PPAT, GART, PFAS, PAICS, ATIC, AMPD2, and ADSS in H1975 OTR cells treated with the LNA miR-21-5p inhibitor (anti-miR-21-5p) or a control plasmid (anti-miR-control). β-Actin shown in (**E**) was used as the loading control. *N* = 2 replicates. **K** Western blot analysis and quantification of MTHFD1 and MTHFD2 in H1975 parental and OTR cells. β-Actin shown in (**D**) was used as the loading control. *N* = 2 replicates. **L** Western blot analysis and quantification of MTHFD1 and MTHFD2 in H1975 OTR cells treated with the LNA miR-21-5p inhibitor (anti-miR-21-5p) and a miRNA control plasmid (anti-miR-control). β-Actin shown in (**E**) was used as the loading control. *N* = 2 replicates. **M** Western blot analysis and quantification of PPAT, GART, and PAICS in H1975 parental cells treated with the LNA inhibitor against miR-21-3p (anti-miR-21-3p), miR-21-5p (anti-miR-21-5p), and a miRNA control (anti-miR-control). β-Actin shown in (**F**) was used as the loading control. *N* = 3 replicates. Data are mean ± s.e.m. and were analyzed with unpaired two-tailed *t-*test (**B**–**E**, **I**–**L**); Pearson correlation analysis (**G**); RM one-way ANOVA (**F**, **M**).

AICAR is the primary product of ADSL metabolism in de novo purine biogenesis. Thus, we inferred that ADSL repression leads to a reduced level of AICAR in the DTPCs. Our experimental evidence showing a negative correlation between AICAR levels and expression of miR-21-5p and miR-21-3p indicates that miR-21 might reduce AICAR levels via suppressing ADSL expression. We utilize complementary approaches including computational prediction and experimental validation to test the direct interaction between miR-21 and ADSL to validate this hypothesis. We tested if miR-21-5p or miR-21-3p could regulate ADSL directly. Computational prediction of miRNA downstream target using the TargetScan tool showed that *ADSL* mRNA has a sequence that could be bound directly by miR-21-3p (Fig. 3A).

Next, we performed dual transfection in H1975 cells using an oligonucleotide containing LNA miR-21-3p inhibitor and a wild-type (WT) ADSL 3’ UTR plasmid. The dual-glo firefly/renilla luciferase data demonstrated that blocking miR-21-3p increased WT ADSL 3’UTR activity 1.4-fold (*P* = 0.006) compared to the control group (Fig. 3B). To address if miR-21-5p binds to *ADSL* directly, we used a similar strategy of the dual-luciferase assay for 3’UTR activity of *ADSL* by blocking miR-21-5p in H1975 cells. Our data showed that inhibiting miR-21-5p with the synthetic LNA inhibitor against miR-21-5p increased ADSL 3’UTR activity by 1.7-fold (*P* = 0.008) (Fig. 3C). To further understand the binding specificity to the *ADSL* 3’UTR by miR-21, we constructed a mutant *ADSL* 3’UTR plasmid by changing a sequence of GGTGTT to GCACAT (Fig. 3A). As expected, inhibiting miR-21-3p did not significantly reduce the dual-glo luciferase activity of mutant ADSL 3’UTR (*P* = 0.08). Similarly, blocking miR-21-5p did not substantially decrease the dual-glo luciferase activity of mutant ADSL 3’UTR (*P* = 0.487). These data demonstrated that miR-21-3p directly regulates *ADSL*.

Furthermore, we anticipated that ADSL protein levels would be downregulated in the DTPCs due to increased levels of miR-21-3p. Western blot analysis showed that ADSL protein levels decreased in OTR cells compared to parental cells from H1975 (*P* = 0.044) (Fig. 3D and Supplementary Fig. 6A, B). Then we reduced the abundance of miR-21-5p and miR-21-3p using the LNA inhibitor against miR-21-5p in H1975 OTR cells. We found that blocking miR-21-5p effectively increased ADSL protein levels in OTR cells (*P* = 0.027) (Fig. 3E and Supplementary Fig. 6C, D). Blocking the passenger strand with the synthetic LNA inhibitor against miR-21-3p also increased ADSL protein expression in H1975 parental cells (*P* = 0.027) (Fig. 3F and Supplementary Fig. 6E, F). These data confirmed that miR-21-3p and miR-21-5p repress ADSL protein expression.

Using a bioinformatic tool, Lung Cancer Explorer [65], we performed a correlation analysis between expression for precursor *MIR21* and *ADSL* mRNA across 292 lung cancer patient tissues. The analysis showed that *MIR21* is negatively correlated to *ADSL* gene expression in three independent datasets (*r* = −0.38, *P* = 0.000043, *n* = 111 tumors, GSE3141 [66]; *r* = −0.49, *P* = 0.000974, *N* = 43 tumors, GSE16534 [67]; *r* = −0.25, *P* = 0.002655, *n* = 138 tumors, GSE8894 [68]) (Fig. 3G and Supplementary Tables 6–9). These data suggest that miR-21 is negatively correlated to ADSL.

These data validated our hypothesis on miR-21 as a direct repressor of *ADSL*. Several studies have proved that one miRNA may target multiple genes belonging to the same signaling pathway in lung cancer [69, 70]. Therefore, we asked if miR-21 could target other enzymes in the purine biosynthesis pathway besides ADSL. The purine de novo biogenesis pathway provides adenine and guanine building blocks for RNA and DNA synthesis [71]. The six metabolic enzymes including phosphoribosyl pyrophosphate amidotransferase (PPAT), phosphoribosylaminoimidazole synthetase (GART), phosphoribosylformylglycinamidine synthase (PFAS), phosphoribosylaminoimidazole carboxylase (PAICS), ADSL and aminoimidazole carboxamide formyltransferase (AICAR transformylase, ATIC) form the purinosome temporarily to catalyze phosphoribosyl pyrophosphate (PRPP) to IMP [72]. Complementary to the de novo purine synthesis pathway, the purine nucleotide cycle generates ammonia and fumarate from aspartate and IMP. These reactions are catalyzed by three enzymes, including adenosine monophosphate deaminase 2 (AMPD2), adenylosuccinate synthase (ADSS), and ADSL (Fig. 3H). Using the TargetScan tool, we found that other enzymes in the purine pathway are predicted targets of miR-21-5p and miR-21-3p. The anticipated miR-21-5p target genes include *PPAT* and *ATIC*. The predicted miR-21-3p target genes include *PPAT*, *PAICS*, *ADSL*, *ADSS*, and *AMPD2*. *GART* and *PFAS* were not listed as the predicted target genes of miR-21-5p and miR-21-3p. To investigate if any of these are confirmed targets of miR-21-5p and miR-21-3p, we looked at the Tarbase, a database of experimentally verified miRNA–gene interactions [73]. Only one report described an enhanced association of miR-21-3p with GART in human kidney 293S cells upon cellular stress [74]. Furthermore, our western blot analysis showed that these enzymes did not significantly decrease their expression in the OTR cells including PPAT (*P* = 0.20), GART (*P* = 0.45), PFAS (*P* = 0.078), PAICS (*P* = 0.33), ATIC (*P* = 0.42), AMPD2 (*P* = 0.73), and ADSS (*P* = 0.44) (Fig. 3I and Supplementary Fig. 7A–G). Blocking miR-21-5p with the LNA inhibitor against miR-21-5p did not significantly upregulate expression of these targets in H1975 OTR cells compared with cells treated with the control miRNA inhibitor (Fig. 3J and Supplementary Fig. 8A–G). These data suggest that miR-21-5p and miR-21-3p inhibit ADSL in purine metabolism in the DTPCs.

Due to one-carbon metabolism providing carbons for purine biogenesis [75], we asked if one-carbon metabolism was linked to the DTPCs driven by miR-21. ATIC uses 10-formyltetrahydrofolate (FTHF) for purine synthesis [76]. FTHF is converted from 5,10-methylenetetrahydrofolate (MTHF) by methylenetetrahydrofolate dehydrogenase (NADP + dependent) 2 (MTHFD2) in mitochondria and from formate by MTHFD1 in the cytosol [77]. By analyzing TargetScan, we found that *MTHFD1* is a predicted target gene of miR-21-3p and miR-21-5p and *MTHFD2* is a predicted target of miR-21-3p. The consumption of AICAR needs MTHF as a cofactor catalyzed by ATIC. We hypothesized that the increased MTHF contributed to reduced AICAR levels. To test this hypothesis, we measured MTHFD1 and MTHFD2 expression. Western blot data showed that expression of MTHFD1 and MTHFD2 did not significantly decrease in the OTR cells compared with the parental cells (Fig. 3K and Supplementary Fig. 9A, B). Blocking miR-21-5p with the miR-21-5p inhibitor did not significantly increase the expression of MTHFD1 and MTHFD2 (Fig. 3L and Supplementary Fig. 9C, D). This indicates that one-carbon metabolism might not be involved in miR-21-mediated purine metabolism. Blocking the passenger strand with the synthetic LNA inhibitor in the parental H1975 cells did not change GART (*P* = 0.99) and PAICS (*P* = 0.54) protein expression. Still, it increased PPAT expression significantly (*P* = 0.014) (Fig. 3M and Supplementary Fig. 10A–C). This suggests that miR-21-3p can regulate PPAT besides ADSL in purine metabolism. Inhibiting the guide strand with the LNA miR-21-5p inhibitor did not significantly change expression of PPAT (*P* = 0.67), GART (*P* = 0.45), and PAICS (*P* = 0.81). Thus, our data suggest that miR-21-5p and miR-21-3p regulate purine metabolism mainly through tunning ADSL.

### Blocking miR-21 increases reactive oxygen species and co-treatment with AICAR and osimertinib reduces antioxidant response

**Fig. 4:**
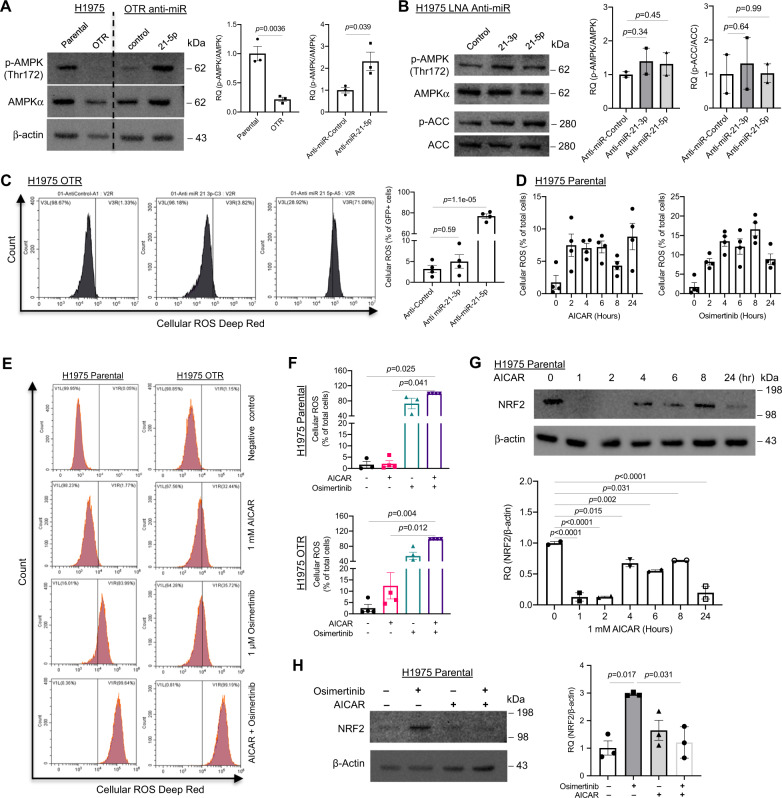
AICAR induces reactive oxygen species generation and prevents transition to drug tolerance. **A** Western blot analysis of p-AMPK and AMPK in parental and osimertinib-tolerant (OTR) cells from H1975 and OTR cells with the miR-21-5p inhibitor (anti-21-5p) and scrambled control (anti-miR-control). β-Actin was used as the loading control. The p-AMPK (Thr172) quantification was normalized to the total AMPK in comparing OTR versus parental cells and OTR anti-miR-control versus OTR anti-miR-21-5p cells. *N* = 3 replicates. **B** Western blot analysis and quantification for p-AMPK, AMPK, p-ACC, and ACC in H1975 cells treated with the LNA inhibitor against miR-21-3p (anti-miR-21-3p), miR-21-5p (anti-miR-21-5p), and a miRNA control (anti-miR-control). Total AMPK and total ACC were used as loading controls to quantify p-AMPK and p-ACC. *N* = 2 replicates. **C** Flow cytometry analysis for ROS levels in OTR cells with the LNA miR-21-3p inhibitor (anti-miR-21-3p), miR-21-5p inhibitor (anti-miR-21-5p), and a scrambled control (anti-miR-control) from H1975. The OTR cells were transfected with 120 nM LNA inhibitors labeled with fluorescein amidite (FAM), followed by flow cytometry analysis for ROS levels 8 h post-transfection. *N* = 4 replicates. **D** Timepoint ROS assay for H1975 parental cells treated with AICAR and osimertinib. The cells were treated with 1 mM AICAR (left) or 1 µM osimertinib (right) for 0, 2, 4, 6, 8, and 24 h followed by incubation with cellular ROS deep red dye. Then the deep red positive cells were analyzed by flow cytometry analysis. *N* = 4 replicates. **E** Representative images of flow cytometry analysis for ROS levels from H1975 parental and osimertinib-tolerant (OTR) cells. H1975 parental and OTR cells were treated with AICAR (1 mM), osimertinib (1 µM), AICAR (1 mM) combined with osimertinib (1 µM), and negative control for 4 h. ROS levels were measured via ROS deep red dye by flow cytometry. **F** Statistical analysis of ROS levels in H1975 parental and osimertinib-tolerant (OTR) cells treated with AICAR, osimertinib, AICAR combined with osimertinib, and negative control. *N* = 3–4 replicates. **G** Western blot analysis and quantification for NRF2 in H1975 cells treated with 1 mM AICAR for 0, 1, 2, 4, 6, 8, and 24 h. β-Actin was used as the loading control. *N* = 2 replicates. **H** Western blot analysis for NRF2 in H1975 parental cells treated with AICAR (1 mM), osimertinib (1 µM), AICAR (1 mM) combined with osimertinib (1 µM) and negative control for 4 h. β-Actin was used as the loading control. *N* = 3 replicates. Data are mean ± s.e.m. and were analyzed with unpaired two-tailed *t*-test (**A**); RM ANOVA (**B**); Brown–Forsythe and Welch ANOVA (**C**); Kruskal–Wallis ANOVA (**F**, **H**); one-way ANOVA (**G**).

We asked how a miR-21-ADSL-AICAR-purine signaling axis regulates drug tolerance to EGFR inhibitors. As an analog of AMP, AICAR activates AMP-activated protein kinase (AMPK) [78] leading to imbalanced ROS or redox status [79]. We asked if AMPK was inactivated in DTPCs compared with parental cells. As expected, western blot data demonstrated that phosphorylated AMPK (p-AMPK) was significantly downregulated in the OTR cells compared to parental cells from H1975 (*P* = 0.0036) (Fig. 4A and Supplementary Fig. 11A–C). And the reduced p-AMPK expression in the OTR cells was partially rescued by miR-21-5p knockdown (*P* = 0.039) (Fig. 4A and Supplementary Fig. 11A–C). In parental H1975 cells, expression of p-AMPK (*P* = 0.34 and *P* = 0.45, respectively) and phosphorylated acetyl-CoA carboxylase (p-ACC) (*P* = 0.64 and *P* = 0.99, respectively) was not changed significantly when treated with the LNA inhibitors against miR-21-3p and miR-21-5p (Fig. 4B and Supplementary Fig. 12A–D). These data indicated that miR-21 inhibition activates AMPK signaling pathway in the OTR cells.

AMPK senses and restores energy homeostasis and the redox balance [80]. We asked if miR-21 regulates ROS levels in our conditions. Our result showed that knocking down miR-21-5p dramatically increased ROS levels in the OTR cells more than the control and miR-21-3p inhibitors (*P* = 1.1e-05) (Fig. 4C), supporting previous evidence demonstrating targeting redox imbalance to overcome drug resistance [81]. This indicates that miR-21-5p plays an important role in keeping redox balance. To determine if AICAR mediates ROS generation, we measured cellular ROS levels in H1975 parental cells treated with 1 mM AICAR at different time points by flow cytometry analysis using a deep red ROS assay. ROS levels increased as early as 2 h after 1 mM AICAR treatment, and the increased ROS levels remained stable for 24 h (Fig. 4D and Supplementary Fig. 13A). Interestingly, 1 µM osimertinib treatment increased ROS generation gradually between 2 and 8 h post-induction. ROS levels fell at 24 h post-treatment with osimertinib (Fig. 4D and Supplementary Fig. 13B). The data support the idea that dysregulated AICAR-ROS signaling is relevant to the DTP state transition. We hypothesized that AICAR in combination with osimertinib could further increase ROS generation. We performed flow cytometry analysis to measure ROS levels 4 h after treatment with AICAR, osimertinib, and combo to address this question. Our data showed that a combination of AICAR and osimertinib dramatically increased ROS levels compared to either single treatment in both parental and OTR cells (Fig. 4E, F). Interestingly, H1975 OTR cells are more sensitive to AICAR treatment than H1975 parental cells, suggesting AICAR as a therapeutic molecule against the DTPCs (Fig. 4E, F).

NRF2 is an antioxidative protein that reduces oxidant levels [82]. Thus, inspired by the data showing increased ROS levels after AICAR treatment, we asked if AICAR treatment could induce antioxidant NRF2 expression. Our timepoint western blot data demonstrated that NRF2 protein expression decreased dramatically during the first 2-h treatment compared with untreated cells (*P* < 0.0001). NRF2 expression was kept lower within 24 h in cells after AICAR treatment than in untreated cells, suggesting reduced antioxidant capacity after AICAR treatment (Fig. 4G and Supplementary Fig. 14A, B). These reciprocal changes in ROS levels and NRF2 expression after AICAR treatment indicate that AICAR-induced imbalance of ROS and NRF2 leads to reduced antioxidant response.

A recent study showed that NRF2 regulated miRNA expression via binding to their promoters including the gene for pri-miR-21 (the transcript that generates miR-21-5p and miR-21-3p) [83]. Since oxidative stress induces NRF2 upregulation, we hypothesized that hydrogen peroxide (H_2_O_2_) but not AICAR treatment could upregulate miR-21-5p or miR-21-3p expression through activating NRF2. We performed a qRT-PCR analysis of miR-21-3p and miR-21-5p in cells treated with hydrogen peroxide (H_2_O_2_) for 4 h to test the hypothesis. Our data showed that increasing doses of H_2_O_2_ induced miR-21-3p and miR-21-5p upregulation in the OTR cells more than in parental cells (Supplementary Fig. 15A, B), indicating that the OTR cells adapt to the oxidative stress better than the parental cells. In contrast, the 4-h treatment with 0.1 mM and 1 mM AICAR did not significantly change miR-21-3p and miR-21-5p expression in parental and OTR cells from H1975 (Supplementary Fig. 15C, D). This indicates that AICAR might block miR-21-3p and miR-21-5p upregulation by targeting NRF2.

It was reported that reduced purine metabolism led to cancer cell differentiation [84] and differentiated cancer cells are more vulnerable to stress such as oxidant stress [85]. We hypothesized that AICAR treatment decreases antioxidant response in tumor cells treated with EGFR TKIs. Our western blot data on NRF2 expression demonstrated an increase in H1975 cells after osimertinib treatment (*P* = 0.017), indicating an increased antioxidant response in tumor cells induced by osimertinib treatment (Fig. 4H and Supplementary Fig. 16A, B). This increase in NRF2 expression was reduced dramatically (*P* = 0.031) when AICAR was co-administrated with osimertinib (Fig. 4H and Supplementary Fig. 16A, B). These data indicated that AICAR co-treatment with osimertinib could prevent antioxidant response in tumor cells.

### Knocking out *MIR21* induces the concurrent increase of purine metabolism and decreased glutathione metabolism

**Fig. 5:**
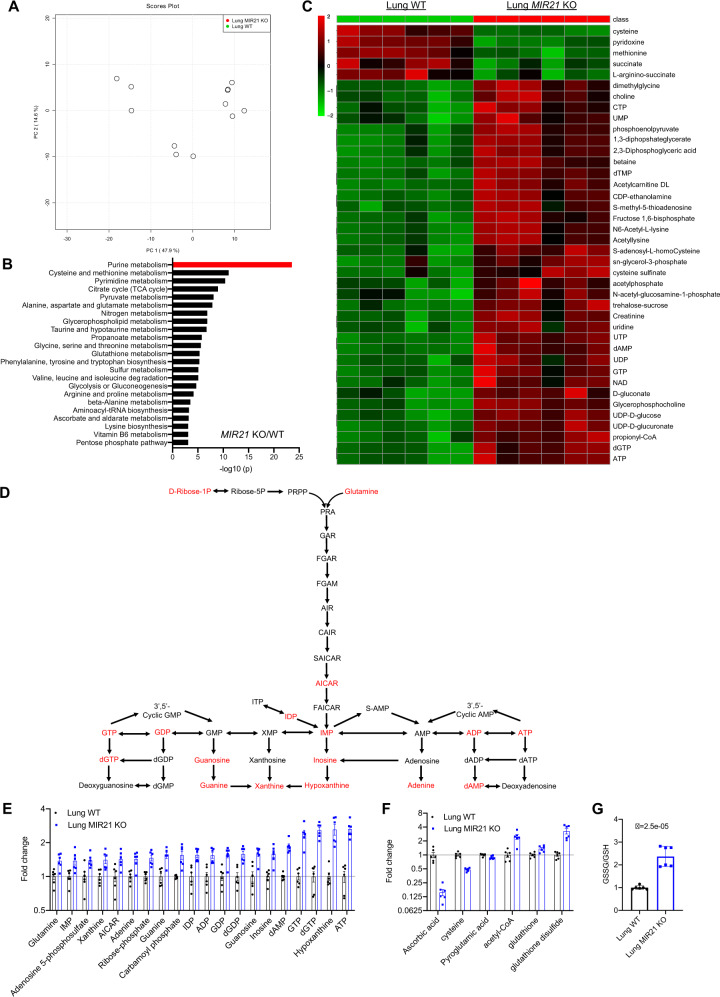
Knocking out *MIR21* promotes purine metabolism and decreases glutathione metabolism. **A** Principal component scores (PCA) of metabolites in whole lung tissues from wild-type (WT) and *MIR21* knockout (KO) mice. *N* = 6 replicates. **B** Metabolic pathway analysis of lung tissues from *MIR21* KO and WT mice. *N* = 6 replicates. **C** Top 40 metabolic hits differentially expressed in the lung tissues from *MIR21* WT and KO mice. *N* = 6 replicates. **D** A diagram of purine metabolism. Red font indicates upregulated metabolites in comparing *MIR21* KO and WT mouse lung tissues. **E** Upregulated metabolic hits in purine metabolism in lung tissues from the *MIR21* KO mice compared with WT mice. *N* = 6 replicates. **F** Downregulated and upregulated metabolic hits in the glutathione metabolism pathway in lung tissues from *MIR21* KO mice compared with WT mice. *N* = 6 replicates. **G** The ratio of glutathione disulfide (GSSG)/glutathione (GSH) in lung tissues from *MIR21* KO mice compared with WT mice. *N* = 6 replicates. Data are mean ± s.e.m. and were analyzed with an unpaired two-tailed *t*-test (**A**, **B**, **C**, **G**).

Since miR-21 regulates purine metabolism in our cultured lung cells in vitro, we tested which metabolic pathways could be modulated by miR-21 in physiological conditions. Utilizing a unique *MIR21* knockout (KO) mouse model [86], we did not find any noticeable phenotypic changes in the *MIR21* KO mice compared with wild-type littermates when grown under standard laboratory conditions for the length of the study. We collected whole lung tissues from 8-week-old animals (males and females) and performed metabolomics profiling (Fig. 5A). Metabolic pathway analysis showed that the top significantly changed pathways were purine metabolism, cysteine and methionine metabolism, pyrimidine metabolism, citrate cycle (TCA cycle), and pyruvate metabolism (Fig. 5B). Consistent with our in vitro data, this in vivo data further demonstrated that dysfunction of purine metabolism was the top metabolic change in lung tissues when the *MIR21* gene was lost. Among differentially expressed metabolites (Supplementary Table 10), the top downregulated metabolites were cysteine, pyridoxine, methionine, and succinate (Fig. 5C). And top upregulated metabolites were ATP, dGTP, propionyl-CoA, and UDP-D-glucuronate (Fig. 5C). In the top dysregulated pathway – purine metabolism, many metabolites were upregulated after *MIR21* was knocked out (Fig. 5D). The AICAR level was upregulated 1.5-fold in *MIR21* KO lung tissues compared with WT lung tissues (Fig. 5E). Other upregulated purine metabolites included IDP, IMP, inosine, hypoxanthine, xanthine, guanine, guanosine, GDP, GTP, dGTP, adenine, dAMP, ADP, and ATP. The levels of ribose-phosphate and glutamine that provide ribose and nitrogen for the synthesis of purine were also upregulated 1.5-fold and 1.4-fold, respectively (Fig. 5E).

Our previous data showed that AICAR-induced ROS increased in vitro. ROS is eliminated by endogenous and exogenous antioxidant metabolites such as glutathione and ascorbic acid [87]. We tested if knocking out *MIR21* could regulate glutathione metabolism. Our data demonstrated that metabolites including ascorbic acid, cysteine, and pyroglutamic acid were downregulated in *MIR21* KO lung tissues (Fig. 5F). Further analysis showed that the ratio of glutathione disulfide (GSSG)/glutathione (GSH) increased 2.4-fold when *MIR21* was deleted in the lung tissues (*P* = 2.5E-05) (Fig. 5G). These indicate that *MIR21* KO decreased ROS scavenging capacity in the mouse tissues. Collectively, our in vivo data confirmed *MIR21’s* role in regulating purine metabolism and glutathione metabolism.

## Discussion

The purine de novo biogenesis and degradation pathway provides adenine and guanine building blocks for RNA and DNA synthesis [71]. The six metabolic enzymes, including PPAT, GART, PFAS, PAICS, ADSL, and ATIC, form a purinosome temporarily to catalyze PRPP to IMP [72]. In the presence of EGFR-TKI osimertinib, some drug-sensitive parental cells transit to the DTP state resulting in the survival of cells in the stress [88]. Our metabolomics analysis of the DTPCs showed that purine metabolism is the top signaling pathway demonstrating an increase of a panel of metabolites in purine metabolism except for AICAR. Supported by previous discoveries showing that increasing amounts of purine metabolites promote cancer cells’ proliferation [57, 89], our data suggest that increasing purine degradation products might promote survival of the DTPCs under osimertinib-induced stress. Concurrent to the increased purine degradation blocks in the DTPCs, other metabolites providing nitrogen atoms of the purine ring such as glutamine levels also increased in our metabolomics data (Supplementary Table 3). These data support a comprehensive increase of purines in the DTPCs. Strikingly, as an intermediate metabolite in purine de novo biosynthesis, AICAR is one of the most downregulated metabolites in the DTPCs compared with parental cells. AICAR is converted from SAICAR by ADSL and is catalyzed into FAICAR by ATIC. A reduction of AICAR indicates ADSL is silenced or ATIC is upregulated. Our western blot assay demonstrated more decreases in ADSL than ATIC in the OTR cells, indicating that these changes might cause less supply of AICAR, resulting in reduced AICAR levels in the DTPCs. Another proof supporting silenced ADSL is an increase of S-AMP in the DTPCs because S-AMP is converted to AMP by ADSL in the purine nucleotide cycle. Thus, our data first demonstrate silenced ADSL and reduced AICAR in the DTPCs in lung cancer. Clinically most ADSL-deficient children display marked psychomotor delay and accumulation of SAICAR but reduced AICAR [90]. Our innovative discovery of inactivated ADSL-AICAR signaling in the DTPCs may direct a clinical study in lung cancer patients who receive EGFR TKIs and then undergo the DTP state transition in the future.

Our study first links reduced AICAR and ROS to the acquisition of the DTP state in lung cancer cells. Supporting our findings, it has been recently reported that a signature in DTPCs has lower ROS levels and higher NRF2 expression after drug treatment than in untreated cells in the lung and other cancers [91]. Our study rescued this reduced level of ROS by treating parental cells with AICAR resulting in re-sensitizing DTPCs to osimertinib in H1975 cells. Increasing AICAR leading to reduced antioxidant NRF2 expression indicates that the lung tumor cells might control ROS at low levels to avoid oxidative damage. This mechanism was supported by evidence of reduced glutathione levels in these DTPCs from our data. Further proof of decreased expression of NRF2 protein after combination treatment of AICAR and osimertinib provides a possible mechanism for the parental cells being more vulnerable to AICAR-induced ROS. Thus, our data confirm the effects of AICAR in preventing the acquisition of the DTP state by regulating ROS levels in lung cancer cells. A previous study demonstrated reduced ROS levels in some breast cancer stem cells compared with noncancer stem cells [85]. Cancer stem cells have been described to contribute to drug resistance in many cancers via an intrinsic mechanism [92, 93]. Although the DTP state is acquired in the DTPCs, our previous study has shown partially overlapping genes between DTPCs and cancer stem cells [14]. The DTPCs may acquire some CSC features leading to less responsiveness to treatment by a de-differentiation mechanism. Stimulation of purine biosynthesis promotes stemness in some cancers [84]. Here AICAR inhibits purine metabolism by blocking the enzyme ADSL. Thus, it is reasonable to speculate that AICAR might promote cancer differentiation, allowing the differentiated cancer cells are more sensitive to osimertinib. It will be interesting to understand how DTPCs acquire features of cancer stem cells through AICAR-mediated purine metabolism in the future.

Consistent with the data in previous studies in drug-resistant lung cancer [47, 94], we also found an increase of miR-21-5p expression in the DTPCs compared to parental cells. The left guide miRNA strand (5p, left arm) is bound to another right passenger strand [3p] composing a miRNA duplex in the cytoplasm. Few studies look at the passenger strand of miR-21 due to a conventional concept regards this passenger strand being degraded after separating from the guide strand [95]. Our data challenged the notion that miR-21-3p expression is abundant in acquiring the DTP state in cancer cells. This suggests that degradation of miR-21-3p might be delayed or interrupted in the DTPCs compared to parental sensitive cells. The concurrent dysregulation of miR-21-5p and miR-21-3p in the acquisition of the DTP state and post-transfection of miR-21-5p with the LNA inhibitor suggest that miR-21-5p might increase miR-21-3p stability in the context of drug tolerance. Supporting this hypothesis, the treatment with hydrogen peroxide upregulated the expression of both strands in both parental and DTPCs from H1975. Consistent with our discovery, other studies found that guide and passenger arms become stable by binding to argonaute 1 and argonaute 2, respectively [96, 97]. Another possible mechanism might be relevant to miRNA arm switching due to a modification of the passenger strand that causes alternative processing by Dicer 1, ribonuclease III (DICER1), and selection of the passenger strand [98]. It will be interesting to explore mechanisms of strand selection impacted by post-transcriptional modifications on miRNAs and their binding to argonaute.

Our study has demonstrated that both strands of miR-21 are abundant in expression and activity in regulating ADSL in the purine metabolism pathway. We found that miR-21-3p directly represses ADSL expression. Strikingly, miR-21-5p also regulates ADSL expression in a similar way to miR-21-3p. This role of miR-21-5p might be an indirect regulation of ADSL via miR-21-3p. In addition to targeting ADSL, miR-21-3p represses apoptotic factors, including PTEN and PDCD4 in PC9ER and H1975 cells. Collectively, our data indicate that the miR-21 family members coordinate with each other to regulate the acquisition of the DTP state by targeting the apoptosis and purine metabolism pathway. It will be interesting to expand the miR-21-mediated network using new approaches such as in silico models [99].

This is the first report that miR-21-3p is enhanced in the DTPCs in lung cancer together with miR-21-5p. miR-21-5p and miR-21-3p increase cells’ drug tolerance by regulating the purine metabolism and oxidative stress pathway. Our data showing synergy between AICAR and osimertinib provides a promising direction for targeting purine metabolism and oxidative stress in preventing drug tolerance against anti-EGFR therapy in the future.

## Methods

### Cell culture and cell lines

Each cell line was maintained in a 5% CO_2_ atmosphere at 37 °C. Human lung *EGFR*-mutant cell lines H1650, H1975, HCC827, PC9, and H3255 (provided by Dr. Susumu Kobayashi) were cultured in DMEM (high glucose) (GIBCO) with 10% FBS, 2 mM l-glutamine and 1% penicillin–streptomycin. Immortalized tracheobronchial epithelial AALE cells (provided by William C. Hahn) were derived as previously described [100] and maintained in SAGM media (Lonza). Cell lines were negative for mycoplasma using the MycoAler Kit (Lonza).

### 3D cultures

Single-cell suspensions (2000 cells/well/20 µl) were co-plated with geltrex (25 µl) in 96-well non-treated clear plates (Corning, Cat# 08-772-53). The plate was incubated for 20 minutes at 37 °C, and 100 µl of complete growth media were added. The complete growth media was advanced DMEM/F12 with glutamax [1×], HEPES [1×], 1.25 mM N-acetylcysteine, 10 mM nicotinamide, 10 µM Forskolin, B27 [1×], 5 ng/ml Noggin, 100 ng/ml FGF10, 20 ng/ml FGF2, 50 ng/ml EGF, 10 ng/ml PDGFA, 10 ng/ml FGF7, and 1% penicillin–streptomycin as mentioned previously [14]. The media was changed every 3 days in 24 days. The phase-contrast 3D cultures were photographed from three random fields per group under a microscope (Evos FL, Life Technology).

### *MIR21* knockout mouse model

Both wild-type (strain# B6129SF2/J and stock# 101045) and *MIR21* knockout mice (strain# B6;129S6-*Mir21a*^*tm1Yoli*^/J Homozygote or *MIR21* null and stock# 016856) [86] in males and females were purchased from the Jackson Laboratory. The animal protocol was approved by Beth Israel Deaconess Medical Center Biological Resource Center Institutional Animal Care and Use Committee (IACUC). Both males and females at 8 weeks of age were randomly used for isolating lung tissues for metabolomics study. The whole lung tissues were isolated from mice immediately after euthanasia. After a quick rinse with cold PBS, the lung tissues were chopped into small pieces, followed by fast freezing by liquid nitrogen. Six mice-derived lung tissues from each group proceeded to the metabolomics study. Investigators were blinded to the group allocation during the procedure and metabolomics analysis.

### Antibodies and reagents

For western blotting, primary anti-p-AMPK (Thr172, clone 40H9) (1:500, Cat# 2535s), anti-AMPK (1:1000, Cat# 2532s), anti-p-ACC (Ser79, 1:500, Cat# 3661s), anti-ACC (1:1000, Cat# 3662s), anti-PFAS (1:1000, Cat# 61852s) anti-PTEN (1:1000, Cat# 9552S), and anti-PDCD4 (1:1000, Cat# 9535s) antibodies were from Cell Signaling Technology. Primary anti-ATIC (1:1000, clone H-3, Cat# sc-365402), anti-GART (1:1000, clone F-8, Cat# 166447), anti-MTHFD1/1 L (1:1000, clone D-9, Cat# sc-376722), anti-MTHFD2 (1:1000, clone A-2, Cat# sc-390708), anti-AMPD2 (1:1000, clone QQ13, Cat# sc-100504), and anti-NRF2 (1:1000, clone A-10, Cat# sc-365949) antibodies were from Santa Cruz Biotechnology. Primary anti-PPAT (1:1000, Cat# Ab125864) and anti-ADSS/ADSS2 (1:1000, clone EPR12331-52, Cat# Ab174842) antibodies were from Abcam. Primary anti-PAICS (1:1000, clone 2C9, Cat# GTX83950) was from GeneTex. Primary anti-ADSL (1:500, Cat# HPA000525) was from Sigma-Aldrich. Mouse anti-β-actin (1:10,000, clone C4, Santa Cruz, Cat# sc-47778) was used as a loading control. Secondary goat anti-rabbit (1:1000, Cat# 32460) and goat anti-mouse (1:1000, Cat# 32430) antibodies were purchased from Thermo Fisher Scientific.

### Small molecules

Osimertinib (Cat# S7297), WZ4002 (Cat# S1173), and gefitinib (Cat# S1025) were purchased from Selleck Chemicals. AICAR (Cat# 2840) was purchased from Tocris. H_2_O_2_ (Cat# H1009-100ML) was purchased from Sigma-Aldrich.

### Small molecule treatment

AICAR, osimertinib, WZ4002, and gefitinib were reconstituted in sterile dimethyl sulfoxide (DMSO) at a stock concentration of 200 mM, 10 mM, 10 mM, and 20 mM, respectively. H_2_O_2_ was diluted in growth media (DMEM supplemented with 10% FBS and 1% penicillin–streptomycin) to achieve 0, 0.5, and 1 mM concentrations. To achieve the required concentration, the compound of interest was serially diluted in growth media for mono treatment. For combination treatment, AICAR and osimertinib were serially diluted in growth media to achieve 2x of the necessary concentration and were combined to get the 1× working concentration.

To establish osimertinib-tolerant (OTR) cells, the parental cells were treated with 20 nM osimertinib or a vehicle for 14 days from HCC827 and PC9. To establish osimertinib-tolerant cells, the parental H1975 were continuously treated with 100 nM osimertinib for 2 weeks. To establish WZ4002-tolerant DTPCs in PC9, the parental PC9 were treated by culturing in 300 nM WZ4002 for 2 weeks.

For cells with a short-term response to WZ4002, the parental PC9 cells were treated with 1 µM WZ4002 or a vehicle for 24 h. The WZ4002-tolerant cells were taken out of the drug for 48 h prior to being rechallenged with WZ4002 or a vehicle for 48 h.

### Cell viability assay

Cells (3 × 10^3^/well) were seeded in 96-well plates (Falcon) in three replicates. After 6 h of plating, the spent medium was replaced with a fresh medium containing vehicle or serially diluted solutions of AICAR or osimertinib. At 3 days post-drug treatment, 50 μl of CellTiter-Glo (Promega, Cat# G7570) was added to each well. The samples were incubated for 10 min in the dark before detection with the EnVision plate reader (Perkin-Elmer). For combination treatment, cells (3 × 10^3^/well) were seeded in four 96-well plates, each representing an independent replicate. After 6 h of plating, the spent media was replaced with a medium containing AICAR and osimertinib at the indicated concentrations. The cell viability was measured by CellTiter-Glo luminescent assay.

### Western blots

H1975-derived parental, OTR, OTR with a scrambled control and miR-21 inhibitors were treated in different groups at concentrations of 0.1 µM of osimertinib and 1 mM of AICAR for 24 h. For measuring protein changes at various time points, H1975 cells were treated with 1 mM AICAR for 0–24 h. Cells were then lysed with 300 µL RIPA buffer (Thermo Fischer Scientific) with a cocktail of inhibitors against protease and phosphatase (Thermo Fischer Scientific). The collected cells were centrifuged for 40 min at 15,000 rpm at 4 °C, followed by a collection of the supernatant. Protein concentration was measured by Pierce BCA Protein Assay (Thermo Scientific). SDS-Page was performed using standard protocol for NuPAGE Bis-Tris Mini Gels by Thermo Fisher Scientific. Samples were combined with NuPAGE LDS Sample Buffer [4×], NuPAGE Reducing Agent [10×], and DI water. 200 mL of 1× NuPAGE MOPs Running Buffer was added to 500 µL NuPAGE antioxidant for upper chamber buffer. SDS-PAGE was run using PowerEase for 45 min on a 200 V constant. Membrane transfer was performed overnight at 17 V. Blocking was performed with 5% milk in phosphate buffer saline in Tween 20 (PBS-T) for 1 h at room temperature, followed by overnight primary antibody incubation. Secondary antibody incubation was performed for 1 h at room temperature. The membrane was washed with PBS-T between incubations. The protein was detected using SuperSignal West PICO Plus Chemiluminescent substrate (Thermo Fisher Scientific) and was visualized by ChemiDoc Imaging Systems (BioRad Laboratories). The protein band intensity was quantified by Image Lab (v6.0.1, BioRad Laboratories).

### RNA extraction and qRT-PCR

Total RNA was isolated from lung immortalized or malignant cell lines without treatment (AALE, PC9, H1650, H3255, HCC827, H1975), H1975-derived parental and OTR cells with or without AICAR treatment (0, 0.1, and 1 mM), or H_2_O_2_ treatment (0, 0.5 and 1 mM) for 4 h using mirVana miRNA Isolation Kit (Ambion #AM1561, Invitrogen). Similarly, the lentiviral or LNA miRNA inhibitor-treated cells were processed for RNA extraction. For miRNA expression analysis, 10 ng RNA in each sample was input for consecutive reactions, including Poly(A) Tail reaction, Ligation reaction, Reverse Transcription reaction, and miR-Amp reaction using the TaqMan Advanced miRNA cDNA synthesis kit (Applied Biosystems #A28007). miRNA expression levels were determined by TaqMan Fast Advanced miRNA Assays (Applied Biosystems) protocol. For pre-miRNA expression analysis, 500 ng RNA in each sample was input using a high-capacity RNA-to-cDNA kit according to the manufacturer’s instructions (Applied Biosystems #4387406). Real-time PCR was performed using TaqMan probes on QuantStudio Real-Time PCR (Thermo Fisher Scientific). TaqMan Advanced miRNA probes (Applied Biosystem) included the following miRNAs: hsa-miR-21-5p (Cat#477975_mir), hsa-miR-21-3p (Cat#479773_mir), hsa-miR-423-5p (Cat#478090_mir), and hsa-miR-186-5p (Cat# 477940_mir). Taqman gene expression probes included hsa-pre-miR-21 (Cat# hs03302625_pri). Hsa-miR-423-5p or hsa-miR-186-5p and *GAPDH* were used as endogenous controls to analyze miRNA and gene expression.

### miRNA-seq

For microRNA sequencing (miRNA-seq) of OTR cells, the paired parental and OTR cells (treated with 20 nM osimertinib or a vehicle for 14 days) from HCC827 and PC9 cells were applied. For miRNA-seq of WZ4002-tolerant cells from an independent lab [45], the WZ4002-tolerant cells (established by culturing in 300 nM WZ4002 for 2 weeks) from PC9 were processed. For miRNA-seq of cells with a short-term response to WZ4002, the parental PC9 cells treated with 1 µM WZ4002 (WZ4002_PC9) or a vehicle for 24 h were used. The WZ4002-tolerant cells were taken out of the drug for 48 h prior to being rechallenged with WZ4002 (WZ4002_PC9_WP2) or a vehicle (vehicle_PC9_WP2) for 48 h. The total RNA samples (1 µg) were applied by LC Sciences for miRNA-seq. All RNA samples were analyzed for quality on an Agilent 2100 Bioanalyzer. The RNA samples were processed utilizing Illumina’s TruSeq small RNA sample preparation protocol for small RNA library generation (Part# 15004197 Rev. F, Cat# RS-200-9002DOC). The subsequent sequencing was performed on the HiSeq 2500 platform for 1 ×50-nt single-end sequencing, and the sequencing adaptor was trimmed from the raw reads. The reads were then mapped to the miRBase v21 (http://www.mirbase.org/) and the human genome (GRCh37) using Bowtie [101]. The mapping results were summarized using an in-house script to estimate the number of reads mapped to each miRNA. Normalization was done using the median of the ratio of the read count to the geometric mean of read counts across samples as implemented in DESeq [102].

### Transfection by LNAs in vitro

Tumor cells were plated in a complete growth medium in a six-well plate to reach 50–60% confluence. In all, 120 nM of fluorescein-labeled LNA anti-miR-21-3p (Sequence: AGCCCATCGACTGGTGTT) (Cat# 339121 YI04101754-ADB, Exiqon-Qiagen), LNA anti-miR-21-5p (sequence: CAACATCAGTCTGATAAGCT) (Cat#4100689-011, Exiqon-Qiagen) or a negative control (sequence: TAACACGTCTATACGCCCA) (Cat#199006-011, Exiqon-Qiagen) with PureFection (System Biosciences) were applied for transfection. The transfected cells were harvested after culturing for 8 h (for ROS detection by flow cytometry) or 48 h (for western blot assay).

### Transient transfection and dual-glo luciferase assay

PureFection (System Biosciences) was used for transient transfection. In total, 100 ng of wild-type (Genocopoeia, HmiT117862-MT06) or mutant 3’UTR reporter construct of *ADSL* (Genocopoeia, HmiT117862-MT06-01) was co-transfected into H1975 cells with 120 nM of LNA anti-miR-21-3p, LNA anti-miR-21-5p, or a negative control (Exiqon-Qiagen) in three~five replicates. Firefly and Renilla luciferase activities were measured 48 h post-co-transfection using Dual-Glo Luciferase Assay (Promega, E2940). The firefly luminescence was normalized to renilla luminescence as an internal control for transfection efficiency. miR-21-3p-binding site GGTGTT was substituted with GCACAT in mutated *ADSL*.

### Lentiviral infection

For lentiviral overexpression (Lenti miRa-GFP-hsa-miR-21-5p, Cat# mh15276) or knockdown of miR-21-5p (Lenti miRa-Off-hsa-miR-21-5p virus, Cat# mh35326), cells (HCC827 and H1975) at 70% confluence were transduced with the lentiviral particles labeled with green fluorescence protein (GFP) (Applied Biological Material Inc, ABM) for 48 h in the presence of 1:100 Viralplus transduction enhancer (ABM) and 8 μg ml^−1^ polybrene (Sigma). The Lenti III-miR control virus (Cat# M002) and the Lenti III-miR-Off control virus (Cat# M008) serve as negative controls for miRNA overexpression and knockdown, respectively. Two days after infection, puromycin was added to the media at 0.5 μg ml^−1^, and cell populations were selected for 1–2 weeks.

### Metabolite extraction

The metabolomics samples from paired OTR and parental cells, OTR cells with miR-21 knockdown and scrambled control from H1975, and *MIR21* knockout and wild-type mice were prepared according to a previous method [103]. Briefly, the cells and the tissues were incubated with 80% methanol at −80 °C for 15 min. The cell and tissue lysate/methanol mixture was centrifuged at 4500 × *g* at 4 °C for 15 min three times in a cold room. The supernatants were dried entirely by speedVac and were further processed for liquid chromatography-mass spectrometry (LC-MS) analysis. Five to six biological replicates were used in each group and the analysis was normalized with the same number of cells or the same amount of tissues in each group.

### Targeted mass spectrometry

Cell samples were resuspended in high-performance liquid chromatography (HPLC) grade water for mass spectrometry as described previously [14]. Briefly, the solutions were injected and analyzed using a hybrid 5500 QTRAP triple quadrupole mass spectrometer (AB/SCIEX) coupled to a Prominence UFLC HPLC system (Shimadzu) via selected reaction monitoring (SRM) of a total of 274 unique endogenous water-soluble metabolites for steady-state analyses of samples. Some metabolites were targeted in both positive and negative ion mode for a total of 306 SRM transitions using positive/negative ion polarity switching. Peak areas from the total ion current for each metabolite SRM transition were integrated using MultiQuant v2.1 software (AB/SCIEX). Comprehensive metabolomic data analysis was performed using MetaboAnalyst 4.0 [104].

### ROS assay by flow cytometry

For comparisons of ROS levels between H1975 parental and OTR cells, as well as OTR cells treated with a scrambled control, miRNA inhibitors labeled with fluorescein against miR-21-5p and miR-21-3p 8 h post-transfection, the cells above (1 × 10^4^ cells/250 μl/well) were seeded in a 24-well plate (Falcon). After 24 h, cells were treated for 4 h with 250 μl of vehicle, AICAR (1 mM), osimertinib (1 μM), or a combo of AICAR (1 mM) and osimertinib (1 μM) and then were trypsinized and collected. For longitudinal measurement of ROS levels, H1975 parental cells (12 × 10^4^ cells/250 μl/well) were seeded in a 24-well plate (Falcon). After 24 h, cells were treated with 250 μl of AICAR (1 mM) and osimertinib (1 μM) for 0, 2, 4, 6, 8, and 24 h before trypsinization and collection. Reactive oxygen species (superoxide and hydroxyl radical) were measured with deep red fluorescence (Abcam, Cat# ab186029). The single-cell suspension was incubated for 45 min at 37 °C with deep red dye diluted to 1× in assay buffer for ROS detection. After incubation, the dye was removed, and cells were washed once with PBS and resuspended in phenol red-free DMEM (Gibco, Cat# 2187289) supplemented with 10% FBS and 1% penicillin–streptomycin. The fluorescence was measured with a flow cytometer using CytoFLEX (Beckman Coulter) via APC channel (Ex/Em:633/660). In all, 10,000 independent events were analyzed with CytExpert software (Beckman Coulter) from the total live cells (OTR and parental) and the total GFP^+^ cells (OTR treated with the LNA inhibitors labeled with fluorescein).

### Statistical analyses

All experiments were performed in two to five biological replicates and independently reproduced as indicated in figure legends. Investigators were blinded to the group allocation during the procedure and data analysis. Data are presented as the means ± SEM. Unless otherwise stated, statistical significance was determined by a Student’s two-tailed *t*-test by GraphPad Prism (v8.4.3). *P* < 0.05 was considered statistically significant. Two-tailed *t*-test with Welch’s correction was applied for two samples with unequal variances. For three and more normally distributed samples with equal variances, one-way ANOVA was used for multiple comparisons. For three and more normally distributed samples with unequal variances, Brown–Forsythe and Welch ANOVA was used for multiple comparisons. For three and more samples that are not normally distributed, the Kruskal–Wallis test was used for multiple comparisons. For three and more matched samples, RM one-way ANOVA was used for multiple comparisons. Pearson correlation coefficient was used for correlation analysis between ADSL and miRNA-21 expression in lung tumor tissues from patients from three independent datasets in the Lung Cancer Explorer web portal. Significance for Bliss synergy analysis was calculated via a one-sample *t*-test.

## Supplementary information

Fig S1

Fig S2

Fig S3

Fig S4

Fig S5

Fig S6

Fig S7

Fig S8

Fig S9

Fig S10

Fig S11

Fig S12

Fig S13

Fig S14

Fig S15

Fig S16

Supplemental texts and figures

Related Manuscript File-original data files

## Data Availability

Data from this study have been deposited in the Gene Expression Omnibus (GEO) databases under the following accession: GSE103352 (miRNA-seq). The results shown in this manuscript were partially based upon data generated by the Lung Cancer Explorer portal: https://lce.biohpc.swmed.edu/lungcancer/. The experimentally validated miRNA–gene interactions were collected from the Tarbase (v8): https://carolina.imis.athena-innovation.gr/diana_tools/web/index.php?r=tarbasev8%2Findex. The genetic mutation status was confirmed by the Cansar portal (v3.0 beta) (https://cansar.icr.ac.uk/) and cancer Catalogue Of Somatic Mutations In Cancer (COSMIC) (http://cancer.sanger.ac.uk/cosmic/sample/overview?id=722040). The small molecule combinational treatment data were analyzed with Combenefit2.0 (Cancer Research UK Cambridge Institute) using the Bliss independence model. The data that support the plots within this paper and other findings of this study are available from the corresponding author upon reasonable request.
