# Correction to: MicroRNA-21 guide and passenger strand regulation of adenylosuccinate lyase-mediated purine metabolism promotes transition to an EGFR-TKI-tolerant persister state

**DOI:** 10.1038/s41417-022-00523-9

**Published:** 2022-08-25

**Authors:** Wen Cai Zhang, Nicholas Skiados, Fareesa Aftab, Cerena Moreno, Luis Silva, Paul Joshua Anthony Corbilla, John M. Asara, Aaron N. Hata, Frank J. Slack

**Affiliations:** 1 Harvard Medical School Initiative for RNA Medicine, Department of Pathology, Cancer Center, Beth Israel Deaconess Medical Center, Harvard Medical School, Boston, MA 02215 USA; 2 Department of Cancer Division, Burnett School of Biomedical Sciences, College of Medicine, University of Central Florida, 6900 Lake Nona Blvd, Orlando, FL 32827 USA; 3 Department of Medicine, Division of Signal Transduction, Beth Israel Deaconess Medical Center, Harvard Medical School, Boston, MA 02215 USA; 4 Department of Medicine, Massachusetts General Hospital, Charlestown, MA 02129 USA

**Keywords:** Cancer genetics, Cancer genetics

Correction to: *Cancer Gene Therapy* 10.1038/s41417-022-00504-y, published online 15 July 2022

In this article Fareesa Aftab was incorrectly denoted as the corresponding author.

The original article has been corrected.

