# Supplementary figures and images for: MicroRNA-21 guide and passenger strand regulation of adenylosuccinate lyase-mediated purine metabolism promotes transition to an EGFR-TKI-tolerant persister state
Source: Cancer Gene Ther. 2022 Jul 15;29(12):1878–94. doi: 10.1038/s41417-022-00504-y (PMC9750876; doi:10.1038/s41417-022-00504-y)

## Slide 1
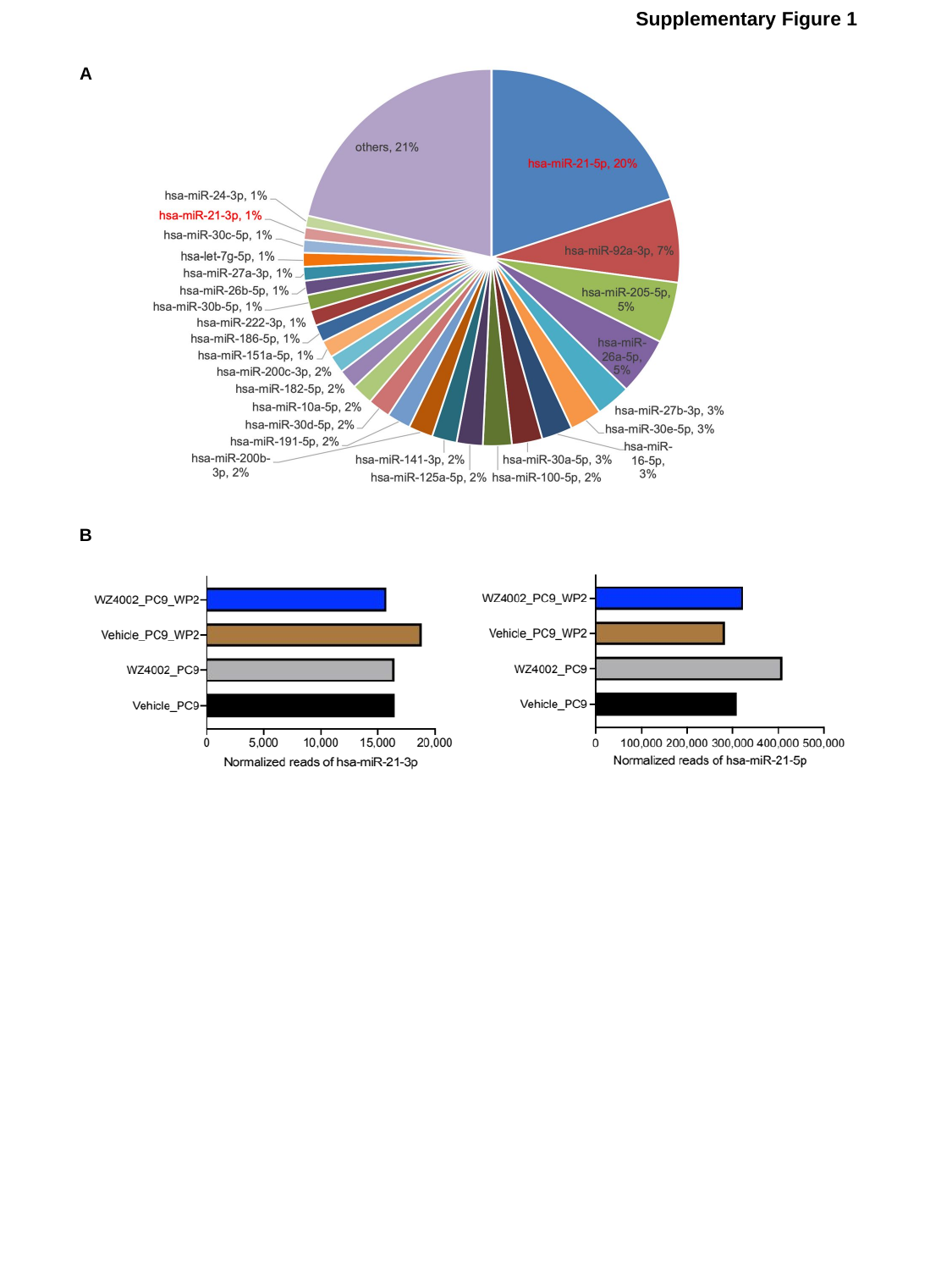

Supplementary Figure 1
A
B

Supplement: Supplementary file 1 — Fig S1 [file 41417_2022_504_MOESM1_ESM.pptx]

## Slide 1
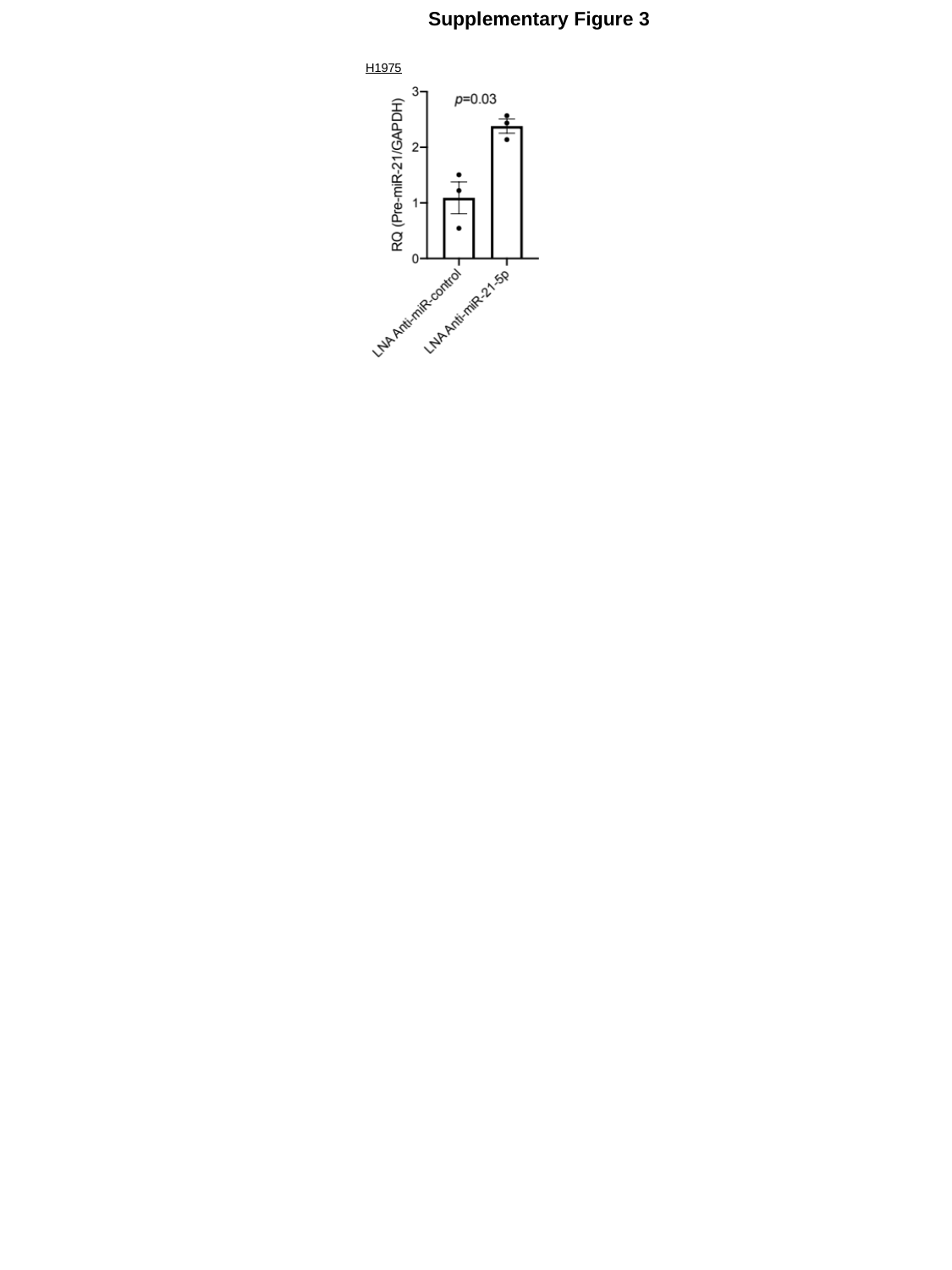

Supplementary Figure 3
H1975

Supplement: Supplementary file 3 — Fig S3 [file 41417_2022_504_MOESM3_ESM.pptx]

## Slide 1
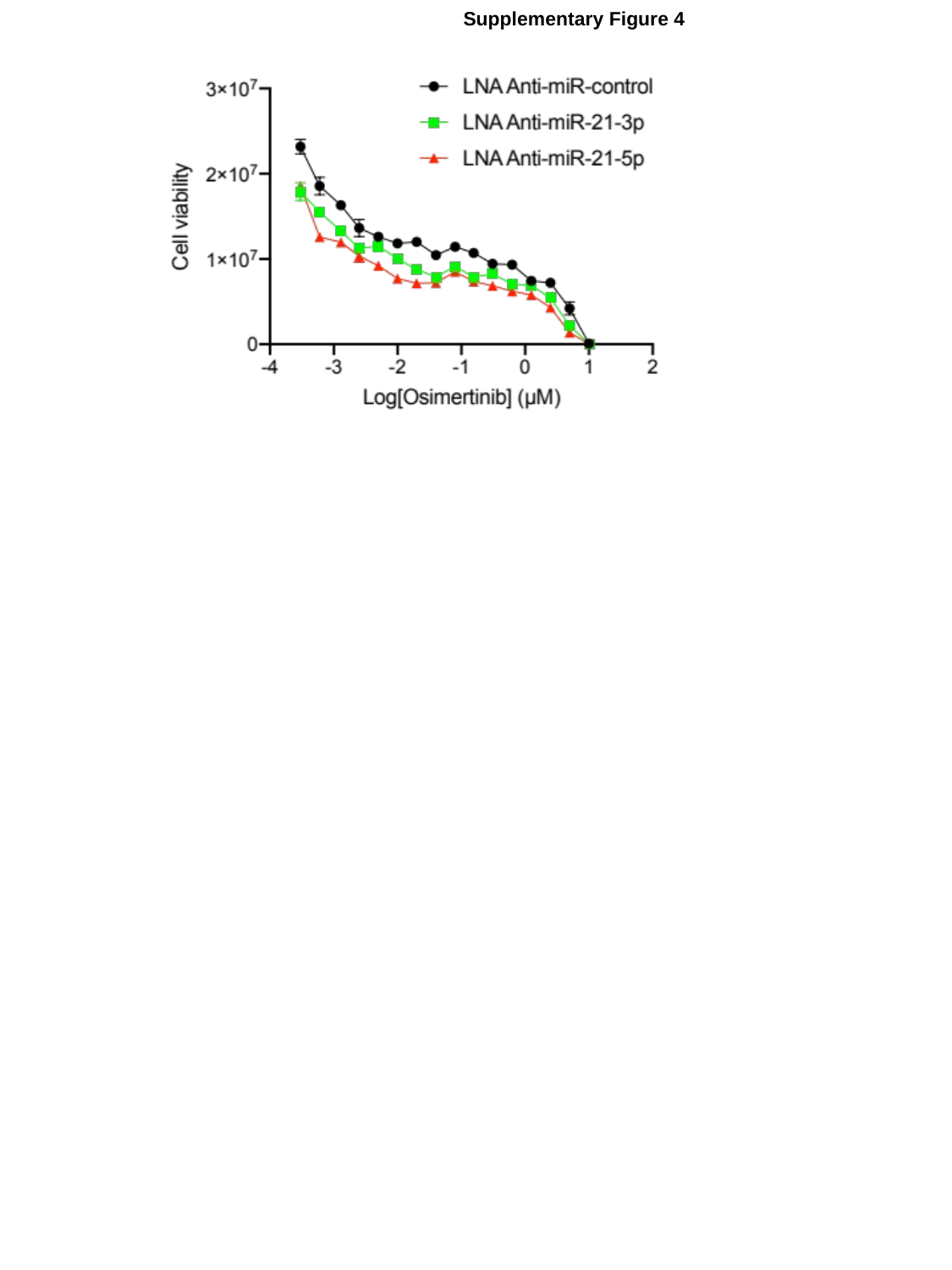

Supplementary Figure 4

Supplement: Supplementary file 4 — Fig S4 [file 41417_2022_504_MOESM4_ESM.pptx]

## Slide 1
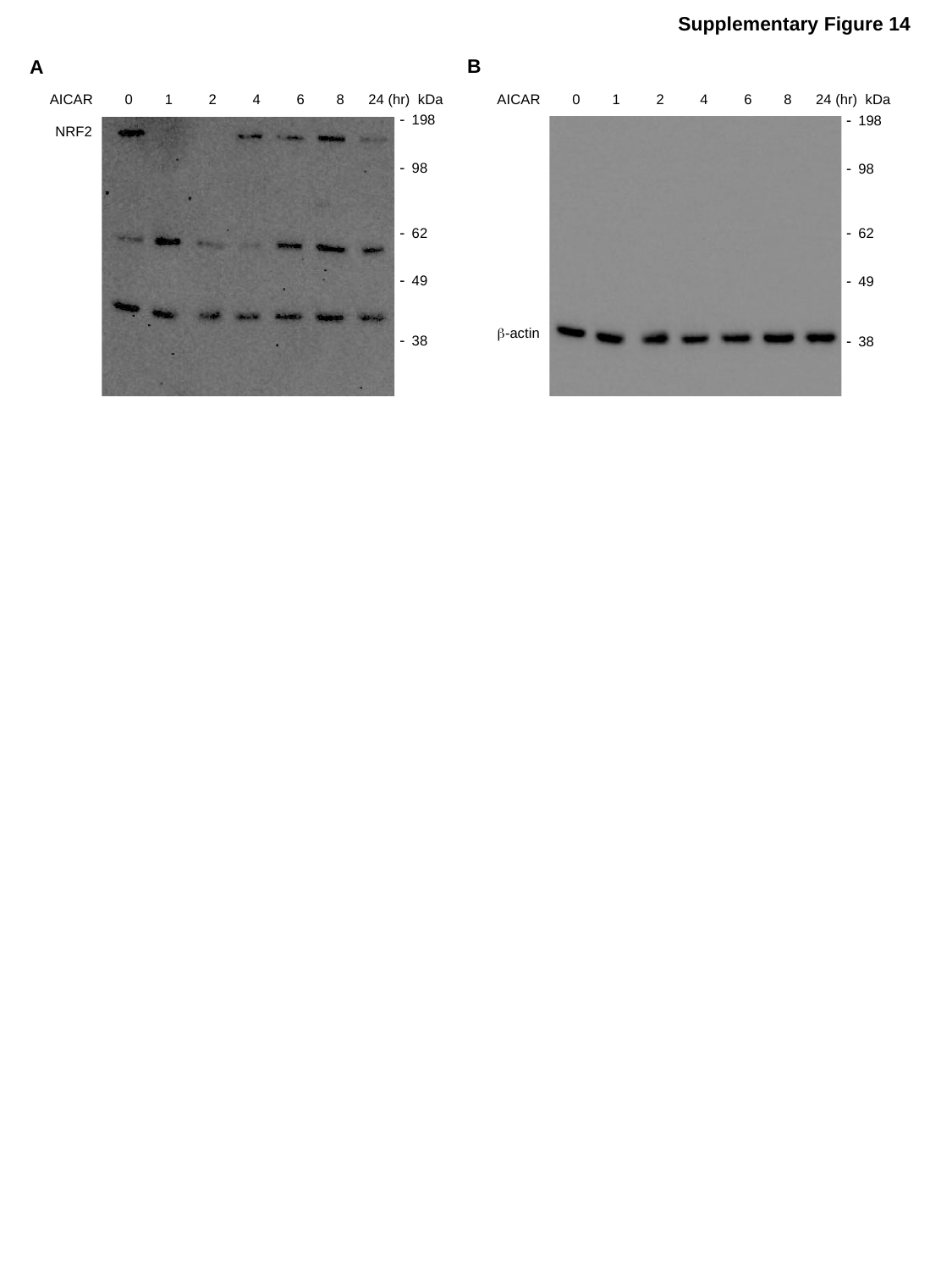

Supplementary Figure 14
B
A
AICAR 0 1 2 4 6 8 24 (hr) kDa
AICAR 0 1 2 4 6 8 24 (hr) kDa
| - 198 |
| --- |
| - 98 |
| - 62 |
| - 49 - 38 |
| - 198 |
| --- |
| - 98 |
| - 62 |
| - 49 - 38 |
NRF2
b-actin

Supplement: Supplementary file 14 — Fig S14 [file 41417_2022_504_MOESM14_ESM.pptx]

## Slide 1
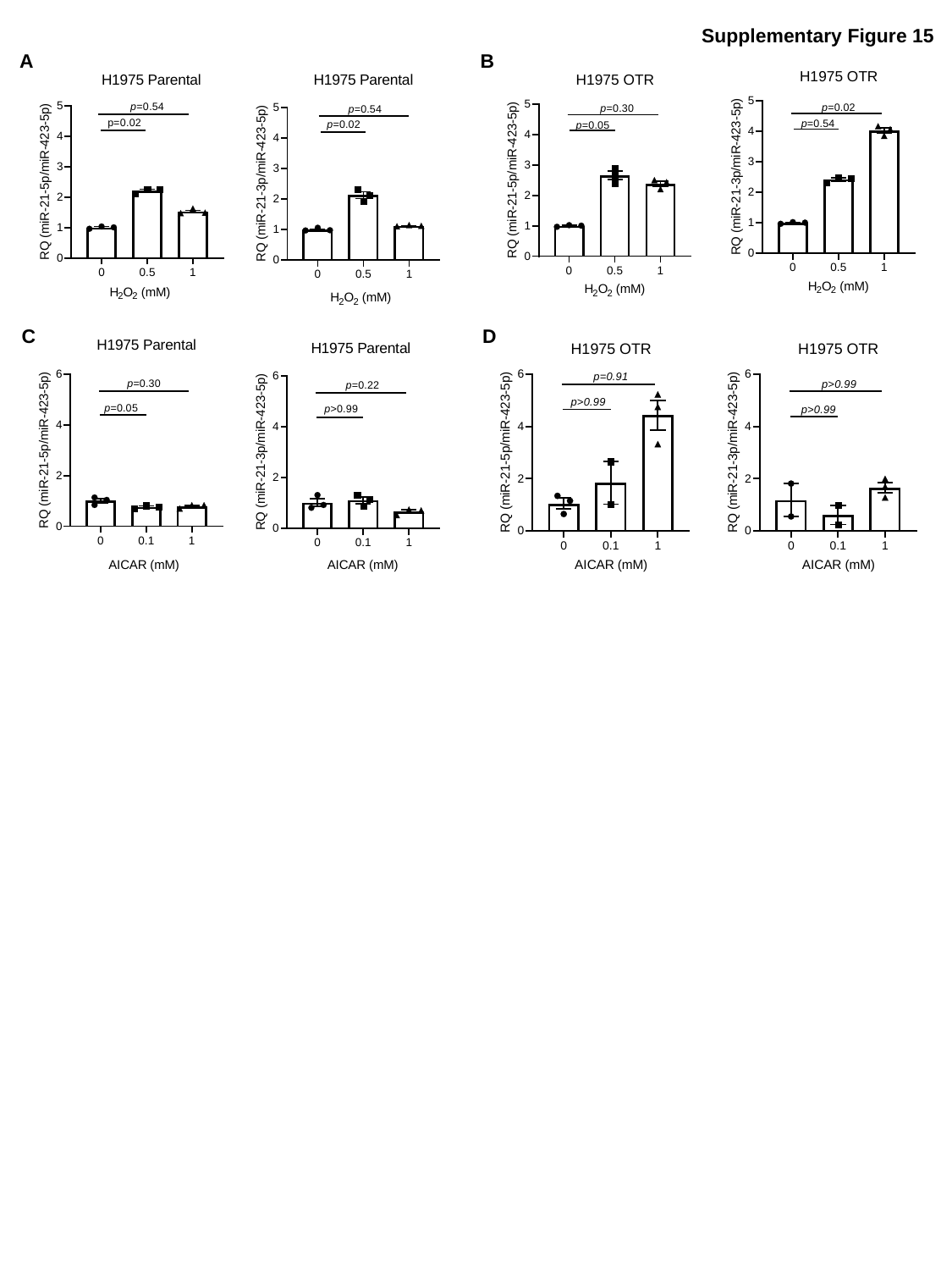

Supplementary Figure 15
A
B
C
D

Supplement: Supplementary file 15 — Fig S15 [file 41417_2022_504_MOESM15_ESM.pptx]
