# Supplementary material for: MicroRNA-21 guide and passenger strand regulation of adenylosuccinate lyase-mediated purine metabolism promotes transition to an EGFR-TKI-tolerant persister state
Source: Cancer Gene Ther. 2022 Jul 15;29(12):1878–94. doi: 10.1038/s41417-022-00504-y (PMC9750876; doi:10.1038/s41417-022-00504-y)
Supplement: Supplementary file 2 — Fig S2 [file 41417_2022_504_MOESM2_ESM.pptx]

## Slide 1
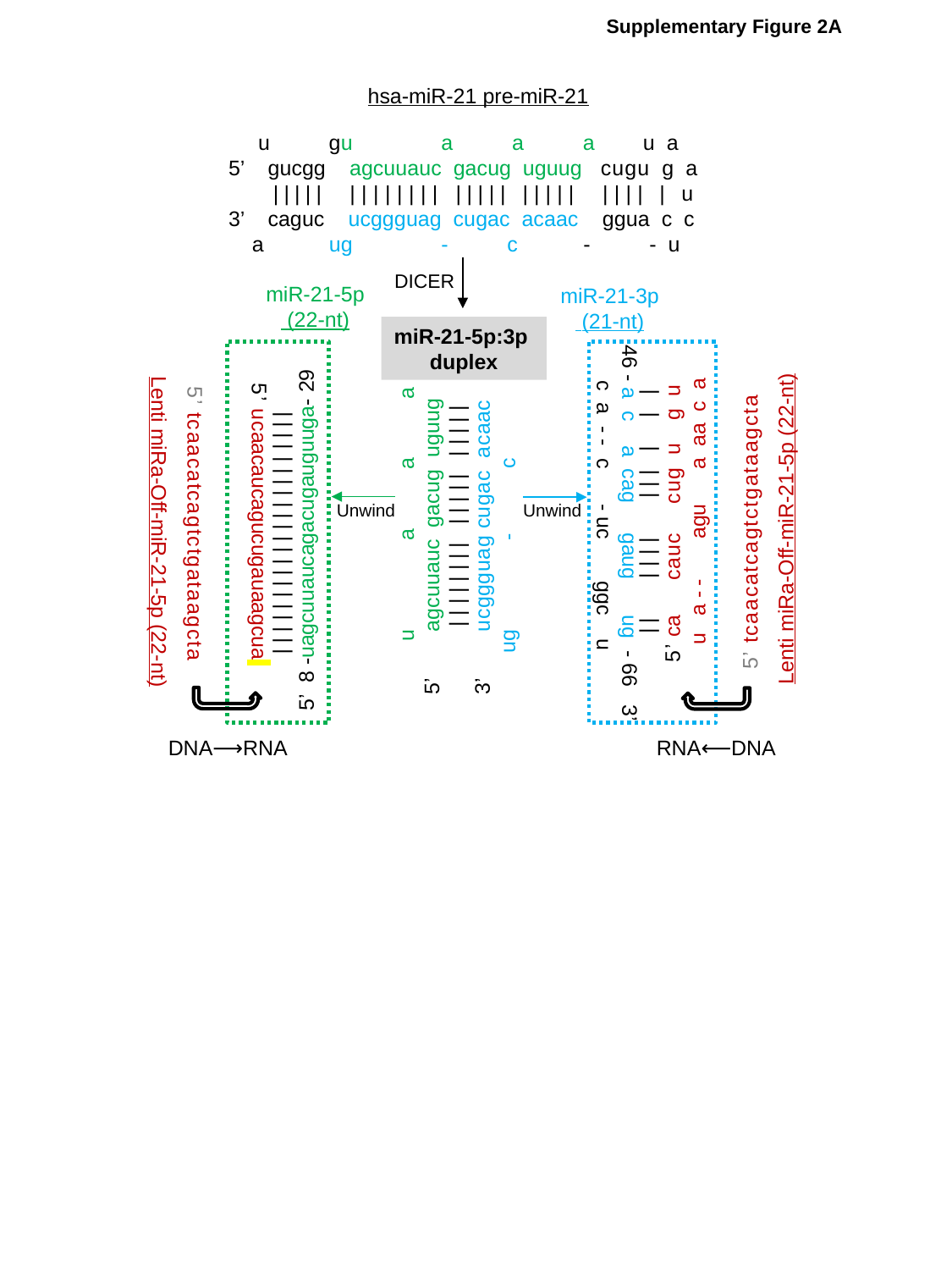

Supplementary Figure 2A
hsa-miR-21 pre-miR-21
 u gu a a a u a
5’ gucgg agcuuauc gacug uguug cugu g a
 ||||| |||||||| ||||| ||||| |||| | u
3’ caguc ucggguag cugac acaac ggua c c
 a ug - c - - u
DICER
miR-21-5p
 (22-nt)
miR-21-3p
 (21-nt)
miR-21-5p:3p
duplex
 || |||| ||| | | |
5’ ca cauc cug u g u
 u a - - agu a aa c a
 u a a a
5’ agcuuauc gacug uguug
 |||||||| ||||| |||||
3’ ucggguag cugac acaac
 ug - c
 5’ tcaacatcagtctgataagcta
 ||||||||||||||||||||||
5’ ucaacaucagucugauaagcua
Unwind
Unwind
46 - a c a cag gaug ug - 66 3’
 c a - - c - uc ggc u
Lenti miRa-Off-miR-21-5p (22-nt)
5’ 8 -uagcuuaucagacugauguuga- 29
Lenti miRa-Off-miR-21-5p (22-nt)
 5’ tcaacatcagtctgataagcta
RNA⟵DNA
DNA⟶RNA

## Slide 2
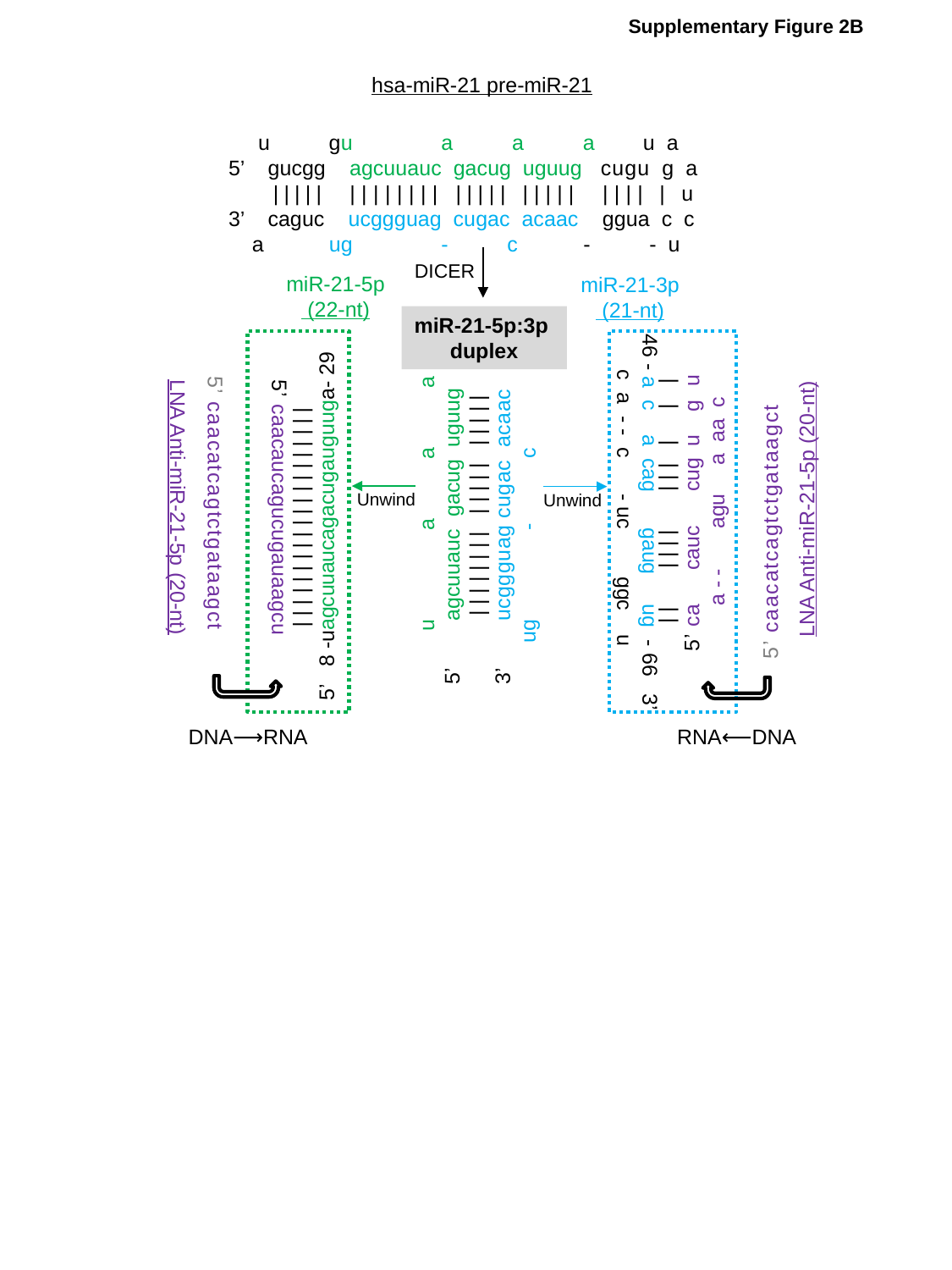

Supplementary Figure 2B
hsa-miR-21 pre-miR-21
DICER
miR-21-5p
 (22-nt)
miR-21-3p
 (21-nt)
miR-21-5p:3p
duplex
 || |||| ||| | | |
5’ ca cauc cug u g u
 a - - agu a aa c
 u a a a
5’ agcuuauc gacug uguug
 |||||||| ||||| |||||
3’ ucggguag cugac acaac
 ug - c
 5’ caacatcagtctgataagct
Unwind
Unwind
 ||||||||||||||||||||
5’ caacaucagucugauaagcu
46 - a c a cag gaug ug - 66 3’
 c a - - c - uc ggc u
5’ 8 -uagcuuaucagacugauguuga- 29
 5’ caacatcagtctgataagct
RNA⟵DNA
DNA⟶RNA
LNA Anti-miR-21-5p (20-nt)
LNA Anti-miR-21-5p (20-nt)
 u gu a a a u a
5’ gucgg agcuuauc gacug uguug cugu g a
 ||||| |||||||| ||||| ||||| |||| | u
3’ caguc ucggguag cugac acaac ggua c c
 a ug - c - - u

## Slide 3
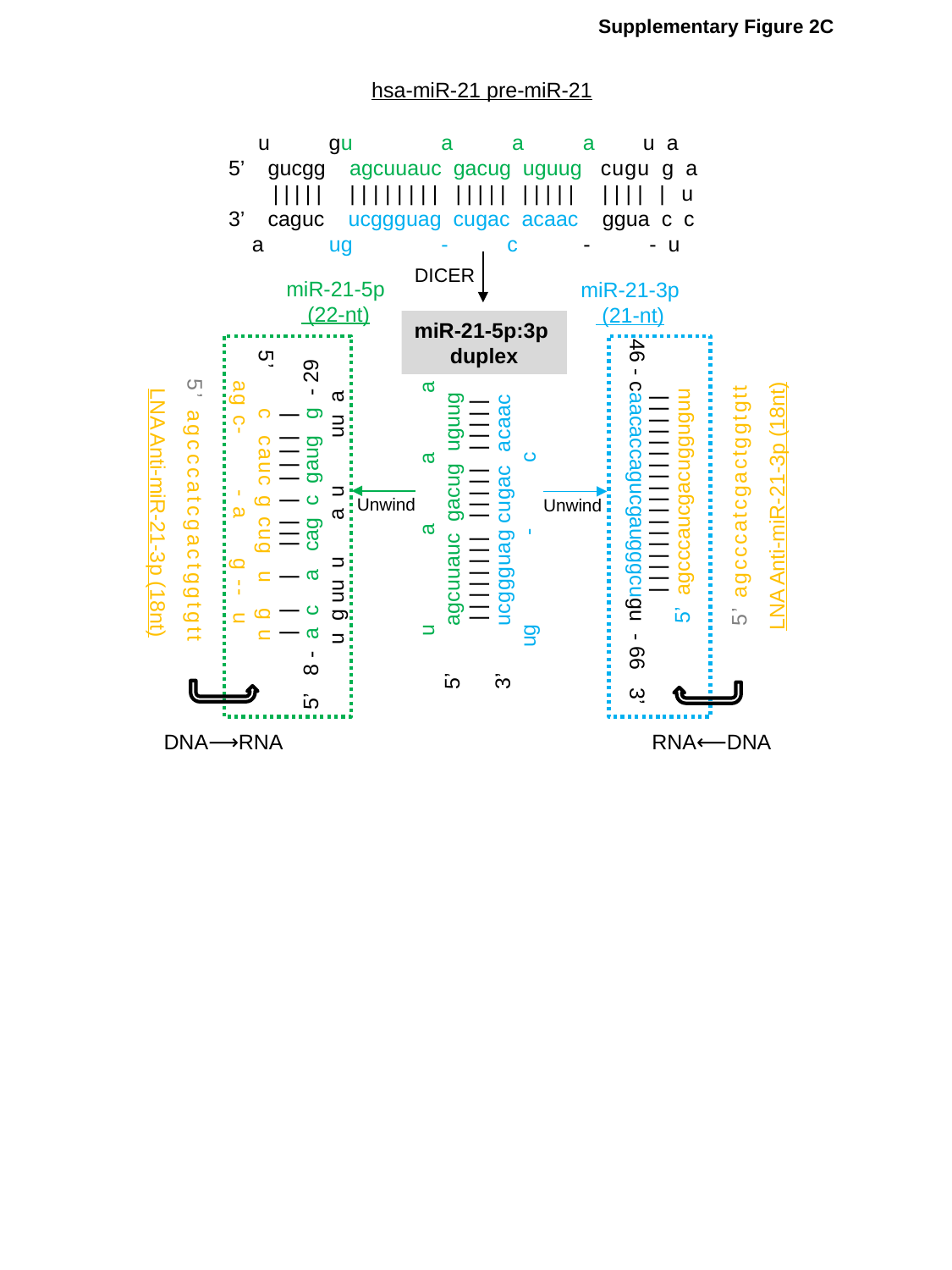

Supplementary Figure 2C
hsa-miR-21 pre-miR-21
DICER
miR-21-5p
 (22-nt)
miR-21-3p
 (21-nt)
miR-21-5p:3p
duplex
 ||||||||||||||||||
 5’ agcccaucgacugguguu
 | |||| | ||| | | |
5’ c cauc g cug u g u
 ag c- - a g -- u
 u a a a
5’ agcuuauc gacug uguug
 |||||||| ||||| |||||
3’ ucggguag cugac acaac
 ug - c
 5’ agcccatcgactggtgtt
Unwind
Unwind
 5’ agcccatcgactggtgtt
5’ 8 - a c a cag c gaug g - 29
 u g uu u a u uu a
46 - caacaccagucgaugggcugu - 66 3’
RNA⟵DNA
DNA⟶RNA
LNA Anti-miR-21-3p (18nt)
LNA Anti-miR-21-3p (18nt)
 u gu a a a u a
5’ gucgg agcuuauc gacug uguug cugu g a
 ||||| |||||||| ||||| ||||| |||| | u
3’ caguc ucggguag cugac acaac ggua c c
 a ug - c - - u

## Slide 4
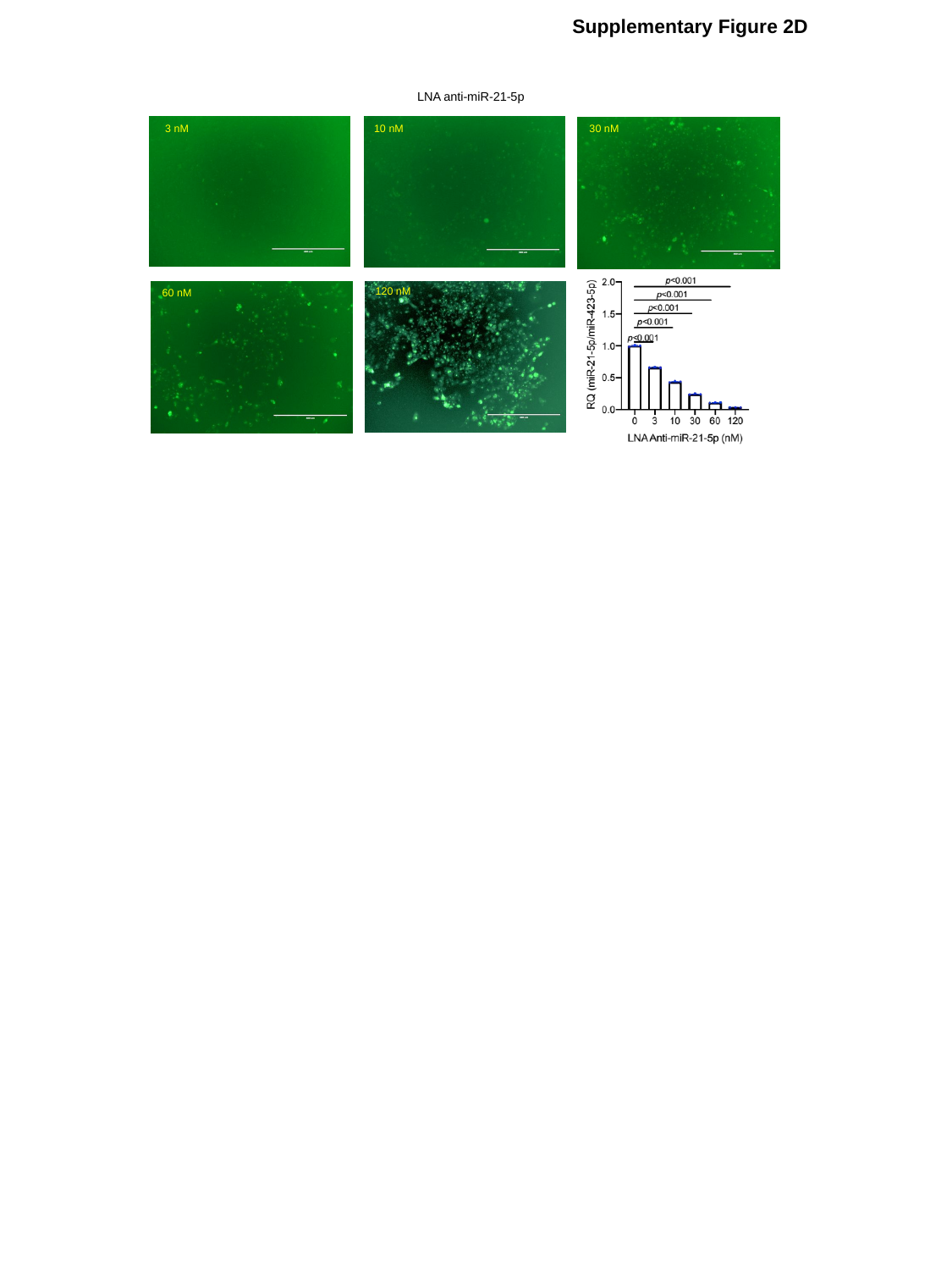

Supplementary Figure 2D
LNA anti-miR-21-5p
10 nM
30 nM
3 nM
120 nM
60 nM
