# Supplementary material for: MicroRNA-21 guide and passenger strand regulation of adenylosuccinate lyase-mediated purine metabolism promotes transition to an EGFR-TKI-tolerant persister state
Source: Cancer Gene Ther. 2022 Jul 15;29(12):1878–94. doi: 10.1038/s41417-022-00504-y (PMC9750876; doi:10.1038/s41417-022-00504-y)
Supplement: Supplementary file 5 — Fig S5 [file 41417_2022_504_MOESM5_ESM.pptx]

## Slide 1
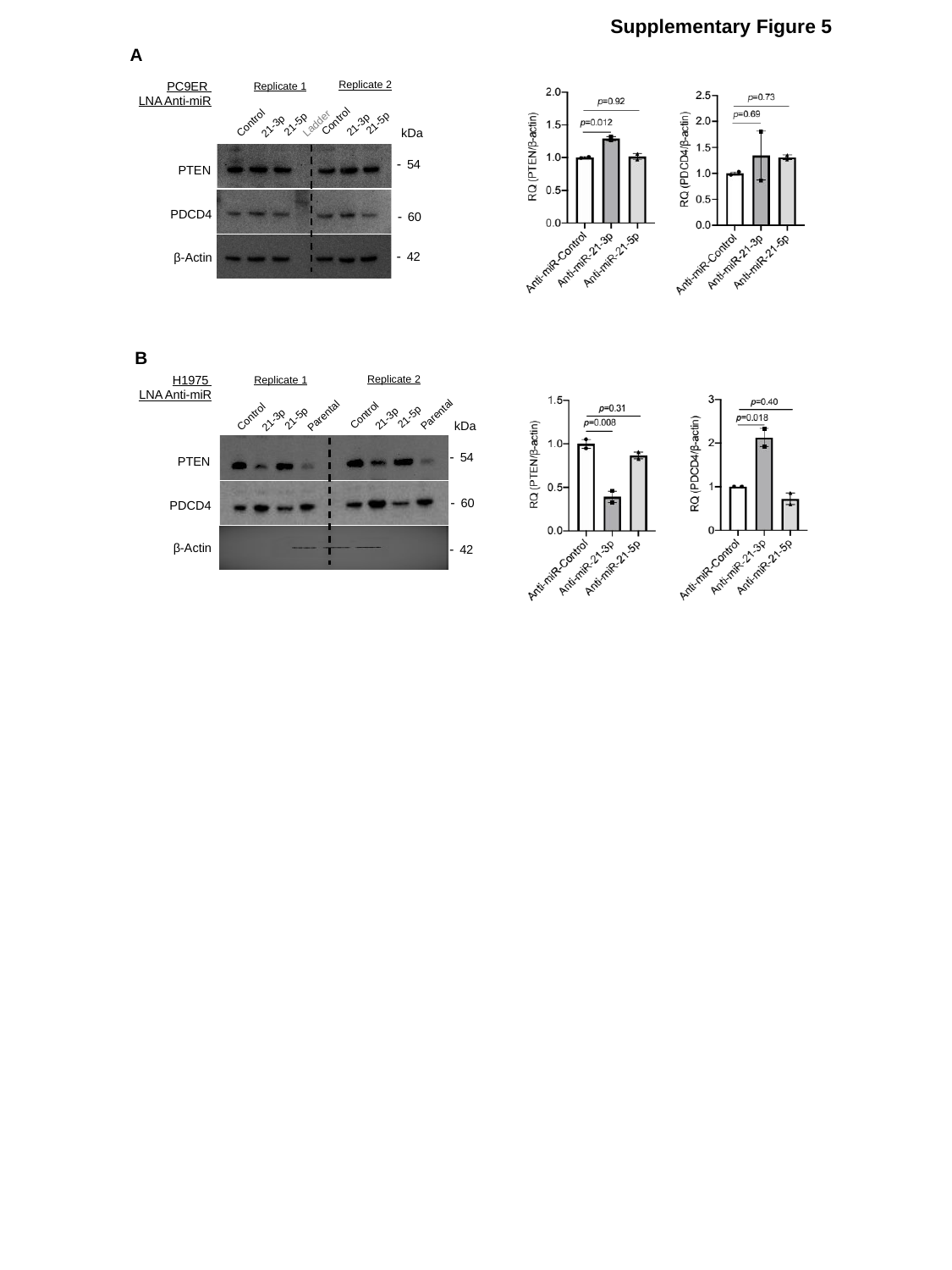

Supplementary Figure 5
A
Replicate 2
21-5p
Control
21-3p
PC9ER
LNA Anti-miR
Replicate 1
21-5p
Control
21-3p
Ladder
kDa
- 54
- 60
- 42
PTEN
PDCD4
β-Actin
B
Replicate 2
21-5p
Control
21-3p
Parental
H1975
LNA Anti-miR
Replicate 1
21-5p
Control
21-3p
Parental
kDa
- 54
- 60
- 42
PTEN
PDCD4
β-Actin
