# Supplementary material for: MicroRNA-21 guide and passenger strand regulation of adenylosuccinate lyase-mediated purine metabolism promotes transition to an EGFR-TKI-tolerant persister state
Source: Cancer Gene Ther. 2022 Jul 15;29(12):1878–94. doi: 10.1038/s41417-022-00504-y (PMC9750876; doi:10.1038/s41417-022-00504-y)
Supplement: Supplementary file 6 — Fig S6 [file 41417_2022_504_MOESM6_ESM.pptx]

## Slide 1
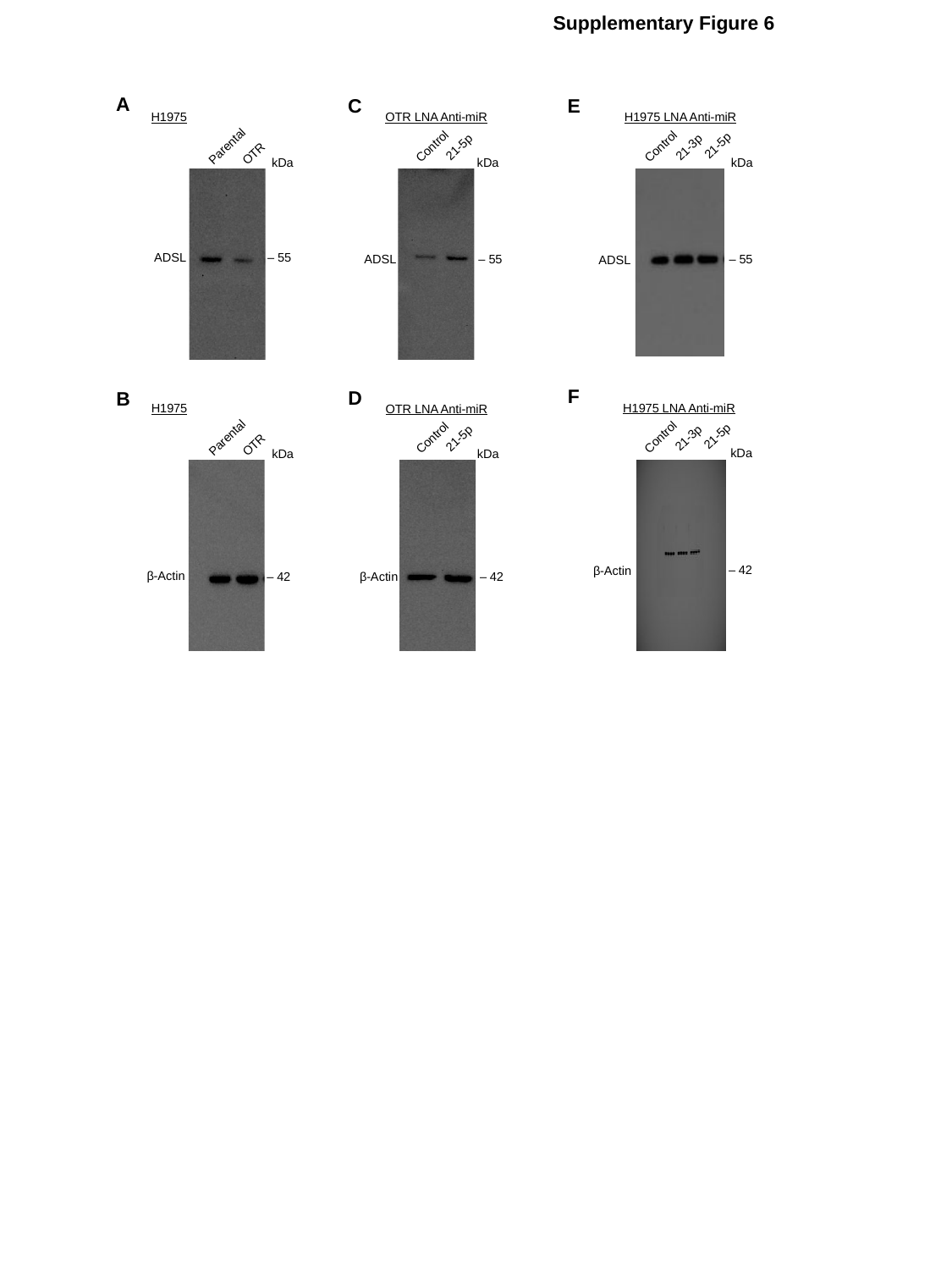

Supplementary Figure 6
A
C
E
H1975
Parental
OTR
kDa
OTR LNA Anti-miR
Control
21-5p
kDa
H1975 LNA Anti-miR
Control
21-3p
kDa
21-5p
– 55
ADSL
– 55
ADSL
ADSL
– 55
F
H1975 LNA Anti-miR
Control
21-3p
kDa
21-5p
D
B
H1975
Parental
OTR
kDa
OTR LNA Anti-miR
Control
21-5p
kDa
β-Actin
– 42
– 42
β-Actin
β-Actin
– 42
