# Supplementary material for: MicroRNA-21 guide and passenger strand regulation of adenylosuccinate lyase-mediated purine metabolism promotes transition to an EGFR-TKI-tolerant persister state
Source: Cancer Gene Ther. 2022 Jul 15;29(12):1878–94. doi: 10.1038/s41417-022-00504-y (PMC9750876; doi:10.1038/s41417-022-00504-y)
Supplement: Supplementary file 7 — Fig S7 [file 41417_2022_504_MOESM7_ESM.pptx]

## Slide 1
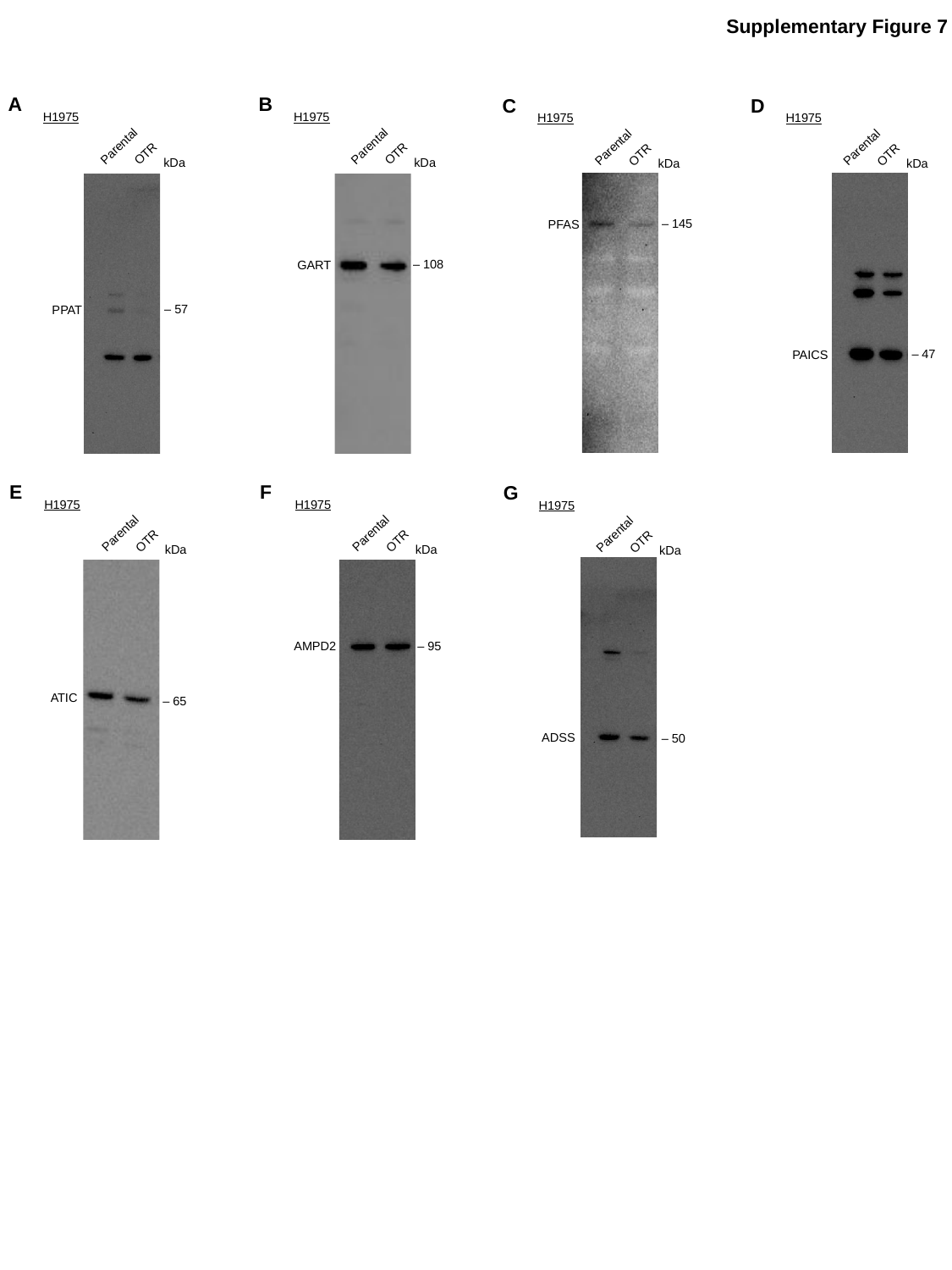

Supplementary Figure 7
A
H1975
Parental
OTR
kDa
B
H1975
Parental
OTR
kDa
D
H1975
Parental
OTR
kDa
C
H1975
Parental
OTR
kDa
– 47
PAICS
– 57
PPAT
– 145
PFAS
– 108
GART
E
H1975
Parental
OTR
kDa
F
H1975
Parental
OTR
kDa
G
H1975
Parental
OTR
kDa
ADSS
– 50
ATIC
– 65
– 95
AMPD2
