# Supplementary material for: MicroRNA-21 guide and passenger strand regulation of adenylosuccinate lyase-mediated purine metabolism promotes transition to an EGFR-TKI-tolerant persister state
Source: Cancer Gene Ther. 2022 Jul 15;29(12):1878–94. doi: 10.1038/s41417-022-00504-y (PMC9750876; doi:10.1038/s41417-022-00504-y)
Supplement: Supplementary file 8 — Fig S8 [file 41417_2022_504_MOESM8_ESM.pptx]

## Slide 1
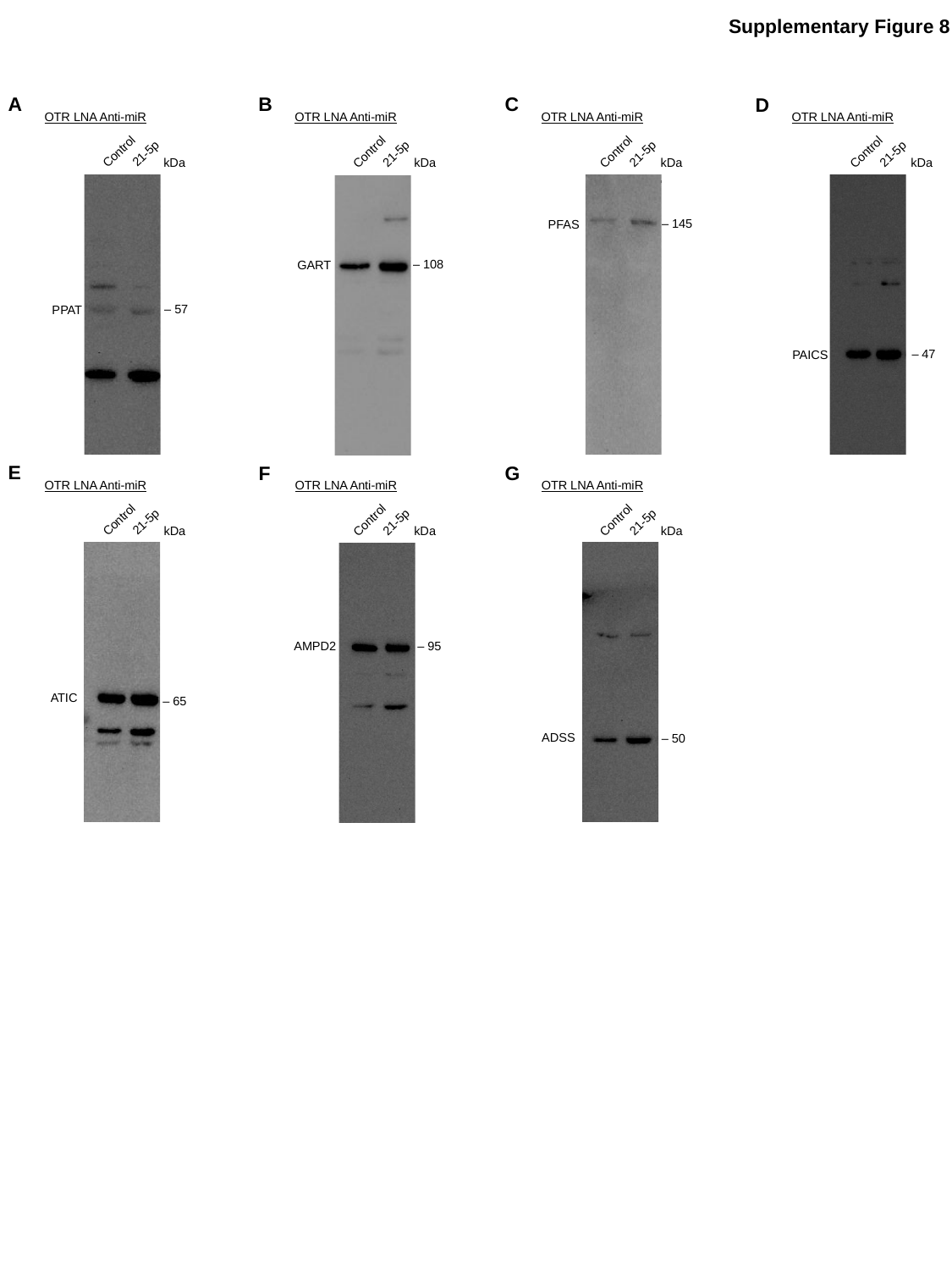

Supplementary Figure 8
A
OTR LNA Anti-miR
Control
21-5p
kDa
C
OTR LNA Anti-miR
Control
21-5p
kDa
B
OTR LNA Anti-miR
Control
21-5p
kDa
D
OTR LNA Anti-miR
Control
21-5p
kDa
– 145
PFAS
– 108
GART
– 57
PPAT
– 47
PAICS
E
OTR LNA Anti-miR
Control
21-5p
kDa
G
OTR LNA Anti-miR
Control
21-5p
kDa
F
OTR LNA Anti-miR
Control
21-5p
kDa
– 95
AMPD2
ATIC
– 65
ADSS
– 50
