# Supplementary material for: MicroRNA-21 guide and passenger strand regulation of adenylosuccinate lyase-mediated purine metabolism promotes transition to an EGFR-TKI-tolerant persister state
Source: Cancer Gene Ther. 2022 Jul 15;29(12):1878–94. doi: 10.1038/s41417-022-00504-y (PMC9750876; doi:10.1038/s41417-022-00504-y)
Supplement: Supplementary file 9 — Fig S9 [file 41417_2022_504_MOESM9_ESM.pptx]

## Slide 1
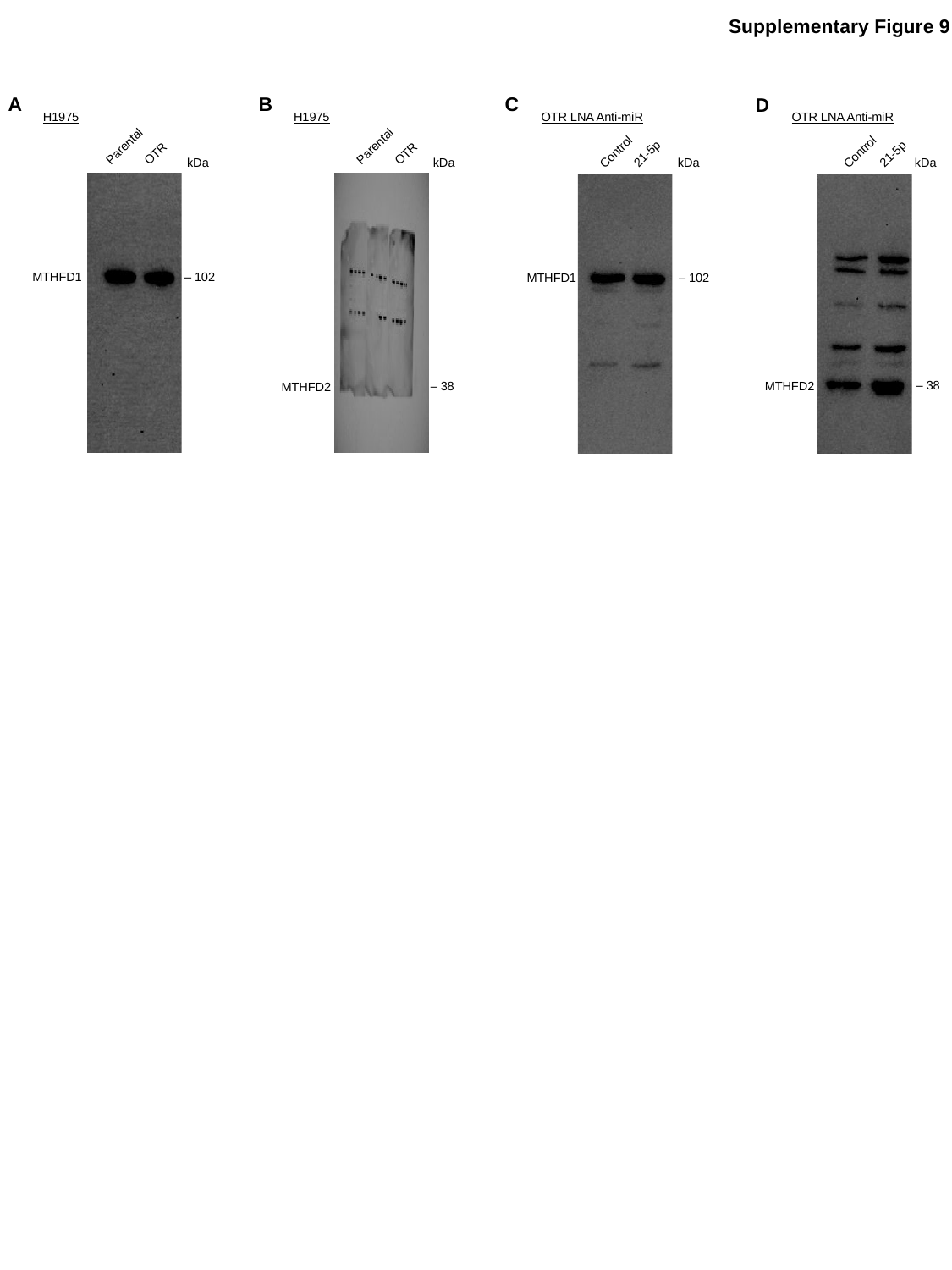

Supplementary Figure 9
A
H1975
Parental
OTR
kDa
B
H1975
Parental
OTR
kDa
C
OTR LNA Anti-miR
Control
21-5p
kDa
D
OTR LNA Anti-miR
Control
21-5p
kDa
– 102
MTHFD1
– 102
MTHFD1
– 38
MTHFD2
– 38
MTHFD2
