# Supplementary material for: MicroRNA-21 guide and passenger strand regulation of adenylosuccinate lyase-mediated purine metabolism promotes transition to an EGFR-TKI-tolerant persister state
Source: Cancer Gene Ther. 2022 Jul 15;29(12):1878–94. doi: 10.1038/s41417-022-00504-y (PMC9750876; doi:10.1038/s41417-022-00504-y)
Supplement: Supplementary file 10 — Fig S10 [file 41417_2022_504_MOESM10_ESM.pptx]

## Slide 1
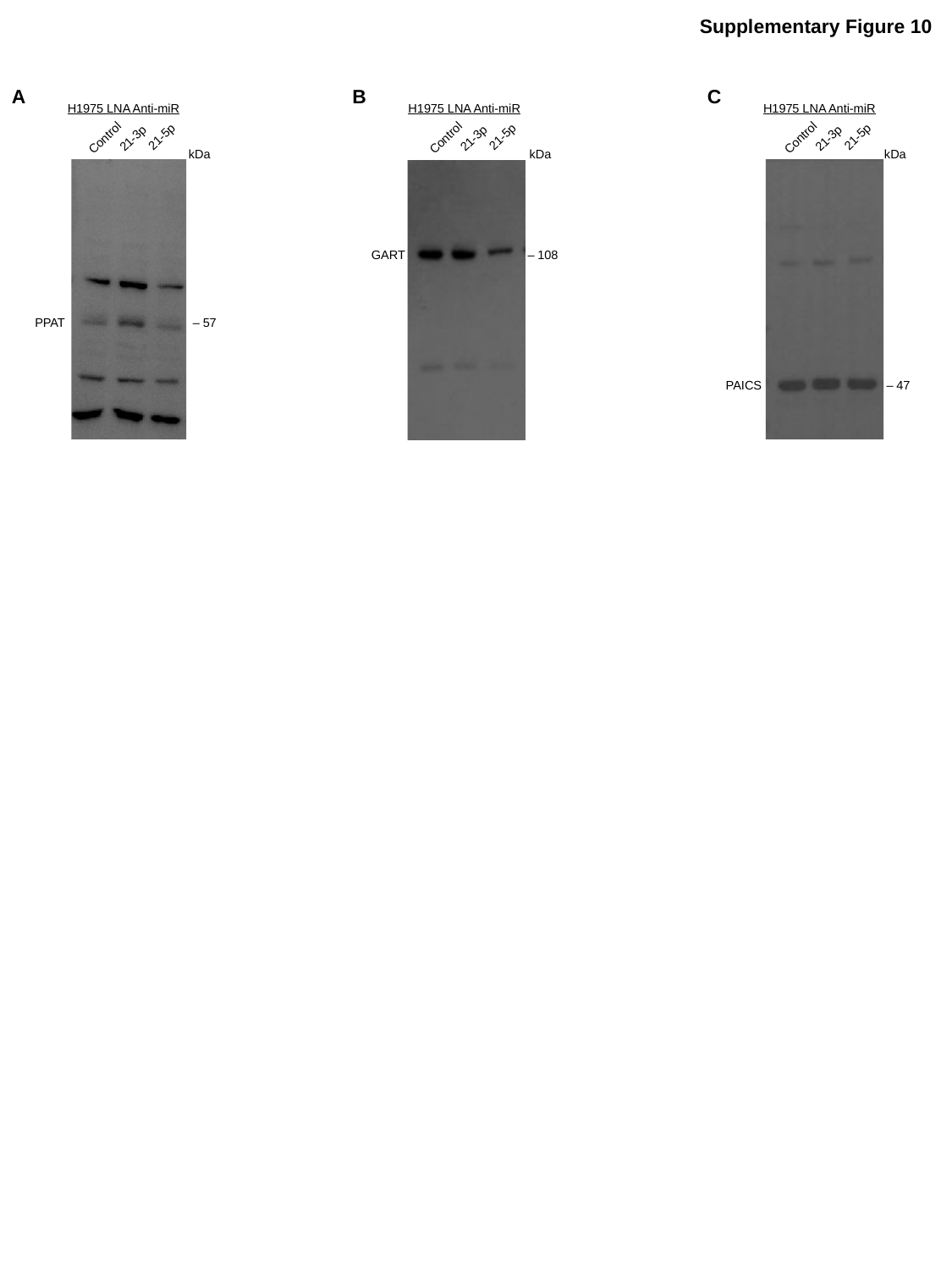

Supplementary Figure 10
B
H1975 LNA Anti-miR
Control
21-3p
kDa
21-5p
C
H1975 LNA Anti-miR
Control
21-3p
kDa
21-5p
A
H1975 LNA Anti-miR
Control
21-3p
kDa
21-5p
PPAT
– 57
GART
– 108
PAICS
– 47
