# Supplementary material for: MicroRNA-21 guide and passenger strand regulation of adenylosuccinate lyase-mediated purine metabolism promotes transition to an EGFR-TKI-tolerant persister state
Source: Cancer Gene Ther. 2022 Jul 15;29(12):1878–94. doi: 10.1038/s41417-022-00504-y (PMC9750876; doi:10.1038/s41417-022-00504-y)
Supplement: Supplementary file 11 — Fig S11 [file 41417_2022_504_MOESM11_ESM.pptx]

## Slide 1
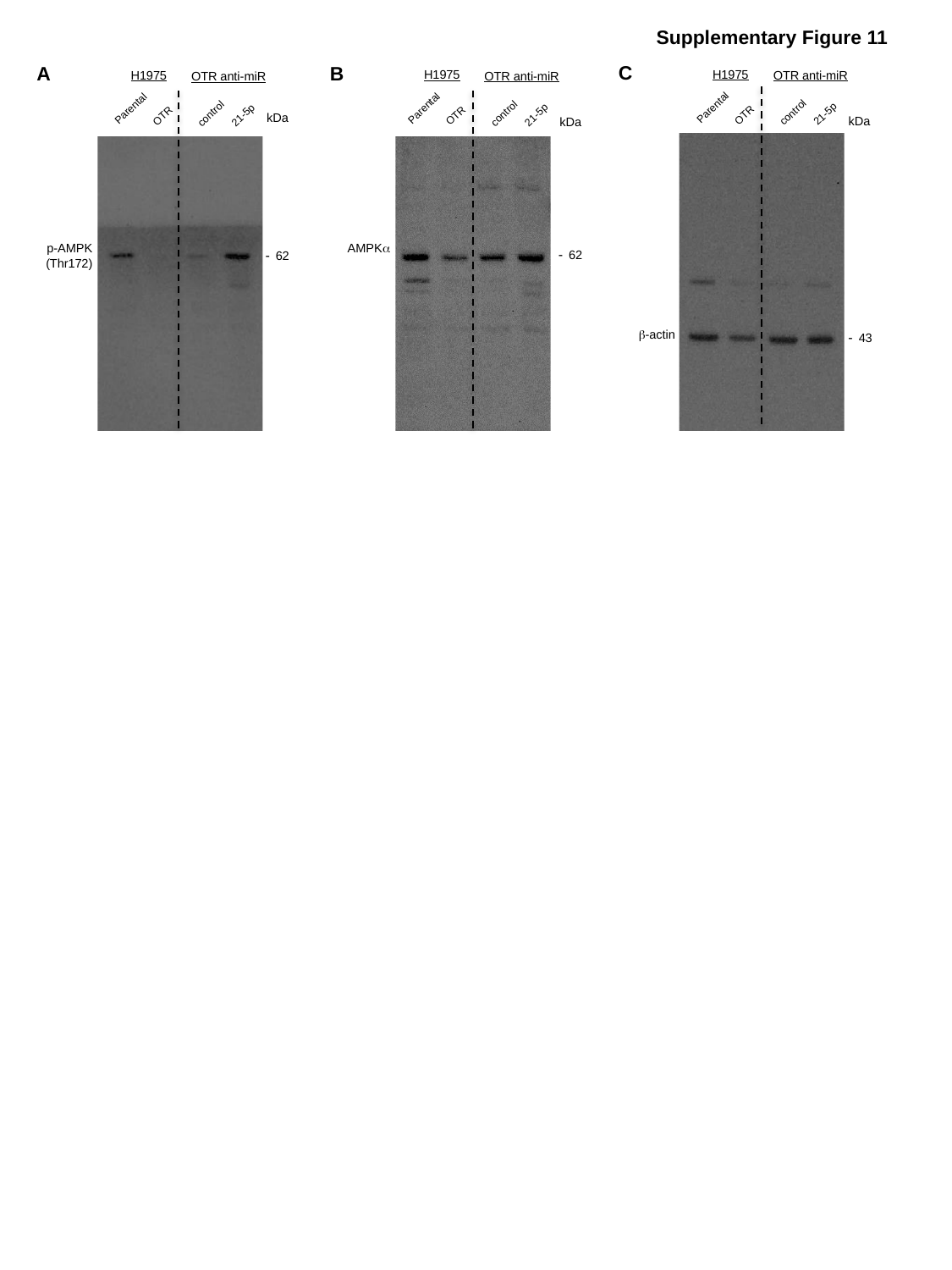

Supplementary Figure 11
C
H1975
OTR anti-miR
Parental
control
21-5p
OTR
B
H1975
OTR anti-miR
Parental
control
21-5p
OTR
A
H1975
OTR anti-miR
Parental
control
21-5p
OTR
kDa
- 62
kDa
kDa
- 62
AMPKa
p-AMPK (Thr172)
b-actin
- 43
