# Supplementary material for: MicroRNA-21 guide and passenger strand regulation of adenylosuccinate lyase-mediated purine metabolism promotes transition to an EGFR-TKI-tolerant persister state
Source: Cancer Gene Ther. 2022 Jul 15;29(12):1878–94. doi: 10.1038/s41417-022-00504-y (PMC9750876; doi:10.1038/s41417-022-00504-y)
Supplement: Supplementary file 12 — Fig S12 [file 41417_2022_504_MOESM12_ESM.pptx]

## Slide 1
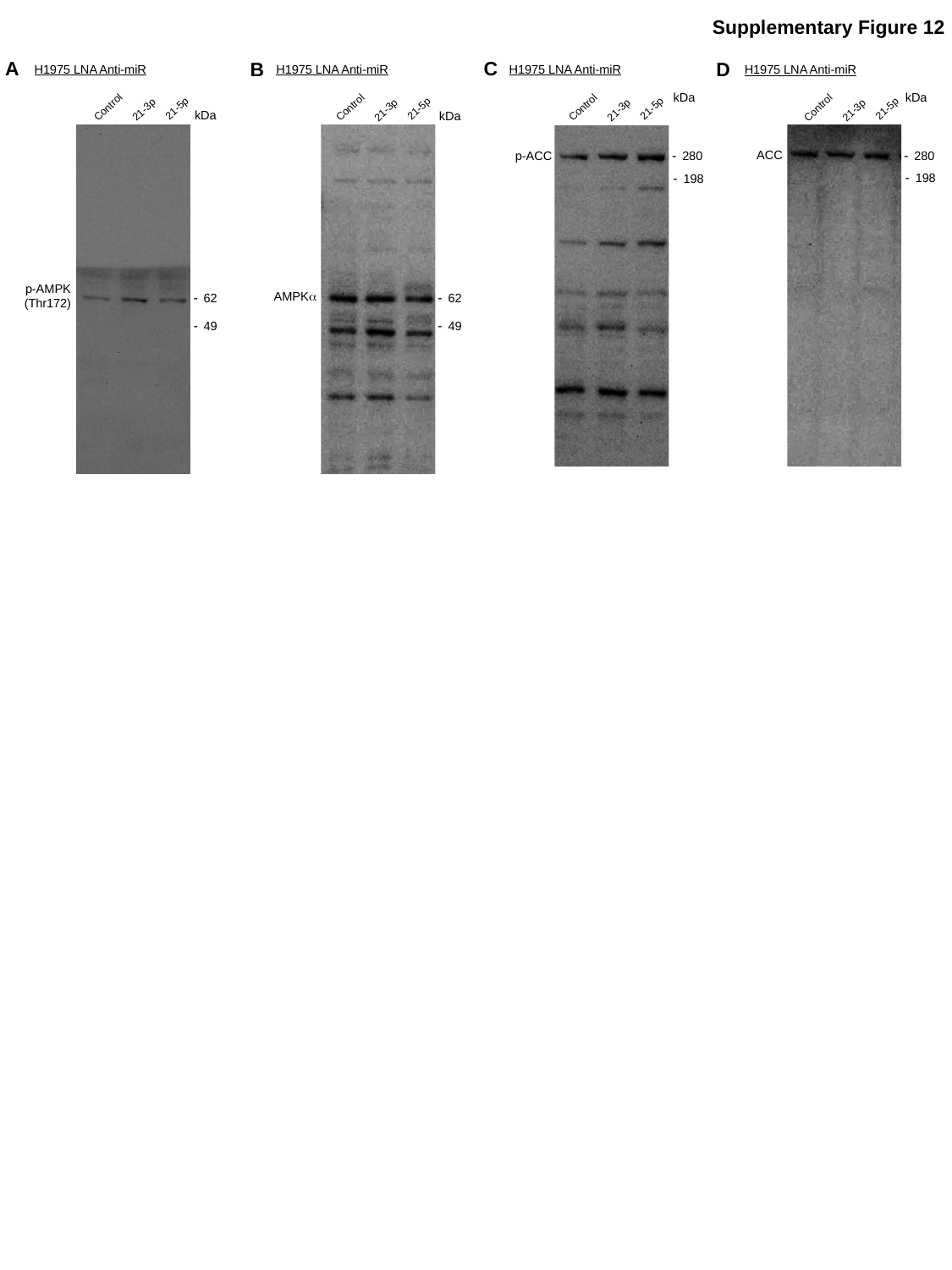

Supplementary Figure 12
C
A
D
B
H1975 LNA Anti-miR
21-5p
Control
21-3p
H1975 LNA Anti-miR
21-5p
Control
21-3p
H1975 LNA Anti-miR
21-5p
Control
21-3p
H1975 LNA Anti-miR
21-5p
Control
21-3p
kDa
- 280
- 198
kDa
- 280
- 198
kDa
kDa
ACC
p-ACC
p-AMPK (Thr172)
AMPKa
- 62
- 49
- 62
- 49
