# Supplementary material for: MicroRNA-21 guide and passenger strand regulation of adenylosuccinate lyase-mediated purine metabolism promotes transition to an EGFR-TKI-tolerant persister state
Source: Cancer Gene Ther. 2022 Jul 15;29(12):1878–94. doi: 10.1038/s41417-022-00504-y (PMC9750876; doi:10.1038/s41417-022-00504-y)
Supplement: Supplementary file 13 — Fig S13 [file 41417_2022_504_MOESM13_ESM.pptx]

## Slide 1
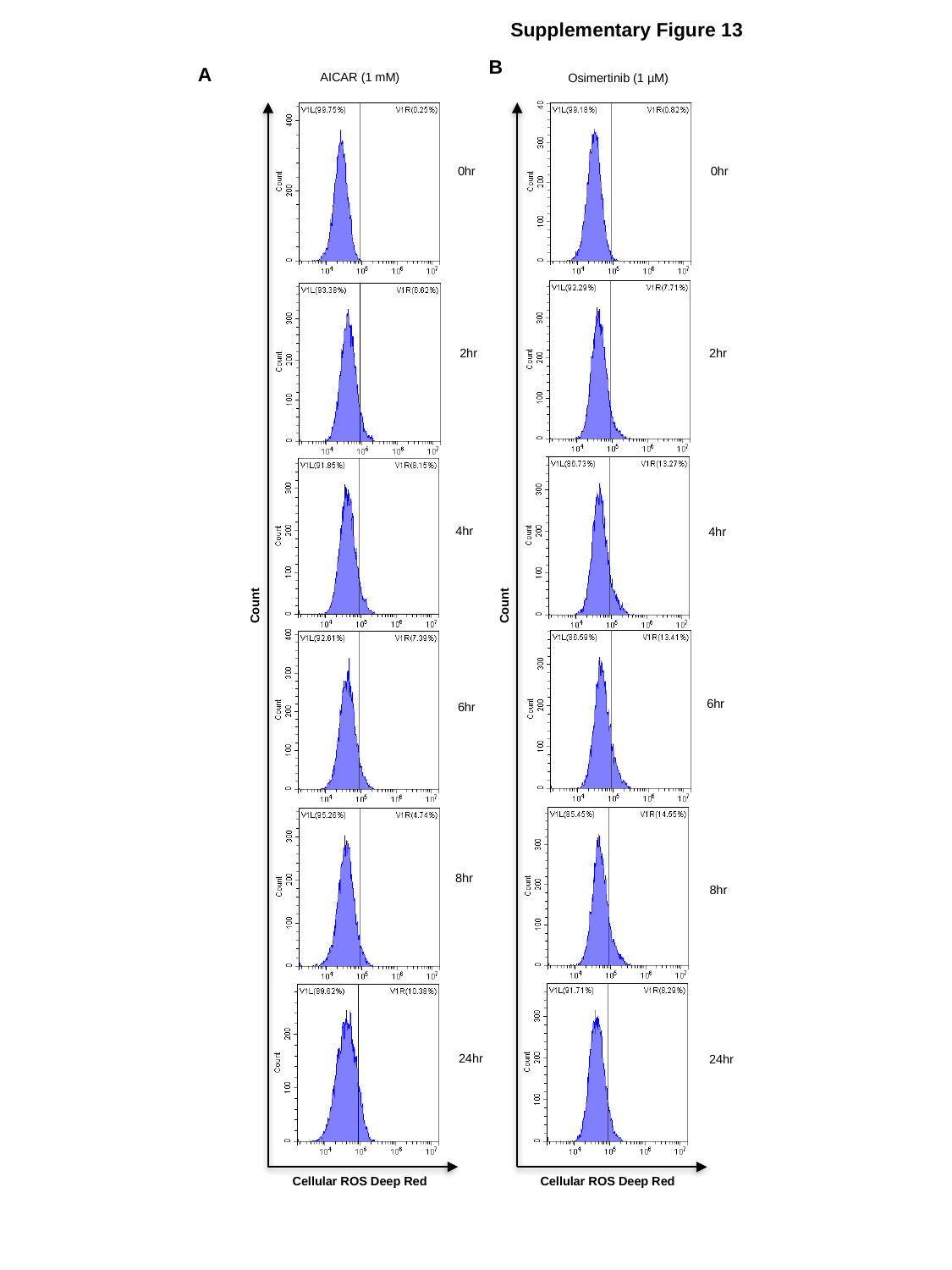

Supplementary Figure 13
AICAR (1 mM)
Osimertinib (1 µM)
B
A
Count
Cellular ROS Deep Red
Count
Cellular ROS Deep Red
0hr
2hr
4hr
6hr
8hr
24hr
0hr
2hr
4hr
6hr
8hr
24hr
