# Supplementary material for: MicroRNA-21 guide and passenger strand regulation of adenylosuccinate lyase-mediated purine metabolism promotes transition to an EGFR-TKI-tolerant persister state
Source: Cancer Gene Ther. 2022 Jul 15;29(12):1878–94. doi: 10.1038/s41417-022-00504-y (PMC9750876; doi:10.1038/s41417-022-00504-y)
Supplement: Supplementary file 16 — Fig S16 [file 41417_2022_504_MOESM16_ESM.pptx]

## Slide 1
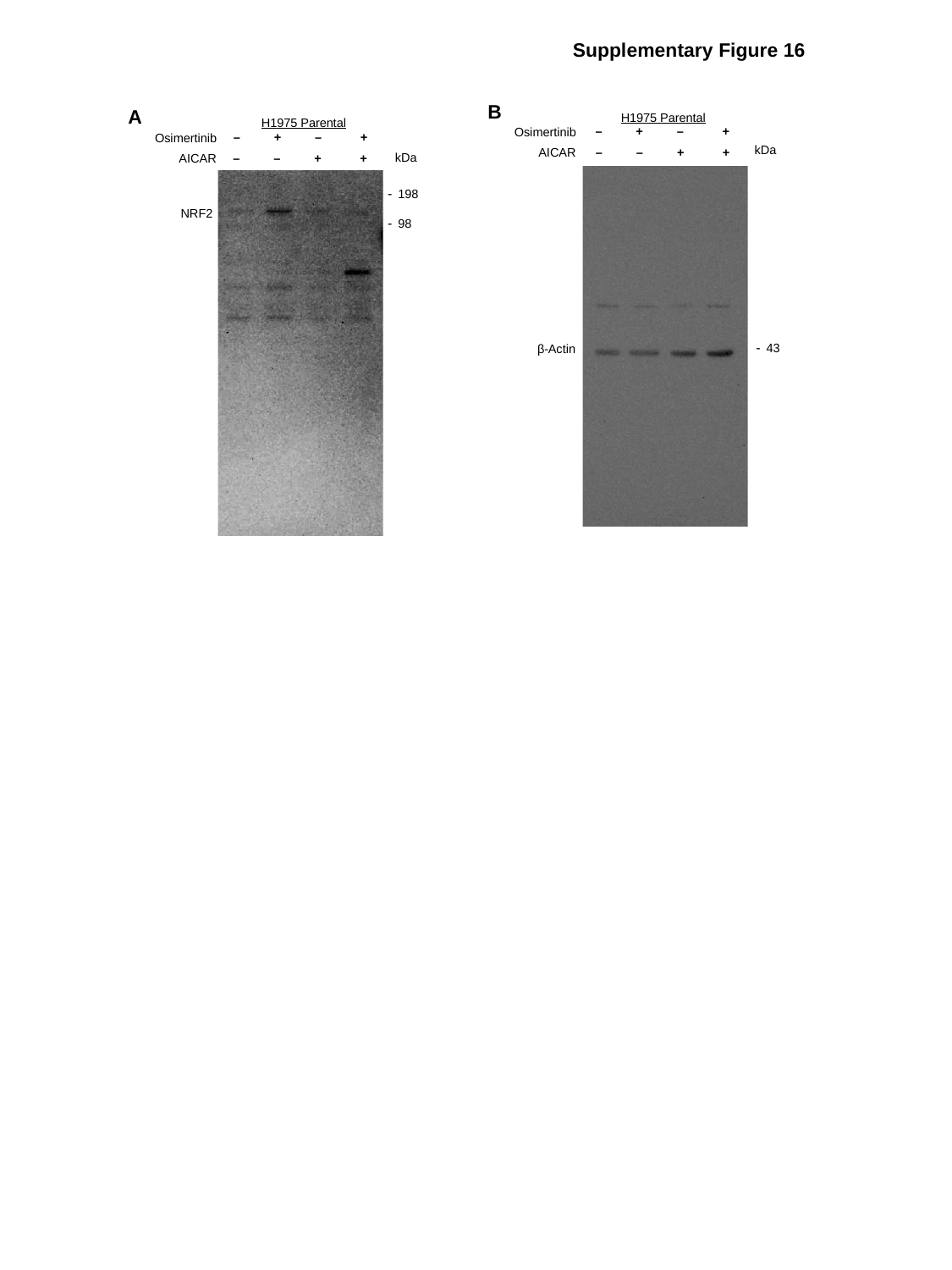

Supplementary Figure 16
B
H1975 Parental
– + –	+
– – +	+
Osimertinib
AICAR
kDa
- 43
β-Actin
A
H1975 Parental
– + –	+
Osimertinib
AICAR
kDa
- 198
- 98
NRF2
– – +	+
