# Supplementary material for: MicroRNA-21 guide and passenger strand regulation of adenylosuccinate lyase-mediated purine metabolism promotes transition to an EGFR-TKI-tolerant persister state
Source: Cancer Gene Ther. 2022 Jul 15;29(12):1878–94. doi: 10.1038/s41417-022-00504-y (PMC9750876; doi:10.1038/s41417-022-00504-y)
Supplement: Supplementary file 17 — Supplemental texts and figures [file 41417_2022_504_MOESM17_ESM.docx]

**Supplementary Information**

**Supplementary Figure 1.** Small RNA-seq analysis of miR-21-5p and miR-21-3p reads in PC9 cells.

(A) Small RNA-seq analysis of miRNAs from PC9 cells. A pie chart showing raw reads of top expressed miRNAs in PC9 cells.

(B) Small RNA-seq analysis of miR-21-5P and miR-21-3p in parental and drug-tolerant persister cells from PC9 treated with a 3^rd^ generation EGFR tyrosine kinase inhibitor WZ4002. The parental PC9 cells were treated with a vehicle (vehicle_PC9) or 1 µM WZ4002 (WZ4002_PC9) for 24 hours. The drug-tolerant persister cells (PC9_WP2) were established by culturing in 300 nM WZ4002 for two weeks. The drug-tolerant persister cells were taken out of the drug for 48 hours prior to being rechallenged with the vehicle (vehicle_PC9_WP2) or WZ4002 (WZ4002_PC9_WP2) for 48 hours. The normalized reads of miR-21-5P and miR-21-3p were shown.

**Supplementary Figure 2A.** The structures and interactions among precursor miR-21, mature miR-21-5p and miR-21-3p as well as miR-21-5p lentiviral inhibitor. The DICER-produced miR-21 duplex (in green and blue) from a hairpin precursor miR-21 (pre-miR-21) is unwound into a guide strand (5p, left in green) and a passenger strand (3p, right in blue), that would be bound by 22-nucleotide (nt) inhibitory lenti miRa-Off-miR-21-5p (in red).

**Supplementary Figure 2B.** The structures and interactions among precursor miR-21, mature miR-21-5p and miR-21-3p as well as locked nucleic acid (LNA) miR-21-5p inhibitor. The DICER-produced miR-21 duplex from a hairpin pre-miR-21 is unwound into a guide strand (5p, left in green) and a passenger strand (3p, right in blue), that are bound to 20-nucleotide (nt) LNA miR-21-5p inhibitor (in purple).

**Supplementary Figure 2C.** The structures and interactions among precursor miR-21, mature miR-21-5p and miR-21-3p as well as locked nucleic acid (LNA) miR-21-3p inhibitor. The DICER-produced miR-21 duplex (in red) from a hairpin pre-miR-21 is unwound into a guide strand (5p, in green) and a passenger strand (3p, in blue), that are bound to 18-nucleotide (nt) LNA miR-21-3p inhibitor (in orange).

**Supplementary Figure 2D.** Fluorescein microscopy and miR-21-5p expression in H1975 cells transfected with a miR-21-5p inhibitor. The cells were transfected with fluorescein-labeled LNA anti-miR-21-5p plasmid (0~120 nM). 36 hours post-transfection, images were taken with a fluorescent microscope and qPCR for miR-21-5p was performed. miR-423-5p was used as an endogenous control. N=3 replicates. Scale bar, 400 µm.

Data are mean ± s.e.m. and were analyzed with Brown-Forsythe and Welch ANOVA.

**Supplementary Figure 3.** qPCR analysis for pre-miR-21 in H1975 parental cells. The H1975 parental cells were transfected with LNA miR-21-5p inhibitor (Anti-miR-21-5p) or LNA scrambled control plasmid (Anti-miR-control) followed by RNA extraction and qPCR analysis. GAPDH was used as an endogenous control. N= 3 replicates.

Data are mean ± s.e.m. and were analyzed with an unpaired two-tailed *t*-test.

**Supplementary Figure 4.** Osimertinib treatment response on H1975 cells treated with LNA inhibitors against miR-21-3p and miR-21-5p. The cells were transfected with LNA miRNA inhibitors against miR-21-3p (Anti-miR-21-3p), miR-21-5p (Anti-miR-21-5p), or a scrambled control miRNA (Anti-miR-control). 48 hours post-transfection, 3,000 cells were plated each well in a 96-well plate and treated with osimertinib for 72 hours followed by cell viability measurement. N=3 replicates.

**Supplementary Figure 5.** Western blot analysis and quantification of PTEN and PDCD4 in parental PC9ER (A) and H1975 (B) cells treated with LNA inhibitors against miR-21-3p, miR-21-5p, and a scrambled miRNA control. β-Actin was used as a loading control. N=2 replicates.

Data are mean ± s.e.m. and were analyzed with one-way ANOVA (A, B).

 **Supplementary Figure 6.** Un-cropped images of the western blots for ADSL in untreated H1975 parental and osimertinib-tolerant (OTR) cells (A), OTR cells treated with LNA miR-21-5p inhibitor (C), or parental cells treated with LNA inhibitors against a scrambled control, miR-21-3p or miR-21-5p (E). β-Actin was used as a loading control (B, D, F).

**Supplementary Figure 7.** Un-cropped images of the western blots for PPAT (A), GART (B), PFAS (C), PAICS (D), ATIC (E), AMPD2 (F), and ADSS (G) in in H1975 parental and osimertinib-tolerant (OTR) cells.

**Supplementary Figure 8.** Un-cropped images of the western blots for PPAT (A), GART (B), PFAS (C), PAICS (D), ATIC (E), AMPD2 (F), and ADSS (G) in H1975 osimertinib-tolerant (OTR) cells treated with LNA inhibitors against miR-21-5p (Anti-miR-21-5p) and a scrambled control (Anti-miR-control).

**Supplementary Figure 9.** Un-cropped images of the western blots for MTHFD1 (A, C) and MTHFD2 (B, D) in H1975 parental cells, osimertinib-tolerant (OTR) cells, and OTR cells treated with LNA inhibitors against miR-21-5p (Anti-miR-21-5p) or a scrambled control (Anti-miR-control).

**Supplementary Figure 10.** Un-cropped images of the western blots for PPAT (A), GART (B), and PAICS (C) in H1975 parental cells treated with LNA inhibitors against miR-21-3p, miR-21-5p, or scrambled control.

**Supplementary Figure 11.** Un-cropped images of the western blots for p-AMPK (A), AMPK (B), and β-actin (C) in H1975 parental cells, OTR cells, and OTR cells treated with the LNA inhibitor against a miRNA control (Anti-miR-control) and miR-21-5p (Anti-miR-21-5p).

**Supplementary Figure 12.** Un-cropped images of the western blots for p-AMPK (A), AMPK (B), p-ACC (C), and ACC (D) in H1975 cells treated with the LNA inhibitor against miR-21-3p (Anti-miR-21-3p), miR-21-5p (Anti-miR-21-5p), and a miRNA control (Anti-miR-control). Total AMPK and total ACC were used as loading controls.

**Supplementary Figure 13.** Representative images for longitudinal flow cytometry analysis of cellular ROS levels in H1975 parental cells. The cells were treated with 1 mM AICAR (A) or 1 µM osimertinib (B) for 0~24 hours before the cellular ROS assay. N=3~4 replicates.

**Supplementary Figure 14.** Un-cropped images of the Western blots for NRF2 (A) in H1975 cells treated with 1 mM AICAR for 0, 1, 2, 4, 6, 8, and 24 hours. β-Actin (B) was used as a loading control.

**Supplementary Figure 15.** qPCR analysis of miR-21-5p and miR-21-3p in H1975 parental and osimertinib-tolerant (OTR) cells. The cells were treated with H_2_O_2_ (A,B) or AICAR (C,D) for four hours followed by RNA extraction and qPCR analysis. miR-423-5p was used as an endogenous control. N= 2~3 replicates.

Data are mean ± s.e.m. and were analyzed with one-way ANOVA (A,B,C,D).

**Supplementary Figure 16.** Un-cropped images of the Western blots for NRF2 (A) and β-Actin (B) in H1975 parental cells treated with vehicle, AICAR (1 mM), osimertinib (1 μM), or both for four hours.

**Supplementary Table 1 Differentially expressed miRNAs in osimertinib and gefitinib tolerant PC9 and HCC827 cells by small RNA-seq**.

| miRNA Name | Fold change_miRNA expression | | Fold change_miRNA reads_other miRNA candidates versus miR-21 | |
| --- | --- | --- | --- | --- |
|  | PC9DTPC  VS PC9 | HCC827DTPC VS HCC827 | PC9DTPC | HCC827DTPC |
| miR-21-5p | 1.43 | 1.55 | 1.000 | 1.000 |
| miR-27a-3p | 1.43 | 1.47 | 0.243 | 0.070 |
| miR-141-3p | 1.47 | 1.56 | 0.259 | 0.094 |
| miR-92a-3p | 0.60 | 1.40 | 0.114 | 0.047 |
| miR-21-3p | 1.55 | 1.11 | 0.069 | 0.031 |
| let-7i-5p | 0.56 | 1.23 | 0.061 | 0.046 |
| miR-100-5p | 0.44 | 0.28 | 0.052 | 0.081 |
| miR-222-3p | 0.75 | 0.46 | 0.054 | 0.033 |
| let-7e-5p | 1.61 | 2.91 | 0.036 | 0.019 |
| miR-92b-3p | 1.05 | 0.53 | 0.029 | 0.010 |
| let-7d-5p | 1.07 | 1.53 | 0.020 | 0.018 |
| let-7b-5p | 1.35 | 2.31 | 0.023 | 0.018 |
| miR-125b-5p | 0.58 | 0.16 | 0.019 | 0.021 |
| miR-423-3p | 1.02 | 1.70 | 0.016 | 0.011 |
| miR-424-5p | 1.88 | 2.24 | 0.013 | 0.020 |
| miR-96-5p | 1.76 | 0.51 | 0.012 | 0.029 |

Note: DTPC, drug-tolerant persister cell.

**Supplementary Table 2 A list of human lung cell lines**

| **Cell lines** | **Pathology** | **Mutant genes and protein sequence** | **Response to gefitinib** | **Response to osimertinib** |
| --- | --- | --- | --- | --- |
| H1975 | lung adenocarcinoma | *EGFR T790M;L858R* | no | partial |
| H1650 | lung adenocarcinoma | *EGFR E746_A750del* | yes | yes |
| PC9 | lung adenocarcinoma | *EGFR E746_A750del* | yes | yes |
| H3255 | lung adenocarcinoma | *EGFR L858R* | yes | yes |
| HCC827 | lung adenocarcinoma | *EGFR E746_A750del* | yes | yes |
| AALE | Lung immortalized cell | n.a. | no | no |

**Supplementary Table 3** **A list of top upregulated metabolites in osimertinib-tolerant cells compared with osimertinib-sensitive cells from H1975 (n=118).**

| Metabolites | FC (T/P) | log2(FC) | *P* value | -log10(*P*) |
| --- | --- | --- | --- | --- |
| Cholesterol | 20.52 | 4.36 | 0.0011023 | 2.96 |
| S-AMP | 5.66 | 2.50 | 8.37E-05 | 4.08 |
| Cytidine | 5.15 | 2.37 | 0.0070461 | 2.15 |
| Acetyl-coa | 4.10 | 2.03 | 0.010717 | 1.97 |
| N-acetyl-glutamate | 3.53 | 1.82 | 0.030486 | 1.52 |
| Glycolate | 3.50 | 1.81 | 0.0052669 | 2.28 |
| dCDP | 3.30 | 1.72 | 0.0067224 | 2.17 |
| CDP-ethanolamine | 3.25 | 1.70 | 0.00089662 | 3.05 |
| Biotin | 3.14 | 1.65 | 0.0071755 | 2.14 |
| UDP-D-glucuronate | 2.88 | 1.53 | 8.03E-05 | 4.10 |
| NADPH | 2.86 | 1.51 | 0.00038477 | 3.41 |
| dTMP | 2.72 | 1.45 | 0.0070456 | 2.15 |
| Inosine | 2.70 | 1.43 | 6.30E-05 | 4.20 |
| Dephospho-CoA | 2.68 | 1.42 | 0.01939 | 1.71 |
| Thymidine | 2.62 | 1.39 | 7.34E-05 | 4.13 |
| Deoxyguanosine | 2.60 | 1.38 | 0.0072093 | 2.14 |
| Urea | 2.60 | 1.38 | 3.06E-05 | 4.51 |
| Methionine sulfoxide | 2.59 | 1.37 | 1.27E-05 | 4.90 |
| Pyridoxine | 2.45 | 1.29 | 2.74E-05 | 4.56 |
| CDP-choline | 2.43 | 1.28 | 0.008458 | 2.07 |
| Glycerol 3-phosphate | 2.37 | 1.24 | 0.0049663 | 2.30 |
| Pyroglutamic acid | 2.33 | 1.22 | 0.00020614 | 3.69 |
| Nicotinamide | 2.33 | 1.22 | 0.00026305 | 3.58 |
| Ornithine | 2.32 | 1.21 | 0.0024081 | 2.62 |
| Adenylyl sulfate | 2.29 | 1.19 | 0.00093902 | 3.03 |
| 1-Methylnicotinamide | 2.28 | 1.19 | 0.015568 | 1.81 |
| Ng,NG-dimethyl-L-arginine | 2.28 | 1.19 | 0.0010667 | 2.97 |
| 2,3-Diphosphoglyceric acid | 2.25 | 1.17 | 0.018918 | 1.72 |
| Lysine | 2.21 | 1.15 | 8.71E-05 | 4.06 |
| Acetoacetate | 2.21 | 1.15 | 0.0044441 | 2.35 |
| D-4'-Phosphopantothenate | 2.20 | 1.14 | 0.00040258 | 3.40 |
| Folate | 2.19 | 1.13 | 5.99E-06 | 5.22 |
| Deoxyuridine | 2.19 | 1.13 | 0.0022092 | 2.66 |
| L-Homocysteic acid | 2.15 | 1.11 | 0.029496 | 1.53 |
| Citrulline | 2.12 | 1.08 | 9.84E-06 | 5.01 |
| Uric acid | 2.11 | 1.08 | 1.47E-05 | 4.83 |
| Trehalose-6-phosphate | 2.10 | 1.07 | 0.0013527 | 2.87 |
| Ribose-5-phosphate | 2.08 | 1.06 | 0.00054086 | 3.27 |
| Arginine | 2.08 | 1.05 | 6.07E-05 | 4.22 |
| Phenylpyruvate | 2.07 | 1.05 | 0.025458 | 1.59 |
| Gluconolactone | 2.06 | 1.05 | 0.015595 | 1.81 |
| 2,3-dihydroxybenzoic acid | 2.06 | 1.05 | 0.017864 | 1.75 |
| UMP | 2.04 | 1.03 | 0.042935 | 1.37 |
| D-gluconate | 2.03 | 1.02 | 0.037601 | 1.42 |
| Citrate | 2.01 | 1.00 | 0.030869 | 1.51 |
| Xanthosine | 1.97 | 0.98 | 0.0053601 | 2.27 |
| Taurodeoxycholic acid | 1.95 | 0.97 | 0.0094011 | 2.03 |
| Sucrose | 1.95 | 0.96 | 0.00056853 | 3.25 |
| Histidine | 1.94 | 0.95 | 1.93E-05 | 4.71 |
| Quinolinate | 1.92 | 0.94 | 0.00034335 | 3.46 |
| Xanthine | 1.90 | 0.93 | 0.0028351 | 2.55 |
| Pyrophosphate | 1.89 | 0.92 | 0.012069 | 1.92 |
| Glycerate | 1.89 | 0.92 | 0.00011325 | 3.95 |
| 7-Methylguanosine | 1.88 | 0.91 | 0.0005081 | 3.29 |
| P-hydroxybenzoate | 1.84 | 0.88 | 0.00025698 | 3.59 |
| Adenine | 1.82 | 0.86 | 5.71E-05 | 4.24 |
| Tyrosine | 1.81 | 0.86 | 0.0001653 | 3.78 |
| NADP | 1.80 | 0.85 | 0.0011825 | 2.93 |
| Methionine | 1.80 | 0.85 | 0.00023411 | 3.63 |
| N1-Methyl-2_2-pyridone-3_5-carboximide | 1.79 | 0.84 | 0.016193 | 1.79 |
| dATP | 1.78 | 0.83 | 0.029186 | 1.53 |
| UDP | 1.78 | 0.83 | 0.00061192 | 3.21 |
| Succinate | 1.77 | 0.83 | 4.74E-05 | 4.32 |
| Methylmalonic acid | 1.76 | 0.81 | 5.42E-05 | 4.27 |
| 3-Methylphenylacetic acid | 1.76 | 0.81 | 0.019192 | 1.72 |
| Serine | 1.76 | 0.81 | 8.02E-05 | 4.10 |
| Cellobiose | 1.76 | 0.81 | 0.0030171 | 2.52 |
| Hydroxyphenylpyruvate | 1.75 | 0.81 | 0.00018051 | 3.74 |
| Methylcysteine | 1.74 | 0.80 | 0.00053585 | 3.27 |
| Glyceric acid 1,3-biphosphate | 1.74 | 0.80 | 0.0077766 | 2.11 |
| 2-dehydro-D-gluconate | 1.72 | 0.79 | 0.0015694 | 2.80 |
| Valine | 1.72 | 0.78 | 0.00025176 | 3.60 |
| dTDP | 1.71 | 0.78 | 0.0063847 | 2.19 |
| 2-Oxoglutarate | 1.70 | 0.77 | 5.24E-05 | 4.28 |
| ADP | 1.70 | 0.76 | 0.0043378 | 2.36 |
| Kynurenine | 1.68 | 0.75 | 0.0074812 | 2.13 |
| Phenylpropiolic acid | 1.68 | 0.75 | 0.00014105 | 3.85 |
| dGDP | 1.68 | 0.75 | 0.0027695 | 2.56 |
| 1-Methyl-Histidine | 1.67 | 0.74 | 0.00058707 | 3.23 |
| IDP | 1.67 | 0.74 | 0.0091311 | 2.04 |
| Purine | 1.67 | 0.74 | 0.00046021 | 3.34 |
| N6-Acetyl-L-lysine | 1.66 | 0.73 | 0.0032193 | 2.49 |
| Tryptophan | 1.66 | 0.73 | 0.0018357 | 2.74 |
| 2-Deoxy-D-glucose 6-phosphate | 1.66 | 0.73 | 0.0015252 | 2.82 |
| Phosphoenolpyruvate | 1.63 | 0.70 | 0.00075041 | 3.12 |
| Allantoin | 1.59 | 0.66 | 0.0025305 | 2.60 |
| Nicotinate | 1.58 | 0.66 | 0.015152 | 1.82 |
| Threonine | 1.55 | 0.64 | 0.00016201 | 3.79 |
| Hydroxyproline | 1.54 | 0.62 | 0.00018224 | 3.74 |
| Indole | 1.53 | 0.62 | 0.03106 | 1.51 |
| Sedoheptulose 1,7-bisphosphate | 1.53 | 0.61 | 0.01142 | 1.94 |
| Glycine | 1.52 | 0.61 | 0.00895 | 2.05 |
| Spermine | 1.51 | 0.59 | 0.017761 | 1.75 |
| AMP | 1.50 | 0.58 | 0.043699 | 1.36 |
| Hydroxyisocaproic acid | 1.48 | 0.57 | 0.00025194 | 3.60 |
| glutamine | 1.48 | 0.56 | 0.00017383 | 3.76 |
| N-carbamoyl-L-aspartate | 1.47 | 0.56 | 0.016328 | 1.79 |
| Asparagine | 1.47 | 0.55 | 0.0012804 | 2.89 |
| FAD | 1.46 | 0.55 | 0.0090607 | 2.04 |
| Hypoxanthine | 1.44 | 0.53 | 0.038571 | 1.41 |
| Beta-D-Fructose 1,6-bisphosphate | 1.44 | 0.53 | 0.028553 | 1.54 |
| Phenylalanine | 1.42 | 0.51 | 0.0042506 | 2.37 |
| Indole-3-carboxylic acid | 1.40 | 0.48 | 0.016775 | 1.78 |
| 2-Hydroxy-2-methylbutanedioic acid | 1.37 | 0.45 | 0.0034965 | 2.46 |
| Glutathione disulfide | 1.37 | 0.45 | 0.010855 | 1.96 |
| Carbamoyl phosphate | 1.36 | 0.45 | 0.0085478 | 2.07 |
| Aminoadipic acid | 1.36 | 0.44 | 0.032383 | 1.49 |
| Allantoate | 1.34 | 0.42 | 0.036452 | 1.44 |
| L-Leucine | 1.33 | 0.41 | 0.0014392 | 2.84 |
| Lactate | 1.32 | 0.40 | 0.028739 | 1.54 |
| Dimethylglycine | 1.31 | 0.39 | 0.030126 | 1.52 |
| DL-Pipecolic acid | 1.30 | 0.38 | 0.024394 | 1.61 |
| Pantothenate | 1.29 | 0.37 | 0.033598 | 1.47 |
| Glyoxylate | 1.24 | 0.31 | 0.013883 | 1.86 |
| Proline | 1.24 | 0.31 | 0.03191 | 1.50 |
| Phenyllactic acid | 1.24 | 0.31 | 0.040546 | 1.39 |
| Orotidine 5'-phosphate | 1.23 | 0.30 | 0.0036988 | 2.43 |

**Supplementary Table 4** A list of top downregulated metabolites in osimertinib-tolerant cells compared with osimertinib-sensitive cells from H1975 (n=12).

| Metabolites | FC (T/P) | log2(FC) | *P* value | -log10(*P*) |
| --- | --- | --- | --- | --- |
| Coenzyme A | 0.13 | -2.96 | 7.59E-06 | 5.12 |
| Glutathione | 0.14 | -2.87 | 5.33E-06 | 5.27 |
| Aminoimidazole carboxamide ribonucleotide (AICAR) | 0.37 | -1.45 | 0.0006778 | 3.17 |
| S-adenosylhomocysteine | 0.45 | -1.15 | 0.019461 | 1.71 |
| Aspartate | 0.53 | -0.92 | 0.00023074 | 3.64 |
| N-Acetyl-D-glucosamine | 0.56 | -0.82 | 0.0091476 | 2.04 |
| Carnitine | 0.58 | -0.79 | 1.12E-06 | 5.95 |
| Maleic acid | 0.59 | -0.76 | 0.00067406 | 3.17 |
| Alpha-ketoisovaleric acid | 0.60 | -0.74 | 0.0009032 | 3.04 |
| Fumarate | 0.60 | -0.73 | 0.00030551 | 3.52 |
| 4-Aminobutyrate | 0.61 | -0.71 | 0.00068459 | 3.16 |
| Sedoheptulose 7-phosphate | 0.62 | -0.69 | 0.018529 | 1.73 |
| L-malic acid | 0.70 | -0.51 | 0.0016075 | 2.79 |
| Nicotinamide adenine dinucleotide (NAD) | 0.74 | -0.42 | 0.020692 | 1.68 |

T: tolerant cells; P: parental cells.

**Supplementary Table 5** **A list of top upregulated and downregulated metabolites in osimertinib-tolerant cells with miR-21 knockdown (anti21) compared with scrambled control cells (antictrl) from H1975 (n=103)**.

| **Metabolites** | **FC (anti21 vs antictrl)** | **log2(FC)** | ***P* value** | **-log10(*P*)** |
| --- | --- | --- | --- | --- |
| acadesine | 6.8311 | 2.7721 | 6.88E-05 | 4.1627 |
| hypoxanthine | 3.8573 | 1.9476 | 3.53E-08 | 7.4522 |
| D-gluconate | 3.0298 | 1.5992 | 6.87E-08 | 7.1632 |
| putrescine | 3.0023 | 1.5861 | 0.0014285 | 2.8451 |
| anthranilate | 2.7912 | 1.4809 | 0.022037 | 1.6568 |
| Gluconolactone | 2.5514 | 1.3513 | 3.85E-08 | 7.4145 |
| 6-phospho-D-gluconate | 2.4115 | 1.27 | 0.00013224 | 3.8786 |
| 6-Phosphonoglucono-D-lactone | 2.331 | 1.221 | 0.00044707 | 3.3496 |
| Ascorbic acid | 2.0628 | 1.0446 | 0.023897 | 1.6217 |
| AICAR | 2.0014 | 1.001 | 0.0054758 | 2.2616 |
| IMP | 1.8428 | 0.88189 | 8.34E-05 | 4.0791 |
| cis-Aconitic acid | 1.6931 | 0.75971 | 2.41E-05 | 4.6176 |
| 2,3-dihydroxybenzoic acid | 1.5542 | 0.63615 | 0.0079496 | 2.0997 |
| D-glucarate | 1.5166 | 0.60083 | 0.0040653 | 2.3909 |
| L-Dihydroorotic acid | 1.4578 | 0.54383 | 0.0014293 | 2.8449 |
| 4-aminobutyrate | 1.4403 | 0.5264 | 0.001095 | 2.9606 |
| citrate | 1.3847 | 0.46962 | 0.0059189 | 2.2278 |
| aspartate | 1.2668 | 0.34123 | 0.024276 | 1.6148 |
| 2-Isopropylmalic acid | 1.2462 | 0.31751 | 0.034572 | 1.4613 |
| histidine | 0.84003 | -0.25149 | 0.0003724 | 3.429 |
| succinate | 0.83659 | -0.2574 | 0.00021312 | 3.6714 |
| tyrosine | 0.83635 | -0.25782 | 0.00035824 | 3.4458 |
| hydroxyproline | 0.83553 | -0.25924 | 0.020941 | 1.679 |
| phenylalanine | 0.83437 | -0.26124 | 3.41E-06 | 5.4672 |
| indole | 0.82892 | -0.27069 | 0.02673 | 1.573 |
| tryptophan | 0.82558 | -0.27653 | 6.26E-05 | 4.2034 |
| methionine | 0.8236 | -0.27998 | 0.0032691 | 2.4856 |
| 2-Aminooctanoic acid | 0.82263 | -0.28168 | 0.00037824 | 3.4222 |
| Phosphorylcholine | 0.81576 | -0.29378 | 0.00014698 | 3.8327 |
| ADP | 0.80391 | -0.3149 | 0.034056 | 1.4678 |
| Methionine sulfoxide | 0.80345 | -0.31572 | 0.0012471 | 2.9041 |
| Sucrose | 0.80265 | -0.31716 | 3.40E-05 | 4.4681 |
| trehalose-6-Phosphate | 0.79944 | -0.32294 | 0.048516 | 1.3141 |
| octulose 8-phosphate/octulose 1-phosphate | 0.797 | -0.32735 | 0.00025475 | 3.5939 |
| glutathione disulfide | 0.79611 | -0.32895 | 0.00046389 | 3.3336 |
| folate | 0.79169 | -0.33699 | 1.24E-06 | 5.9055 |
| arginine | 0.79059 | -0.33899 | 0.00041499 | 3.382 |
| p-hydroxybenzoate | 0.78765 | -0.34437 | 0.00066503 | 3.1772 |
| UDP-D-glucuronate | 0.78678 | -0.34596 | 0.030888 | 1.5102 |
| carnitine | 0.78392 | -0.35122 | 0.00025866 | 3.5873 |
| Uric acid | 0.7785 | -0.36123 | 0.0016502 | 2.7825 |
| proline | 0.77268 | -0.37206 | 1.30E-05 | 4.8862 |
| Deoxyribose 5-phosphate | 0.77098 | -0.37523 | 5.45E-05 | 4.2639 |
| glutamate | 0.7603 | -0.39536 | 1.35E-05 | 4.8704 |
| xanthine | 0.75921 | -0.39742 | 0.001213 | 2.9161 |
| dGDP | 0.75675 | -0.40212 | 0.0076107 | 2.1186 |
| taurine | 0.75431 | -0.40678 | 3.97E-05 | 4.401 |
| xanthosine | 0.75122 | -0.41269 | 0.0063584 | 2.1967 |
| UDP | 0.75121 | -0.41271 | 0.035559 | 1.449 |
| pyridoxine | 0.74503 | -0.42463 | 2.55E-05 | 4.5932 |
| 2-Hydroxyglutarate | 0.74125 | -0.43196 | 1.33E-06 | 5.8753 |
| Ng,NG-dimethyl-L-arginine | 0.7345 | -0.44516 | 0.016599 | 1.7799 |
| IDP | 0.72517 | -0.46361 | 0.0025183 | 2.5989 |
| glucose-6-phosphate | 0.72388 | -0.46618 | 0.0023208 | 2.6344 |
| D-Ribose 5-phosphate | 0.72378 | -0.46638 | 0.0020851 | 2.6809 |
| Kynurenine | 0.71442 | -0.48516 | 0.010474 | 1.9799 |
| DL-Pipecolic acid | 0.71106 | -0.49195 | 2.92E-06 | 5.5352 |
| lysine | 0.70724 | -0.49973 | 0.00014009 | 3.8536 |
| 2-Ketohexanoic acid | 0.70443 | -0.50548 | 2.27E-05 | 4.6444 |
| Pyroglutamic acid | 0.70252 | -0.50938 | 2.50E-08 | 7.6024 |
| Carbamoyl phosphate | 0.70174 | -0.51099 | 1.01E-05 | 4.9944 |
| nicotinamide | 0.69965 | -0.5153 | 7.43E-06 | 5.129 |
| cholesteryl sulfate | 0.68402 | -0.54789 | 0.00011822 | 3.9273 |
| N-acetyl spermine | 0.68362 | -0.54872 | 0.0091938 | 2.0365 |
| asparagine | 0.68126 | -0.55373 | 6.92E-05 | 4.1601 |
| Urea | 0.64863 | -0.62453 | 0.00035372 | 3.4513 |
| beta-D-Fructose 6-phosphate | 0.64466 | -0.63339 | 0.00020094 | 3.6969 |
| phosphocreatine | 0.64254 | -0.63814 | 2.84E-07 | 6.5473 |
| Taurodeoxycholic acid | 0.63989 | -0.64411 | 0.0069281 | 2.1594 |
| UDP-N-acetyl-glucosamine | 0.63939 | -0.64524 | 5.38E-07 | 6.2691 |
| alanine | 0.6294 | -0.66796 | 5.55E-10 | 9.2558 |
| Ribose-5-phosphate | 0.62789 | -0.67141 | 0.012959 | 1.8874 |
| cyclic AMP | 0.62471 | -0.67874 | 0.012497 | 1.9032 |
| glycolate | 0.62003 | -0.6896 | 0.021328 | 1.671 |
| fructose-6-phosphate | 0.61157 | -0.7094 | 0.00018875 | 3.7241 |
| sarcosine | 0.59434 | -0.75063 | 1.68E-08 | 7.7757 |
| dTMP | 0.58604 | -0.77092 | 0.0022 | 2.6576 |
| lactate | 0.58451 | -0.7747 | 7.33E-10 | 9.1348 |
| D-4'-Phosphopantothenate | 0.53657 | -0.89816 | 0.0035713 | 2.4472 |
| UDP-D-glucose | 0.53179 | -0.91108 | 1.13E-07 | 6.9476 |
| ornithine | 0.52554 | -0.92814 | 0.00071277 | 3.1471 |
| N-Acetyl-D-glucosamine | 0.51148 | -0.96726 | 2.11E-08 | 7.6763 |
| spermine | 0.50482 | -0.98615 | 0.0041413 | 2.3829 |
| adenosine | 0.495 | -1.0145 | 0.001635 | 2.7865 |
| Farnesyl pyrophosphate | 0.49307 | -1.0201 | 0.0070584 | 2.1513 |
| Sedoheptulose 1,7-bisphosphate | 0.47388 | -1.0774 | 0.0028659 | 2.5427 |
| dUMP | 0.47117 | -1.0857 | 0.0006858 | 3.1638 |
| Thiamine pyrophosphate | 0.47097 | -1.0863 | 0.0056844 | 2.2453 |
| NADH | 0.46332 | -1.1099 | 0.00054766 | 3.2615 |
| S-adenosyl-L-methionine | 0.43882 | -1.1883 | 1.11E-05 | 4.9556 |
| Glycerol 3-phosphate | 0.40269 | -1.3122 | 0.00062444 | 3.2045 |
| CDP-choline | 0.39202 | -1.351 | 7.71E-06 | 5.1128 |
| histidinol | 0.38697 | -1.3697 | 0.040921 | 1.388 |
| beta-D-Fructose 1,6-bisphosphate | 0.36574 | -1.4511 | 3.79E-06 | 5.4209 |
| Glycerophosphocholine | 0.3575 | -1.484 | 2.02E-11 | 10.695 |
| dTDP | 0.35652 | -1.4879 | 0.0029723 | 2.5269 |
| 7-methylguanosine | 0.33252 | -1.5885 | 0.00011326 | 3.9459 |
| dihydroxy-acetone-phosphate | 0.32245 | -1.6328 | 2.60E-07 | 6.5855 |
| D-Glyceraldehyde 3-phosphate | 0.32024 | -1.6428 | 1.95E-06 | 5.7099 |
| inosine | 0.19127 | -2.3863 | 4.12E-08 | 7.3853 |
| Kynurenic acid | 0.11925 | -3.0679 | 0.0098739 | 2.0055 |
| thymidine | 0.026691 | -5.2275 | 1.11E-07 | 6.9541 |
| deoxyuridine | 0.01015 | -6.6223 | 3.25E-08 | 7.4875 |

**Supplementary Table 6 Dataset analyzed in this study.**

| Dataset | References |
| --- | --- |
| GSE16534 | (Lin et al., 2009) |
| GSE3141 | (Bild et al., 2006) |
| GSE8894 | (Lee et al., 2008) |

**Supplementary Table 7 MIR21 and ADSL RNA expressions in lung cancer patient tissues from a dataset GSE3141_Bild_2006.**

| EntrezID | 158 | 406991 |
| --- | --- | --- |
| Gene (converted by DAVID) | adenylosuccinate lyase(ADSL) | microRNA 21(MIR21) |
| GSM70125 | 1.366240441 | 2.393820365 |
| GSM70126 | 1.479430396 | 1.881820895 |
| GSM70127 | 0.950537128 | 2.414473078 |
| GSM70128 | 1.263181279 | 1.869417629 |
| GSM70129 | 1.04267453 | 2.471457175 |
| GSM70130 | 1.242746402 | 2.738294433 |
| GSM70131 | 1.407509339 | 2.528072571 |
| GSM70132 | 1.357589794 | 2.160070572 |
| GSM70133 | 1.150230269 | 2.275753272 |
| GSM70134 | 0.93782011 | 1.99793218 |
| GSM70135 | 1.195147418 | 2.163075386 |
| GSM70136 | 1.203879884 | 2.405351077 |
| GSM70137 | 1.75039263 | 2.069365807 |
| GSM70138 | 1.117444812 | 2.49039504 |
| GSM70139 | 1.24309621 | 2.398167156 |
| GSM70140 | 1.352516706 | 2.455572798 |
| GSM70141 | 0.995839766 | 1.994718053 |
| GSM70142 | 1.207025469 | 2.049626116 |
| GSM70143 | 1.564282515 | 1.899780103 |
| GSM70144 | 1.087267908 | 2.492940908 |
| GSM70145 | 0.897060539 | 2.814395389 |
| GSM70146 | 1.128192483 | 1.290512271 |
| GSM70147 | 1.522272933 | 2.037897834 |
| GSM70148 | 1.168582602 | 2.610978844 |
| GSM70149 | 1.384747394 | 1.676901518 |
| GSM70150 | 1.297112032 | 2.75800941 |
| GSM70151 | 0.829852594 | 2.467549862 |
| GSM70152 | 1.721748028 | 1.904010143 |
| GSM70153 | 1.222238578 | 2.28878279 |
| GSM70154 | 1.195573172 | 1.95866352 |
| GSM70155 | 0.232053289 | 2.477369887 |
| GSM70156 | 1.288953807 | 2.471337816 |
| GSM70157 | 1.525966634 | 2.15599402 |
| GSM70158 | 1.530222227 | 1.47403344 |
| GSM70159 | 1.337172294 | 2.077110092 |
| GSM70160 | 1.329840816 | 2.122988783 |
| GSM70161 | 1.256844472 | 2.430718323 |
| GSM70162 | 1.684232505 | 1.759821076 |
| GSM70163 | 0.985961376 | 2.878172362 |
| GSM70164 | 0.950483535 | 2.518211679 |
| GSM70165 | 1.235100592 | 1.993605585 |
| GSM70166 | 1.28797153 | 2.27253288 |
| GSM70167 | 1.231648366 | 2.23355991 |
| GSM70168 | 1.514820287 | 1.959600178 |
| GSM70169 | 0.931923663 | 2.739928504 |
| GSM70170 | 1.374816318 | 2.279981861 |
| GSM70171 | 1.374786431 | 2.075616742 |
| GSM70172 | 1.001977249 | 2.431830449 |
| GSM70173 | 1.078117269 | 2.348633303 |
| GSM70174 | 1.269363398 | 2.481743891 |
| GSM70175 | 1.208651212 | 2.147786829 |
| GSM70176 | 1.27210769 | 2.042536024 |
| GSM70177 | 1.372782316 | 2.2719792 |
| GSM70178 | 1.035655337 | 2.13230706 |
| GSM70179 | 1.178340188 | 2.19004059 |
| GSM70180 | 1.441023505 | 1.820335638 |
| GSM70181 | 1.126376039 | 2.100500275 |
| GSM70182 | 1.279500584 | 2.093601253 |
| GSM70183 | 0.869757759 | 2.791630286 |
| GSM70184 | 1.231271331 | 2.457532889 |
| GSM70185 | 1.372134857 | 2.219521797 |
| GSM70186 | 1.33331617 | 2.436831539 |
| GSM70187 | 1.494162638 | 2.027268359 |
| GSM70188 | 1.095335566 | 1.786854047 |
| GSM70189 | 1.361475036 | 2.062522982 |
| GSM70190 | 1.345049311 | 2.069711783 |
| GSM70191 | 1.254374247 | 2.183342408 |
| GSM70192 | 1.439071191 | 2.137615564 |
| GSM70193 | 0.981038253 | 2.830911464 |
| GSM70194 | 1.047129104 | 2.474283774 |
| GSM70195 | 1.617009063 | 1.818267882 |
| GSM70196 | 0.921567898 | 2.541213485 |
| GSM70197 | 1.12896909 | 2.572888401 |
| GSM70198 | 1.797772936 | 2.373172932 |
| GSM70199 | 1.339741574 | 2.170620412 |
| GSM70200 | 1.297084592 | 2.270918865 |
| GSM70201 | 0.991174944 | 2.478040056 |
| GSM70202 | 1.317918972 | 1.555952465 |
| GSM70203 | 1.060391969 | 2.423798563 |
| GSM70204 | 0.862337661 | 2.664115546 |
| GSM70205 | 0.989861452 | 1.957718054 |
| GSM70206 | 1.192369528 | 1.906254331 |
| GSM70207 | 1.353786253 | 2.004500805 |
| GSM70208 | 1.163801051 | 2.173788877 |
| GSM70209 | 1.183603916 | 2.562439847 |
| GSM70210 | 1.457440898 | 2.066283711 |
| GSM70211 | 1.18746813 | 1.691049542 |
| GSM70212 | 1.547539665 | 2.066371612 |
| GSM70213 | 1.208114996 | 1.795256569 |
| GSM70214 | 1.6349132 | 2.199228146 |
| GSM70215 | 1.007622033 | 1.477282191 |
| GSM70216 | 1.769107931 | 2.096378879 |
| GSM70217 | 1.652137906 | 1.972282723 |
| GSM70218 | 1.387158105 | 2.391234294 |
| GSM70219 | 1.164876969 | 2.440068652 |
| GSM70220 | 1.180083209 | 2.420447847 |
| GSM70221 | 1.686035757 | 1.910558531 |
| GSM70222 | 1.349743942 | 2.668692628 |
| GSM70223 | 1.396719042 | 2.705175859 |
| GSM70224 | 1.251959646 | 2.222344417 |
| GSM70225 | 1.465682189 | 2.561166859 |
| GSM70226 | 1.3005233 | 2.179549849 |
| GSM70227 | 1.102808606 | 2.249882295 |
| GSM70228 | 1.26300948 | 2.456972692 |
| GSM70229 | 1.234233343 | 2.690579917 |
| GSM70230 | 1.133093891 | 2.014055318 |
| GSM70231 | 1.800474498 | 1.766584287 |
| GSM70232 | 1.234415536 | 1.847883576 |
| GSM70233 | 1.451501397 | 2.306909016 |
| GSM70234 | 1.373940914 | 2.050610606 |
| GSM70235 | 1.136005613 | 2.031396991 |

**Supplementary Table 8 MIR21 and ADSL RNA expressions in lung cancer patient tissues from a dataset GSE16534_Lin_2009.**

| EntrezID | 158 | 406991 |
| --- | --- | --- |
| Gene (converted by DAVID) | adenylosuccinate lyase(ADSL) | microRNA 21(MIR21) |
| GSM415335 | 1.10212281 | 3.45219896 |
| GSM415336 | 0.99517876 | 1.501017044 |
| GSM415337 | 1.118308081 | 0.412254687 |
| GSM415338 | 1.457169327 | 0.633581266 |
| GSM415339 | 1.171542157 | 1.72199875 |
| GSM415340 | 1.013946362 | 2.357335185 |
| GSM415341 | 1.663980836 | 1.570429291 |
| GSM415342 | 0.789892721 | 1.664972389 |
| GSM415343 | 1.17388915 | 0.285953538 |
| GSM415344 | 1.1256827 | 1.649218414 |
| GSM415345 | 0.948041786 | 1.498085823 |
| GSM415346 | 1.391623453 | 1.450909362 |
| GSM415347 | 1.469512031 | 1.372207135 |
| GSM415348 | 1.603331349 | 1.623640992 |
| GSM415349 | 1.067632362 | 1.301675648 |
| GSM415350 | 1.401377767 | 2.338135691 |
| GSM415351 | 1.221972263 | 1.68607094 |
| GSM415352 | 2.047115476 | 0.589689973 |
| GSM415353 | 1.549318667 | 0.815666531 |
| GSM415354 | 1.217097638 | 0.604836851 |
| GSM415355 | 1.491125504 | -0.232413481 |
| GSM415356 | 1.638693902 | 0.914843262 |
| GSM415357 | 1.582623609 | 0.921290467 |
| GSM415358 | 1.529362337 | 0.543959442 |
| GSM415359 | 1.187263014 | 0.546070489 |
| GSM415360 | 1.506137969 | 0.621706298 |
| GSM415361 | 1.908148399 | 0.709879841 |
| GSM415362 | 1.269790468 | -0.250366488 |
| GSM415363 | 2.370876524 | -0.013424504 |
| GSM415364 | 1.126734696 | 2.745122839 |
| GSM415365 | 1.621594807 | 0.149553617 |
| GSM415366 | 1.397869431 | 0.394022162 |
| GSM415367 | 1.206860329 | 1.285266051 |
| GSM415368 | 1.546583924 | 0.416599194 |
| GSM415369 | 1.883485127 | 0.001143791 |
| GSM415370 | 1.101511663 | 1.200595859 |
| GSM415371 | 1.521170859 | 1.083363533 |
| GSM415372 | 1.43507609 | 1.386643393 |
| GSM415373 | 1.546323954 | 0.42925082 |
| GSM415374 | 0.625771068 | 0.778322421 |
| GSM415375 | 1.89573465 | -0.15412899 |
| GSM415376 | 1.816233195 | -0.406474743 |
| GSM415377 | 1.415389458 | 0.549641999 |

**Supplementary Table 9 MIR21 and ADSL RNA expressions in lung cancer patient tissues from a dataset GSE8894_Lee_2008.**

| EntrezID | 158 | 406991 |
| --- | --- | --- |
| Gene (converted by DAVID) | adenylosuccinate lyase(ADSL) | microRNA 21(MIR21) |
| GSM225465 | 1.766091776 | 2.1417777 |
| GSM225530 | 1.730123958 | 2.274436937 |
| GSM225544 | 1.973834174 | 2.390443419 |
| GSM225547 | 1.509270796 | 2.052095819 |
| GSM225552 | 1.686738099 | 2.099173769 |
| GSM225553 | 2.102889705 | 1.701791002 |
| GSM225556 | 1.327637089 | 2.170812385 |
| GSM225558 | 1.960627664 | 2.116901389 |
| GSM225563 | 2.333229068 | 1.774471129 |
| GSM225565 | 1.649976392 | 2.078399345 |
| GSM225568 | 1.970383866 | 2.084703588 |
| GSM225572 | 1.434620293 | 2.544314768 |
| GSM225574 | 1.474652473 | 2.391693068 |
| GSM225575 | 1.982179584 | 2.197674419 |
| GSM225578 | 1.919708878 | 1.717863576 |
| GSM225582 | 1.958796771 | 2.184715892 |
| GSM225584 | 1.803412277 | 2.864201078 |
| GSM225585 | 1.204027137 | 1.661496174 |
| GSM225586 | 1.929436474 | 2.907866857 |
| GSM225587 | 1.741236885 | 3.00578309 |
| GSM225589 | 1.314191904 | 2.269307628 |
| GSM225590 | 2.369341059 | 2.471769292 |
| GSM225591 | 1.767649971 | 2.379409058 |
| GSM225593 | 1.768264452 | 2.322654235 |
| GSM225594 | 1.64490151 | 2.317818842 |
| GSM225595 | 1.712513573 | 2.376989054 |
| GSM225596 | 2.002795497 | 2.360710614 |
| GSM225598 | 1.533387467 | 2.359739714 |
| GSM225599 | 1.637724257 | 2.558876213 |
| GSM225601 | 1.427785524 | 1.850978922 |
| GSM225603 | 2.092006166 | 2.276979191 |
| GSM225604 | 1.884208463 | 2.142673773 |
| GSM225765 | 2.124339943 | 1.653656642 |
| GSM225766 | 1.73902887 | 2.184634576 |
| GSM225767 | 1.701930737 | 2.536852145 |
| GSM225768 | 1.873259736 | 2.233440039 |
| GSM225769 | 1.669806282 | 2.38099675 |
| GSM225770 | 1.934707847 | 1.824648347 |
| GSM225771 | 1.995797299 | 2.522477678 |
| GSM225772 | 1.839587658 | 2.341963423 |
| GSM225773 | 1.78848835 | 2.655228959 |
| GSM225774 | 2.139224565 | 2.200925051 |
| GSM225775 | 2.093587713 | 2.151496936 |
| GSM225776 | 1.7435444 | 2.202233782 |
| GSM225777 | 1.969583593 | 1.876186231 |
| GSM225778 | 2.010936419 | 2.017302629 |
| GSM225779 | 1.808678085 | 2.329358005 |
| GSM225780 | 1.779701261 | 1.83815428 |
| GSM225781 | 2.139095641 | 2.152584933 |
| GSM225782 | 1.909662257 | 2.017379387 |
| GSM225783 | 1.899539139 | 1.919378153 |
| GSM225784 | 1.681755218 | 2.558783477 |
| GSM225785 | 2.132868801 | 2.198255615 |
| GSM225786 | 2.05440988 | 1.727578197 |
| GSM225787 | 1.737951383 | 2.196802114 |
| GSM225788 | 1.91514216 | 2.344718829 |
| GSM225789 | 2.125000849 | 2.018099286 |
| GSM225790 | 1.625292948 | 2.228240293 |
| GSM225791 | 2.227162378 | 1.695011547 |
| GSM225792 | 2.281584812 | 2.279391226 |
| GSM225793 | 2.173965801 | 2.452350199 |
| GSM225794 | 1.706686198 | 2.34836845 |
| GSM225795 | 1.957039148 | 2.079128791 |
| GSM225796 | 1.525243453 | 2.425312605 |
| GSM225797 | 1.59139704 | 1.701286907 |
| GSM225798 | 1.529333621 | 2.001138698 |
| GSM225799 | 1.549827065 | 2.481973919 |
| GSM225800 | 1.522491573 | 1.951039936 |
| GSM225801 | 2.059524636 | 2.174030201 |
| GSM225802 | 1.516474412 | 2.676389606 |
| GSM225803 | 1.677357744 | 2.761123148 |
| GSM225804 | 1.703110336 | 2.003296917 |
| GSM225805 | 1.657150045 | 2.457876486 |
| GSM225806 | 1.547488347 | 2.713083564 |
| GSM225807 | 1.524575517 | 2.033049731 |
| GSM225808 | 1.491085491 | 2.518802263 |
| GSM225809 | 2.112860277 | 2.304489849 |
| GSM225810 | 1.79417409 | 2.567019045 |
| GSM225811 | 1.265633035 | 2.720804635 |
| GSM225812 | 1.487212801 | 2.124115066 |
| GSM225813 | 1.883549405 | 2.297788607 |
| GSM225814 | 1.329124635 | 2.441797445 |
| GSM225815 | 1.501276897 | 2.923638059 |
| GSM225816 | 2.115835301 | 1.544511075 |
| GSM225817 | 1.731650552 | 2.26523959 |
| GSM225818 | 1.794557444 | 2.201549453 |
| GSM225819 | 2.299930586 | 2.368938399 |
| GSM225820 | 2.251059312 | 2.208801422 |
| GSM225821 | 1.933968219 | 2.657211965 |
| GSM225823 | 1.610755909 | 2.383432866 |
| GSM225824 | 2.343591188 | 2.657486544 |
| GSM225825 | 2.074964639 | 2.109296621 |
| GSM225827 | 1.633995735 | 2.342021135 |
| GSM225828 | 2.363129317 | 2.155715732 |
| GSM225829 | 1.672682335 | 1.766078045 |
| GSM225831 | 1.963632167 | 2.39685374 |
| GSM225832 | 2.241319691 | 2.416776923 |
| GSM225833 | 1.86048785 | 2.543412568 |
| GSM225835 | 1.750068196 | 3.004534624 |
| GSM225836 | 1.737393545 | 2.384049814 |
| GSM225844 | 2.005285124 | 1.981561231 |
| GSM225846 | 2.162002015 | 1.738953592 |
| GSM225848 | 1.718309031 | 2.389020943 |
| GSM225849 | 1.789122902 | 2.172888203 |
| GSM225851 | 2.025418757 | 2.100466472 |
| GSM225852 | 2.252215439 | 2.254675812 |
| GSM225854 | 2.153103907 | 2.34106915 |
| GSM225855 | 1.750071174 | 2.625627617 |
| GSM225857 | 2.051033945 | 1.806608903 |
| GSM225859 | 1.889325503 | 2.049304971 |
| GSM225861 | 1.970393916 | 2.390409042 |
| GSM225863 | 1.715863965 | 2.504606156 |
| GSM225864 | 1.463908256 | 2.296107531 |
| GSM225866 | 1.860052024 | 2.532410688 |
| GSM225868 | 1.782244723 | 1.970625909 |
| GSM225873 | 2.091325488 | 2.298050357 |
| GSM225874 | 1.324924174 | 2.470212948 |
| GSM225876 | 1.896398549 | 2.196285414 |
| GSM225877 | 2.112054473 | 2.343617565 |
| GSM225879 | 2.362255496 | 1.807098635 |
| GSM225880 | 1.912775612 | 2.321361387 |
| GSM225882 | 1.421983422 | 2.584062084 |
| GSM225883 | 1.62043889 | 2.345561145 |
| GSM225884 | 1.47739354 | 2.525347202 |
| GSM225886 | 2.458326247 | 1.983212676 |
| GSM225888 | 2.208510326 | 2.037031244 |
| GSM225889 | 2.18821294 | 1.85262502 |
| GSM225890 | 2.083270453 | 1.968727774 |
| GSM225893 | 2.029131144 | 2.175736373 |
| GSM225894 | 1.560603069 | 2.430208763 |
| GSM225895 | 1.961677619 | 2.157640328 |
| GSM225896 | 1.689158095 | 2.422278836 |
| GSM225897 | 1.908999669 | 2.377575974 |
| GSM225898 | 1.499971909 | 2.03369658 |
| GSM225899 | 1.546025103 | 2.548182499 |
| GSM225900 | 1.351830538 | 1.623666553 |
| GSM225901 | 1.342923184 | 2.605150988 |
| GSM225902 | 1.840169997 | 2.201138378 |

**Supplementary Table 10:** **A list of top upregulated and downregulated metabolites in lung tissues of MIR21 knockout mice compared with wild-type mice (n=137)**.

| **Metabolites** | **FC (*MIR21* KO/WT))** | **log2(FC)** | ***P* value** | **-log10(*P*)** |
| --- | --- | --- | --- | --- |
| N-acetyl spermine | 4.0873 | 2.0311 | 0.002515 | 2.5995 |
| trehalose-sucrose | 3.753 | 1.908 | 0.000127 | 3.8964 |
| UDP-D-glucuronate | 3.35 | 1.7442 | 2.04E-06 | 5.6903 |
| UTP | 3.3208 | 1.7315 | 1.14E-05 | 4.9438 |
| glutathione disulfide | 3.2733 | 1.7108 | 0.003169 | 2.4991 |
| NADH | 3.13 | 1.6462 | 0.023112 | 1.6362 |
| propionyl-CoA | 2.8378 | 1.5048 | 1.38E-05 | 4.8593 |
| dTMP | 2.7682 | 1.4689 | 6.88E-06 | 5.1621 |
| fructose-1,6-bisphosphate | 2.7309 | 1.4494 | 0.000402 | 3.3954 |
| p-aminobenzoate | 2.7274 | 1.4475 | 0.002385 | 2.6226 |
| ATP | 2.642 | 1.4016 | 3.88E-05 | 4.4109 |
| hypoxanthine | 2.6206 | 1.3899 | 0.001747 | 2.7576 |
| dGTP | 2.5901 | 1.373 | 1.66E-05 | 4.7805 |
| malonyl-CoA | 2.4799 | 1.3103 | 0.021873 | 1.6601 |
| HMG-CoA | 2.4655 | 1.3019 | 0.010301 | 1.9871 |
| acetyl-CoA | 2.4435 | 1.2889 | 0.002601 | 2.5849 |
| SBP | 2.4326 | 1.2825 | 0.015451 | 1.811 |
| cysteine sulfinate | 2.4306 | 1.2813 | 0.000753 | 3.1233 |
| GTP | 2.3818 | 1.252 | 0.000455 | 3.3421 |
| UDP-D-glucose | 2.3301 | 1.2204 | 1.30E-06 | 5.8864 |
| shikimate | 2.2896 | 1.1951 | 0.005943 | 2.226 |
| S-methyl-5-thioadenosine | 2.2676 | 1.1811 | 0.000757 | 3.1211 |
| dimethylglycine | 2.1719 | 1.1189 | 0.00015 | 3.8235 |
| Kynurenine | 2.1649 | 1.1143 | 0.007602 | 2.1191 |
| UDP-N-acetyl-glucosamine | 2.155 | 1.1077 | 0.003701 | 2.4317 |
| anthranilate | 2.1537 | 1.1068 | 0.017532 | 1.7562 |
| Phosphorylcholine | 2.1418 | 1.0988 | 0.006997 | 2.1551 |
| biotin | 2.127 | 1.0888 | 0.013559 | 1.8678 |
| NAD | 2.1159 | 1.0813 | 0.00073 | 3.1368 |
| UDP | 2.0895 | 1.0631 | 0.000697 | 3.157 |
| Acetylcarnitine DL | 2.0692 | 1.0491 | 8.70E-05 | 4.0606 |
| CDP-ethanolamine | 2.0556 | 1.0395 | 0.000318 | 3.4977 |
| choline | 2.0294 | 1.021 | 0.000235 | 3.6282 |
| homocysteic acid | 2.0205 | 1.0147 | 0.032191 | 1.4923 |
| 2-Aminooctanoic acid | 2.0051 | 1.0037 | 0.029332 | 1.5327 |
| 2,3-Diphosphoglyceric acid | 2.0014 | 1.001 | 3.09E-05 | 4.5102 |
| uridine | 1.9885 | 0.99167 | 0.000457 | 3.3397 |
| Glycerophosphocholine | 1.9875 | 0.99095 | 1.26E-06 | 5.8988 |
| 1,3-diphopshateglycerate | 1.9481 | 0.96204 | 2.63E-05 | 4.5805 |
| 5-phosphoribosyl-1-pyrophosphate | 1.9263 | 0.9458 | 0.027013 | 1.5684 |
| carnitine | 1.926 | 0.9456 | 0.003885 | 2.4107 |
| Creatinine | 1.8957 | 0.9227 | 0.000146 | 3.8346 |
| dihydroxy-acetone-phosphate | 1.8679 | 0.90142 | 0.008033 | 2.0951 |
| dAMP | 1.8578 | 0.89361 | 0.000147 | 3.8317 |
| OBP | 1.8332 | 0.8744 | 0.001683 | 2.7739 |
| CTP | 1.8283 | 0.87053 | 0.000564 | 3.2484 |
| D-gluconate | 1.8204 | 0.86429 | 0.000111 | 3.9548 |
| S-adenosyl-L-methionine | 1.8156 | 0.86042 | 0.001662 | 2.7794 |
| succinyl-CoA | 1.8046 | 0.85164 | 0.003404 | 2.468 |
| phosphoenolpyruvate | 1.8026 | 0.85011 | 4.80E-05 | 4.3192 |
| D-glyceraldehdye-3-phosphate | 1.8016 | 0.84931 | 0.018339 | 1.7366 |
| citrulline | 1.7968 | 0.84544 | 0.03816 | 1.4184 |
| phosphocreatine | 1.7942 | 0.84332 | 0.007174 | 2.1442 |
| betaine aldehyde | 1.7904 | 0.84029 | 0.001952 | 2.7094 |
| UMP | 1.7441 | 0.80245 | 0.00074 | 3.1309 |
| ethanolamine | 1.7412 | 0.80011 | 0.009135 | 2.0393 |
| cholesterol | 1.6441 | 0.71731 | 0.031572 | 1.5007 |
| orotidine-5-phosphate | 1.6307 | 0.70551 | 0.014948 | 1.8254 |
| S-adenosyl-L-homoCysteine | 1.6129 | 0.6897 | 3.58E-05 | 4.4466 |
| Thiamine pyrophosphate | 1.6082 | 0.68543 | 0.002618 | 2.582 |
| guanosine | 1.5975 | 0.67582 | 0.001637 | 2.7859 |
| inosine | 1.59 | 0.66907 | 0.002189 | 2.6599 |
| D-glucono--lactone-6-phosphate | 1.5893 | 0.6684 | 0.033264 | 1.478 |
| dGDP | 1.584 | 0.66356 | 0.001044 | 2.9813 |
| betaine | 1.5836 | 0.66317 | 9.64E-05 | 4.016 |
| GDP | 1.5675 | 0.64844 | 0.001667 | 2.7781 |
| Indole-3-carboxylic acid | 1.5632 | 0.64448 | 0.045299 | 1.3439 |
| IDP | 1.5573 | 0.63901 | 0.000916 | 3.038 |
| myo-inositol | 1.548 | 0.6304 | 0.001657 | 2.7806 |
| ADP | 1.5458 | 0.62839 | 0.00177 | 2.7521 |
| Carbamoyl phosphate | 1.545 | 0.62764 | 0.009592 | 2.0181 |
| guanine | 1.5442 | 0.6269 | 0.001216 | 2.9149 |
| homocysteine | 1.5224 | 0.60639 | 0.010853 | 1.9644 |
| N-Acetyl-L-alanine1 | 1.5173 | 0.60147 | 0.001759 | 2.7548 |
| cytidine | 1.5098 | 0.59431 | 0.009524 | 2.0212 |
| 4-Pyridoxic acid | 1.4975 | 0.58259 | 0.02272 | 1.6436 |
| N6-Acetyl-L-lysine | 1.4871 | 0.57248 | 0.000239 | 3.6218 |
| aspartate | 1.4861 | 0.57148 | 0.014411 | 1.8413 |
| hydroxyphenylpyruvate | 1.486 | 0.5714 | 0.034982 | 1.4562 |
| Acetyllysine | 1.4752 | 0.56091 | 0.000301 | 3.5219 |
| ribose-phosphate | 1.4659 | 0.55182 | 0.001771 | 2.7518 |
| sn-glycerol-3-phosphate | 1.4643 | 0.55024 | 0.000458 | 3.3392 |
| FAD | 1.4476 | 0.53367 | 0.001035 | 2.985 |
| 6-phospho-D-gluconate | 1.4392 | 0.52531 | 0.002321 | 2.6344 |
| cyclic-AMP | 1.4344 | 0.52043 | 0.029327 | 1.5327 |
| adenine | 1.4277 | 0.51365 | 0.004128 | 2.3843 |
| AICAR (aminoimidazole carboxamide ribonucleotide) | 1.4236 | 0.50953 | 0.012955 | 1.8876 |
| xanthine | 1.4094 | 0.49511 | 0.008882 | 2.0515 |
| DL-Pipecolic acid | 1.4028 | 0.48826 | 0.006509 | 2.1865 |
| adenosine 5-phosphosulfate | 1.3907 | 0.47586 | 0.013439 | 1.8716 |
| IMP | 1.3819 | 0.46661 | 0.019208 | 1.7165 |
| glutamine | 1.3816 | 0.46631 | 0.004907 | 2.3092 |
| acetylphosphate | 1.3751 | 0.45959 | 0.000545 | 3.2638 |
| fructose-6-phosphate | 1.3703 | 0.45448 | 0.017093 | 1.7672 |
| Pyrophosphate | 1.3653 | 0.44919 | 0.00474 | 2.3242 |
| glutathione | 1.3564 | 0.43976 | 0.006712 | 2.1731 |
| a-ketoglutarate | 1.3427 | 0.42514 | 0.018585 | 1.7308 |
| N-acetyl-glucosamine-1-phosphate | 1.3262 | 0.40731 | 0.000828 | 3.0822 |
| taurine | 1.3106 | 0.39021 | 0.005608 | 2.2512 |
| cholesteryl sulfate | 1.2978 | 0.37602 | 0.036065 | 1.4429 |
| glucose-6-phosphate | 1.2922 | 0.36979 | 0.029065 | 1.5366 |
| Phenylpropiolic acid | 1.2821 | 0.3585 | 0.0243 | 1.6144 |
| D-erythrose-4-phosphate | 1.2743 | 0.34972 | 0.01095 | 1.9606 |
| D-sedoheptulose-1-7-phosphate | 1.2614 | 0.33497 | 0.009715 | 2.0126 |
| hexose-phosphate | 1.2419 | 0.31251 | 0.018948 | 1.7224 |
| O8P-O1P | 1.2332 | 0.30237 | 0.038243 | 1.4174 |
| 2-Hydroxy-2-methylbutanedioic acid | 1.2229 | 0.29036 | 0.005471 | 2.262 |
| 3-phosphoglycerate | 1.2058 | 0.26995 | 0.001175 | 2.9299 |
| 2-keto-isovalerate | 1.2054 | 0.26947 | 0.001969 | 2.7058 |
| malate | 1.1899 | 0.25087 | 0.046907 | 1.3288 |
| 2-hydroxygluterate | 1.1467 | 0.19751 | 0.045176 | 1.3451 |
| Pyroglutamic acid | 0.87984 | -0.18469 | 0.011333 | 1.9457 |
| arginosuccinic acid | 0.87606 | -0.1909 | 0.0099 | 2.0044 |
| Phenyllactic acid | 0.86731 | -0.20539 | 0.0473 | 1.3251 |
| valine | 0.86664 | -0.2065 | 0.027898 | 1.5544 |
| histidine | 0.85774 | -0.22139 | 0.0265 | 1.5768 |
| methionine | 0.85757 | -0.22167 | 1.69E-05 | 4.7711 |
| arginine | 0.8555 | -0.22517 | 0.012651 | 1.8979 |
| L-arginino-succinate | 0.85016 | -0.23419 | 0.000901 | 3.0452 |
| serine | 0.84487 | -0.2432 | 0.014198 | 1.8478 |
| Methylcysteine | 0.82692 | -0.27418 | 0.011432 | 1.9419 |
| riboflavin | 0.7994 | -0.32301 | 0.007532 | 2.1231 |
| tyrosine | 0.79652 | -0.32822 | 0.004823 | 2.3167 |
| 1-Methyl-Histidine | 0.76569 | -0.38516 | 0.001071 | 2.9703 |
| folate | 0.74501 | -0.42467 | 0.043766 | 1.3589 |
| orotate | 0.67426 | -0.56861 | 0.036933 | 1.4326 |
| Methylmalonic acid | 0.66861 | -0.58077 | 0.009724 | 2.0122 |
| retinoic acid | 0.58379 | -0.77648 | 0.0054 | 2.2676 |
| succinate | 0.57774 | -0.7915 | 0.000694 | 3.1585 |
| pyridoxine | 0.55144 | -0.85872 | 1.12E-06 | 5.9507 |
| adenosine | 0.53514 | -0.90201 | 0.002119 | 2.6739 |
| deoxyguanosine | 0.50513 | -0.98528 | 0.029113 | 1.5359 |
| 1-Methyladenosine | 0.49868 | -1.0038 | 0.01658 | 1.7804 |
| cysteine | 0.48245 | -1.0515 | 7.89E-05 | 4.1029 |
| Cystine | 0.31273 | -1.677 | 0.002137 | 2.6702 |
| Ascorbic acid | 0.15568 | -2.6834 | 0.001156 | 2.9372 |
| 2-Isopropylmalic acid | 0.12929 | -2.9513 | 0.007116 | 2.1478 |

**Abbreviations**

| 3p | Passenger strand |
| --- | --- |
| 5p | Guide strand |
| ADSL | Adenylosuccinate lyase |
| ADSS | Adenylosuccinate synthase |
| AICAR | 5-aminoimidazole-4-carboxamide riboside; acadesine |
| AKT | AKT serine/threonine kinase |
| AMP | Adenosine monophosphate |
| AMPD2 | Adenosine monophosphate deaminase 2 |
| AMPK | AMP-activated protein kinase |
| APBADP | Adenosine phosphonobutyric acid, 2′(3′), 5′-diphosphate |
| ATIC | Phosphoribosyl aminoimidazole carboxamide formyltransferase, AICAR transformylase |
| ATP | Adenosine triphosphate |
| CoA | Coenzyme A |
| COSMIC | Catalogue Of Somatic Mutations In Cancer |
| DICER1 | Dicer 1, ribonuclease III |
| DTP | Drug-tolerant persister |
| DTPCs | Drug-tolerant persister cells |
| EGFR | Epidermal growth factor receptor |
| ERK | Extracellular signal-regulated kinase |
| GART | Phosphoribosylglycinamide formyltransferase |
| GDP | Guanosine diphosphate |
| GFP | Green fluorescence protein |
| GSH | Glutathione |
| GSSG | Glutathione disulfide |
| H2O2 | Hydrogen peroxide |
| IDP | Inosine diphosphate |
| IMP | Inosine monophosphate |
| KO | Knock-out |
| LC-MS | Liquid chromatography-mass spectrometry |
| LNA | Locked nucleic acid |
| miRNA-seq | microRNA sequencing |
| mRNAs | Messenger RNAs |
| mTORC1 | Mechanistic target of rapamycin complex 1 |
| MTHFD2 | Methylenetetrahydrofolate dehydrogenase (NADP+ dependent) 2 |
| ncRNAs | Non-coding RNAs |
| NRF2 | Nuclear factor erythroid 2–related factor 2 |
| OTR | Osimertinib-tolerant |
| PAICS | Phosphoribosylaminoimidazole carboxylase |
| PDCD4 | Programmed cell death 4 |
| PFAS | Phosphoribosylformylglycinamidine synthase |
| PI3K | Phosphoinositide 3-kinase |
| pre-miRNA | Precursor miRNA |
| PPAT | Phosphoribosyl pyrophosphate amidotransferase |
| PRPP | Phosphoribosyl pyrophosphate |
| PTEN | Phosphatase and tensin homolog |
| ROS | Reactive oxygen species |
| RNAs | Ribonucleic acids |
| SAH | S-adenosylhomocysteine |
| SAICAR | Succinylaminoimidazole carboxamide ribonucleotide; succino-AICAR |
| SAM | S-adenosyl-L-methionine |
| SDH | Succinate dehydrogenase |
| SRM | Selected reaction monitoring |
| S-AMP | Adenylosuccinate; succinaly-AMP |
| TCA | Tricarboxylic acid |
| UTR | Untranslated region |
