# Supplementary material for: MicroRNA-21 guide and passenger strand regulation of adenylosuccinate lyase-mediated purine metabolism promotes transition to an EGFR-TKI-tolerant persister state
Source: Cancer Gene Ther. 2022 Jul 15;29(12):1878–94. doi: 10.1038/s41417-022-00504-y (PMC9750876; doi:10.1038/s41417-022-00504-y)
Supplement: Supplementary file 18 — Related Manuscript File-original data files [file 41417_2022_504_MOESM18_ESM.docx]

**Original data files**

1. **Original CT values in qPCR data**

Figure 1D – left panel

| **Sample** | **Detector** | Ct 1 | Ct 2 | Ct 3 |
| --- | --- | --- | --- | --- |
| H1650 | miR-21-5p | 21.52 | 21.49 | 21.51 |
| HCC827 | miR-21-5p | 18.6 | 18.6 | 18.59 |
| H3255 | miR-21-5p | 17.85 | 17.84 | 17.83 |
| PC9 | miR-21-5p | 20.85 | 20.84 | 20.85 |
| H1975 | miR-21-5p | 18.28 | 18.25 | 18.21 |
| AALE | miR-21-5p | 19.95 | 19.92 | 19.96 |
| H1650 | miR-423-5p (endogenous control) | 24.85 | 24.94 | 24.89 |
| HCC827 | miR-423-5p (endogenous control) | 23.93 | 23.95 | 23.95 |
| H3255 | miR-423-5p (endogenous control) | 22.87 | 22.84 | 22.8 |
| PC9 | miR-423-5p (endogenous control) | 23.97 | 23.99 | 23.97 |
| H1975 | miR-423-5p (endogenous control) | 24.64 | 24.62 | 24.61 |
| AALE | miR-423-5p (endogenous control) | 22.68 | 22.66 | 22.59 |

Figure 1D – right panel

| **Sample** | **Detector** | Ct 1 | Ct 2 | Ct 3 |
| --- | --- | --- | --- | --- |
| H1650 | miR-21-3p | 27.77 | 27.7 |  |
| HCC827 | miR-21-3p | 23.21 | 23.25 |  |
| H3255 | miR-21-3p | 23.18 | 23.16 |  |
| PC9 | miR-21-3p | 22.2 | 22.16 |  |
| H1975 | miR-21-3p | 21.58 | 21.46 |  |
| AALE | miR-21-3p | 22.83 | 22.74 |  |
| H1650 | miR-423-5p (endogenous control) | 25.32 | 25.56 |  |
| HCC827 | miR-423-5p (endogenous control) | 23.78 | 23.74 |  |
| H3255 | miR-423-5p (endogenous control) | 23.03 | 22.98 |  |
| PC9 | miR-423-5p (endogenous control) | 22.11 | 22.12 |  |
| H1975 | miR-423-5p (endogenous control) | 23.45 | 23.5 |  |
| AALE | miR-423-5p (endogenous control) | 21.05 | 21.16 |  |

Figure 1E

| **Sample** | **Detector** | Ct 1 | Ct 2 | Ct 3 |
| --- | --- | --- | --- | --- |
| H1975 Parental | miR-21-5p | 16.087 | 16.082 | 16.000 |
| H1975 OTR | miR-21-5p | 14.739 | 14.615 | 14.581 |
| H1975 Parental | miR-21-3p | 22.946 | 22.891 | 22.850 |
| H1975 OTR | miR-21-3p | 22.506 | 22.409 | 22.312 |
| H1975 Parental | miR-423-5p (endogenous control) | 22.823 | 22.850 | 22.855 |
| H1975 OTR | miR-423-5p (endogenous control) | 23.306 | 23.053 | 22.807 |

Figure 1F

| **Sample** | **Detector** | Ct 1 | Ct 2 | Ct 3 |
| --- | --- | --- | --- | --- |
| H1975 Lenti Anti-miR-control | miR-21-5p | 17.422 | 17.442 | 17.438 |
| H1975 Lenti Anti-miR-21-5p | miR-21-5p | 19.463 | 19.271 | 19.180 |
| H1975 Lenti Anti-miR-control | miR-21-3p | 21.978 | 21.999 | 22.041 |
| H1975 Lenti Anti-miR-21-5p | miR-21-3p | 24.262 | 23.804 | 23.771 |
| H1975 Lenti Anti-miR-control | miR-423-5p (endogenous control) | 23.647 | 23.883 | 23.920 |
| H1975 Lenti Anti-miR-21-5p | miR-423-5p (endogenous control) | 25.345 | 24.810 | 24.885 |

Figure 1G

| **Sample** | **Detector** | Ct 1 | Ct 2 | Ct 3 |
| --- | --- | --- | --- | --- |
| H1975 LNA Anti-miR-control | miR-21-5p | 29.338 | 28.793 | 28.978 |
| H1975 LNA Anti-miR-21-5p | miR-21-5p |  | 40.000 | 39.900 |
| H1975 LNA Anti-miR-control | miR-21-3p | 32.986 | 33.115 | 32.927 |
| H1975 LNA Anti-miR-21-5p | miR-21-3p |  | 40.000 | 39.900 |
| H1975 LNA Anti-miR-control | miR-423-5p (endogenous control) | 28.491 | 28.335 | 28.247 |
| H1975 LNA Anti-miR-21-5p | miR-423-5p (endogenous control) | 34.110 | 34.008 | 34.005 |

Figure 1H

| **Sample** | **Detector** | Ct 1 | Ct 2 | Ct 3 |
| --- | --- | --- | --- | --- |
| H1975 LNA Anti-miR-control | miR-21-3p | 23.890 | 23.925 | 23.869 |
| H1975 LNA Anti-miR-21-3p | miR-21-3p | 29.535 | 29.316 | 29.389 |
| H1975 LNA Anti-miR-control | miR-21-5p | 20.827 | 20.674 | 20.534 |
| H1975 LNA Anti-miR-21-3p | miR-21-5p | 20.347 | 18.989 | 18.908 |
| H1975 LNA Anti-miR-control | miR-186-5p (endogenous control) | 25.869 | 25.967 | 26.418 |
| H1975 LNA Anti-miR-21-3p | miR-186-5p (endogenous control) | 25.803 | 26.002 | 25.952 |

Figure 1K

| **Sample** | **Detector** | Ct 1 | Ct 2 | Ct 3 |
| --- | --- | --- | --- | --- |
| HCC827 Scrambled | miR-21-5p | 22.095 | 22.212 | 22.029 |
| HCC827 miR-21-5p OE | miR-21-5p | 21.836 | 21.686 | 21.714 |
| HCC827 Scrambled | miR-21-3p | 26.718 | 26.562 | 26.432 |
| HCC827 miR-21-5p OE | miR-21-3p | 26.262 | 26.241 | 26.151 |
| HCC827 Scrambled | miR-423-5p (endogenous control) | 27.935 | 27.791 | 27.737 |
| HCC827 miR-21-5p OE | miR-423-5p (endogenous control) | 27.878 | 27.780 | 27.846 |

Figure 1N

| **Sample** | **Detector** | Ct 1 | Ct 2 | Ct 3 |
| --- | --- | --- | --- | --- |
| miRNA NC 40nM | miR-21-3p | 24.662 | 24.662 | 24.662 |
| miR-21-3p mimic 40nM | miR-21-3p | 17.637 | 17.637 | 17.637 |
| miR-21-5p mimic 40nM | miR-21-3p | 25.889 | 25.889 | 25.889 |
| miRNA NC 40nM | miR-21-5p | 20.561 | 20.561 | 20.561 |
| miR-21-3p mimic 40nM | miR-21-5p | 21.103 | 21.103 | 21.103 |
| miR-21-5p mimic 40nM | miR-21-5p | 20.166 | 20.166 | 20.166 |
| miRNA NC 40nM | miR-423-5p (endogenous control) | 26.522 | 26.522 | 26.522 |
| miR-21-3p mimic 40nM | miR-423-5p (endogenous control) | 27.309 | 27.309 | 27.309 |
| miR-21-5p mimic 40nM | miR-423-5p (endogenous control) | 27.880 | 27.880 | 27.880 |

Supplementary Figure 3

| **Sample** | **Detector** | Ct 1 | Ct 2 | Ct 3 |
| --- | --- | --- | --- | --- |
| LNA Anti-miR-control H1975 | Pre-miR-21 | 29.947 | 29.947 | 29.947 |
| LNA Anti-miR-21-5p H1975 | Pre-miR-21 | 30.892 | 30.892 | 30.892 |
| LNA Anti-miR-control H1975 | GAPDH (endogenous control) | 25.021 | 25.021 | 25.021 |
| LNA Anti-miR-21-5p H1975 | GAPDH (endogenous control) | 27.720 | 27.720 | 27.720 |

Supplementary Figure 15A

| **Sample** | **Detector** | Ct 1 | Ct 2 | Ct 3 |
| --- | --- | --- | --- | --- |
| H1975 Parental H2O2 0.5mM | miR-21-5p | 20.219 | 20.124 | 20.121 |
| H1975 Parental H2O2 1mM | miR-21-5p | 19.461 | 19.443 | 19.316 |
| H1975 Parental H2O2 0mM | miR-21-5p | 22.385 | 22.496 | 22.427 |
| H1975 Parental H2O2 0.5mM | miR-21-3p | 26.369 | 26.251 | 26.512 |
| H1975 Parental H2O2 1mM | miR-21-3p | 26.041 | 26.024 | 25.993 |
| H1975 Parental H2O2 0mM | miR-21-3p | 28.526 | 28.635 | 28.647 |
| H1975 Parental H2O2 0.5mM | miR-423-5p (endogenous control) | 26.479 | 26.452 | 26.365 |
| H1975 Parental H2O2 1mM | miR-423-5p (endogenous control) | 25.268 | 25.115 | 25.078 |
| H1975 Parental H2O2 0mM | miR-423-5p (endogenous control) | 27.691 | 27.525 | 27.498 |

Supplementary Figure 15B

| **Sample** | **Detector** | Ct 1 | Ct 2 | Ct 3 |
| --- | --- | --- | --- | --- |
| H1975 OTR H2O2 0.5 mM | miR-21-5p | 19.662 | 19.510 | 19.397 |
| H1975 OTR H2O2 1mM | miR-21-5p | 19.299 | 19.156 | 19.116 |
| H1975 OTR H2O2 0mM | miR-21-5p | 26.895 | 26.818 | 26.838 |
| H1975 OTR H2O2 0.5 mM | miR-21-3p | 26.529 | 26.456 | 26.476 |
| H1975 OTR H2O2 1mM | miR-21-3p | 26.346 | 26.274 | 26.273 |
| H1975 OTR H2O2 0mM | miR-21-3p | 33.104 | 32.181 | 32.544 |
| H1975 OTR H2O2 0.5 mM | miR-423-5p (endogenous control) | 25.591 | 25.433 | 25.500 |
| H1975 OTR H2O2 1mM | miR-423-5p (endogenous control) | 25.171 | 24.996 | 24.892 |
| H1975 OTR H2O2 0mM | miR-423-5p (endogenous control) | 31.428 | 31.525 | 31.340 |

Supplementary Figure 15C

| **Sample** | **Detector** | Ct 1 | Ct 2 | Ct 3 |
| --- | --- | --- | --- | --- |
| H1975 Parental AICAR 0.1mM | miR-21-5p | 20.709 | 20.555 | 20.476 |
| H1975 Parental AICAR 1mM | miR-21-5p | 22.441 | 22.197 | 22.202 |
| H1975 Parental AICAR 0mM | miR-21-5p | 20.506 | 20.201 | 20.070 |
| H1975 Parental AICAR 0.1mM | miR-21-3p | 27.614 | 27.240 | 27.019 |
| H1975 Parental AICAR 1mM | miR-21-3p | 30.101 | 29.660 | 29.594 |
| H1975 Parental AICAR 0mM | miR-21-3p | 27.794 | 27.606 | 27.100 |
| H1975 Parental AICAR 0.1mM | miR-423-5p (endogenous control) | 27.346 | 26.670 | 26.452 |
| H1975 Parental AICAR 1mM | miR-423-5p (endogenous control) | 29.012 | 28.441 | 28.240 |
| H1975 Parental AICAR 0mM | miR-423-5p (endogenous control) | 27.227 | 26.830 | 26.613 |

Supplementary Figure 15D

| **Sample** | **Detector** | Ct 1 | Ct 2 | Ct 3 |
| --- | --- | --- | --- | --- |
| H1975 OTR AICAR 0.1mM | miR-21-5p |  | 25.631 | 24.250 |
| H1975 OTR AICAR 1mM | miR-21-5p | 20.662 | 20.145 | 20.007 |
| H1975 OTR AICAR 0mM | miR-21-5p | 25.771 | 24.938 | 24.714 |
| H1975 OTR AICAR 0.1mM | miR-21-3p |  | 34.827 | 32.823 |
| H1975 OTR AICAR 1mM | miR-21-3p | 29.177 | 28.775 | 28.542 |
| H1975 OTR AICAR 0mM | miR-21-3p |  | 33.140 | 31.427 |
| H1975 OTR AICAR 0.1mM | miR-423-5p (endogenous control) | 31.657 | 30.260 | 30.476 |
| H1975 OTR AICAR 1mM | miR-423-5p (endogenous control) | 27.777 | 27.404 | 27.401 |
| H1975 OTR AICAR 0mM | miR-423-5p (endogenous control) | 31.451 | 29.988 | 29.385 |

2. **Un-cropped images of the western blots**

 **Supplementary Figure 6.** Un-cropped images of the western blots for ADSL in untreated H1975 parental and osimertinib-tolerant (OTR) cells (A), OTR cells treated with LNA miR-21-5p inhibitor (C), or parental cells treated with LNA inhibitors against a scrambled control, miR-21-3p or miR-21-5p (E). β-Actin was used as a loading control (B, D, F).

 **Supplementary Figure 7.** Un-cropped images of the western blots for PPAT (A), GART (B), PFAS (C), PAICS (D), ATIC (E), AMPD2 (F), and ADSS (G) in in H1975 parental and osimertinib-tolerant (OTR) cells.

 **Supplementary Figure 8.** Un-cropped images of the western blots for PPAT (A), GART (B), PFAS (C), PAICS (D), ATIC (E), AMPD2 (F), and ADSS (G) in H1975 osimertinib-tolerant (OTR) cells treated with LNA inhibitors against miR-21-5p (Anti-miR-21-5p) and a scrambled control (Anti-miR-control).

**Supplementary Figure 9.** Un-cropped images of the western blots for MTHFD1 (A,C) and MTHFD2 (B,D) in H1975 parental cells, osimertinib-tolerant (OTR) cells, and OTR cells treated with LNA inhibitors against miR-21-5p (Anti-miR-21-5p) or a scrambled control (Anti-miR-control).

 **Supplementary Figure 10.** Un-cropped images of the western blots for PPAT (A), GART (B), and PAICS (C) in H1975 parental cells treated with LNA inhibitors against miR-21-3p, miR-21-5p, or scrambled control.

**Supplementary Figure 11.** Un-cropped images of the western blots for p-AMPK (A), AMPK (B), and β-actin (C) in H1975 parental cells, OTR cells, OTR cells treated with the LNA inhibitor against a miRNA control (Anti-miR-control), and miR-21-5p (Anti-miR-21-5p).

**Supplementary Figure 12.** Un-cropped images of the western blots for p-AMPK (A), AMPK (B), p-ACC (C), and ACC (D) in H1975 cells treated with the LNA inhibitor against miR-21-3p (Anti-miR-21-3p), miR-21-5p (Anti-miR-21-5p), and a miRNA control (Anti-miR-control). Total AMPK and total ACC were used as loading controls.

**Supplementary Figure 14.** Un-cropped images of the Western blots for NRF2 (A) in H1975 cells treated with 1 mM AICAR for 0, 1, 2, 4, 6, 8, and 24 hours. β-Actin (B) was used as a loading control.

 **Supplementary Figure 16.** Un-cropped images of the Western blots for NRF2 (A) and β-Actin (B) in H1975 parental cells treated with vehicle, AICAR (1 mM), osimertinib (1 μM), or both for four hours.
